# Biological evaluation, molecular modeling and dynamics simulation of phenanthrenes isolated from *Bletilla* *striata* as butyrylcholinesterase inhibitors

**DOI:** 10.1038/s41598-022-17912-7

**Published:** 2022-08-11

**Authors:** Yi Liu, Yanbei Tu, Yunyao Kang, Chao Zhu, Chuanhai Wu, Gang Chen, Zerong Liu, Yanfang Li

**Affiliations:** 1 School of Chemical Engineering, Sichuan University, Chengdu, 610065 China; 2 School of Pharmacy, Jiangsu University, Zhenjiang, 212013 Jiangsu China; 3 Central Nervous System Drug Key Laboratory of Sichuan Province, Luzhou, 646106 China; 4 Sichuan Credit Pharmaceutical CO., Ltd., Luzhou, 646106 China

**Keywords:** Enzymes, Drug discovery, Plant sciences

## Abstract

As part of our continuous studies on natural cholinesterase inhibitors from plant kingdom, the 95% ethanol extract from tubers of *Bletilla* *striata* showed promising butyrylcholinesterase (BChE) inhibition (IC_50_ = 8.6 μg/mL). The extracts with different polarities (petroleum ether, ethyl acetate, n-butanol, and water) were prepared and evaluated for their inhibition of cholinesterases. The most active ethyl acetate extract was subjected to a bioassay-guided isolation and afforded twenty-two bibenzyls and phenanthrenes (**1**–**22**). All isolates were further evaluated for their BChE inhibition activity, and five phenanthrenes presented promising capacity (IC_50_ < 10 μM). Further kinetic studies indicated their modes of inhibition. Compounds **6**, **8**, and **14** were found to be mixed-type inhibitors, while compounds **10** and **12** could be classified as non-competitive inhibitors. The potential interaction mechanism of them with BChE was demonstrated by molecular docking and molecular dynamics simulation, showing that they could interact with catalytic active site and peripheral anionic site of BChE. These natural phenanthrenes provide new scaffold for the further design and optimization, with the aim to discover new selective BChE inhibitors for the treatment of AD.

## Introduction

Alzheimer's disease (AD) is a serious neurodegenerative disorder associated with memory loss and progressive impairment in cognitive functions. The estimated number of AD patients worldwide is forecasted to elevate to 130 million in 2050^1^. The amyloid beta peptide, phosphorylated tau protein, oxidative stress and dysfunction in cholinergic neurotransmission are all responsible for pathogenesis of AD^2^.

Cholinergic dysfunction directly contributes to cognitive decline. Treatment of cholinergic dysfunction is currently the most effective therapeutic strategy. There are two cholinesterases in the brain. Acetylcholinesterase (AChE) can catalyze acetylcholine hydrolysis, and its inhibition will increase the levels of acetylcholine in the brain, so as to improve cholinergic functions in AD patients. Compared with AChE, butyrylcholinesterase (BChE) plays a supportive role in cholinergic neurotransmission, but its level and activity significantly increase in the late stage of AD, so it is considered to be a promising drug target, therefore, the selective-BChE or dual ChE inhibitors may be more effective for advanced AD patients^3,4^.

Most of the drugs currently available for the treatment of AD, such as dual cholinesterase inhibitor rivastigmine, AChE inhibitors galanthamine and huperzine A, are derived from natural products^5^. In this regard, many research groups have focused on naturally-occurring compounds from plants as potential sources of new or more effective ChE inhibitors^6^. Several kinds of secondary metabolites of plants are reported potency against ChEs^7–9^.

*Bletilla* *striata* (Thunb.) Reichb. F (Chinese Ground Orchid), a Traditional Chinese Medicine used for the treatment of gastrointestinal mucosal injury, pulmonary edema, bleeding, hematemesis, hemoptysis and skin cracks, is widely distributed in China, Japan, Korea, Vietnam, and Thailand^10,11^. In addition, it also widely used for the treatment of swellings, sores, and chapped skin due to its efficacy of dispersing swelling and promoting tissue regeneration^12,13^. Previous phytochemistry studies have revealed that polysaccharides, bibenzylsand phenanthrenes are the main constituents of this species^14,15^. Triterpenoids, steroids, anthraquinones and organic acids were also detected in *B.* *striata*^16–19^. Some of the secondary metabolites exhibited potent cytotoxicity, antimicrobial, and anti-inflammatory activities^20–22^. In our continue effort to search for natural cholinesterase inhibitors from plant kingdom^23–25^, the 95% ethanol extract of *B.* *striata* tubers displayed potent inhibitory effects against BChE, which is consistent with one Chinese patent^26^. Recently, we have reported ChEs and beta-amyloid aggregation inhibitory activity of several simple phenanthrenes from *Cremastra* *appendiculata*^27^. The rich source of phenanthrenes in *B.* *striata* and its promising inhibitory activity toward BChE prompted us to further investigate its anti-AD potential. So, the anti-ChEs capacity of different solvent extracts and phytochemicals derived by a bioassay-guided isolation was investigated. Kinetic, molecule docking and molecule dynamic simulations were adapted to reveal the interaction mechanism between bioactive phytochemicals with BChE.

## Results and discussion

### Chemistry

**Figure 1:**
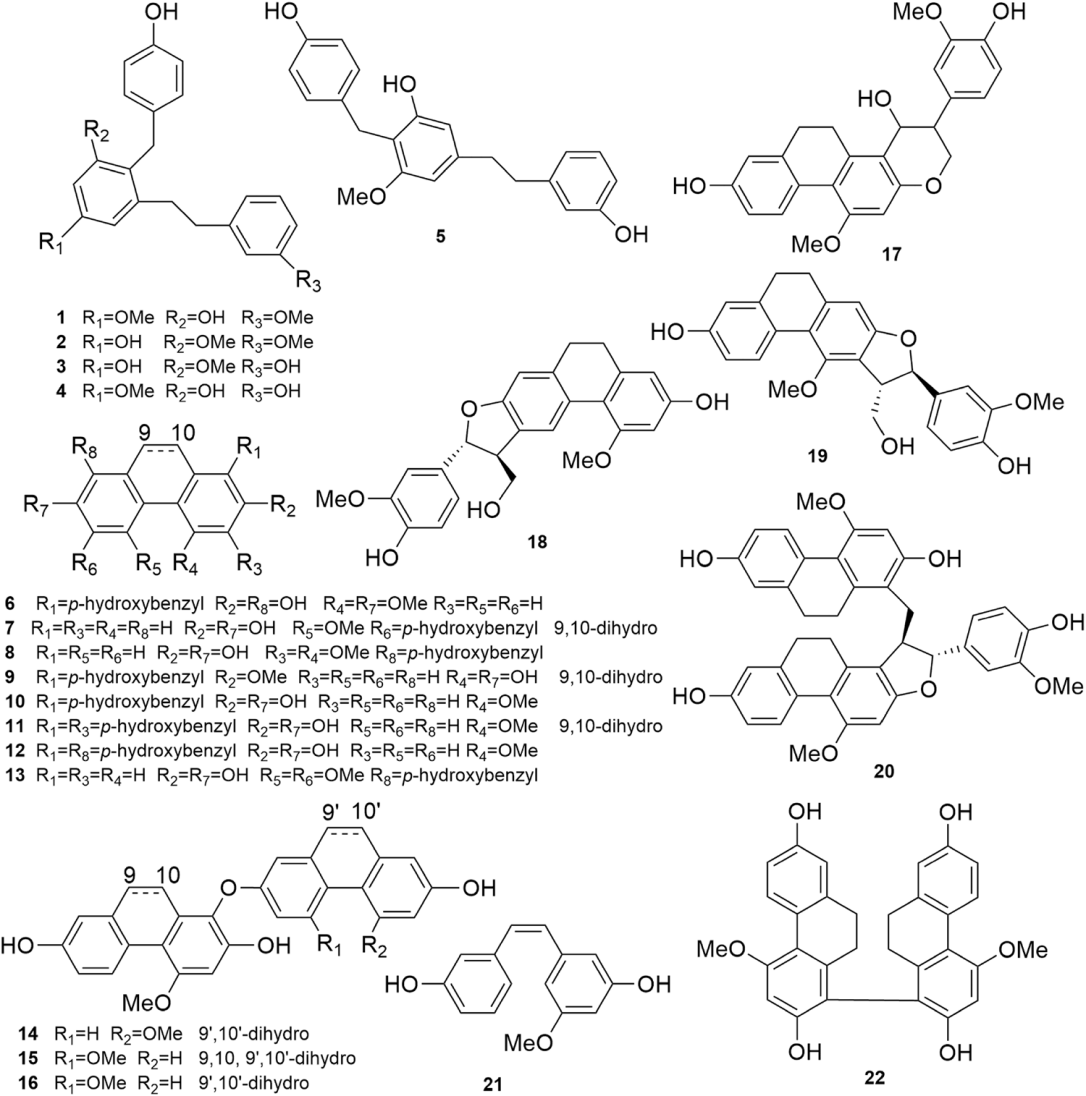
The structures of twenty-two compounds.

Isolation scheme of *B.* *striata* tubers was shown in Fig. S1. Two ChEs inhibition of 95% ethanol, petroleum ether, ethyl acetate, *n*-butanol, and water extracts of *B.* *striata* tubers were investigated and presented in Table S1. The results were expressed in IC_50_ (the half-inhibition concentration μg/mL). According to the results, EtOAc extract displayed the most potent BChE inhibition and the highest selectivity in comparison to AChE (IC_50 AChE_ = 224.0 μg/mL, IC_50 BChE_ = 2.3 μg/mL). Due to the potent BChE inhibition, EtOAc extract was subjected for further bioassay-guide isolation to explore its active phytochemicals. Consequently, 22 compounds (**1**–**22,** Fig. 1) were obtained by following combination of chromatographic techniques and assigned as phenanthrenes and bibenzyls by ^1^H-, ^13^C-, 2D NMR, HR ESI-MS spectrum and HPLC chromatogram analysis.

As previously reviewed, a fairly large number of naturally occurring phenanthrenes have been reported from higher plants, most of them were isolated from the species of the Orchidaceae family, in 49 species: in particular *Dendrobium*, *Bulbophyllum*, *Eria*, *Maxillaria*, *Bletilla*, *Coelogyna*, *Cymbidium*, *Ephemerantha,* and *Epidendrum*^28^. Our present finding concerning the chemical constituents goes in line with previous reports on *B.* *striata*. Noticeably, compounds **18** and **20** are two new phenanthrenes^29^, while compounds **17**, **19** and **21** were obtained from this species for the first time. Notably, the biological activities of most of isolates have not been reported.

### Biological activity evaluation

For all isolated compounds, inhibitory activities on two ChEs in comparison to the references galanthamine and tacrine were detected. The initial tests were achieved at a concentration of 25 μg/mL (final concentration in the reaction system) and all of them exhibited no or weak AChE inhibitory activity. Interestingly, BChE was found to be much more sensitive than the AChE towards the evaluation (Table 1). Subsequently, IC_50_ values towards BChE were determined. Sixteen compounds displayed moderate inhibition with IC_50_ values lower than 100 μM, and most of them (except **3** and **11**) demonstrated better inhibition than the standard galanthamine. More importantly, five phenanthrenes (**6**, **8**, **10**, **12**, and **14**) displayed significant BChE inhibitory effect (IC_50_ < 10 μM) and their selectivity towards BChE was superior to that of tacrine.

**Table 1:** Inhibition activity of phytochemicals isolated from *B.* *striata* against two cholinesterases.

Compounds	% of inhibition at 25 μg/mL		IC_50_ for BChE^b^ (μM)	Selectivity ratio
	AChE^b^	BChE^b^		
**1**	48.1 ± 6.3	66.2 ± 3.4	40.5 ± 5.6	> 1.7
**2**	16.3 ± 3.8	67.7 ± 0.3	33.5 ± 3.7	> 2.1
**3**	5.0 ± 1.5	51.3 ± 2.0	80.3 ± 5.2	–
**4**	0.9 ± 0.8	39.3 ± 2.3	–	–
**5**	2.6 ± 2.8	22.6 ± 2.1	–	–
**6**	20.4 ± 4.5	85.2 ± 2.9	6.4 ± 0.2	> 9.5
**7**	9.6 ± 2.6	65.7 ± 0.7	34.0 ± 1.4	> 2.1
**8**	18.5 ± 1.7	70.0 ± 1.0	5.2 ± 0.4	> 13.1
**9**	15.2 ± 3.6	72.8 ± 3.4	16.7 ± 2.0	> 4.3
**10**	19.1 ± 3.8	96.6 ± 1.2	2.1 ± 0.3	> 34.7
**11**	20.1 ± 3.5	53.1 ± 1.2	44.6 ± 4.1	> 1.2
**12**	16.1 ± 5.0	95.4 ± 0.3	2.3 ± 0.4	> 23.1
**13**	9.9 ± 4.7	75.7 ± 1.1	16.7 ± 2.4	> 4.0
**14**	6.8 ± 1.6	69.0 ± 2.5	8.1 ± 0.5	> 6.8
**15**	8.4 ± 3.1	64.0 ± 2.6	17.9 ± 4.7	> 2.9
**16**	4.9 ± 3.2	64.3 ± 2.4	12.1 ± 3.4	> 4.3
**17**	5.5 ± 1.8	21.8 ± 3.1	–	–
**18**	5.7 ± 2.8	31.6 ± 2.8	–	–
**19**	3.3 ± 1.8	61.2 ± 1.3	35.8 ± 9.2	> 1.7
**20**	5.1 ± 4.0	8.0 ± 2.4	–	–
**21**	5.2 ± 3.2	29.1 ± 1.3	–	–
**22**	13.3 ± 2.9	56.7 ± 2.0	42.2 ± 5.1	> 1.2
Galantamine^a^	94.8 ± 0.9	64.2 ± 0.6	46.3 ± 5.8	0.1
Tacrine^a^	–	–	0.0101 ± 0.0005	4.3

Selectivity ratio: % of inhibition at 25 μg/mL _AChE_/% of inhibition at 25 μg/mL _BChE_.

^a^Positive control.

^b^Data are the mean ± SD of three independent experiments. AChE from electric eel and BChE from equine Serum.

Notably, phenanthrenes **6**–**13** were found to inhibit BChE with IC_50_ values ranging from 2.1 to 44.6 μM. By comparing their structure and activity, it should be noted that the presence of *p*-hydroxybenzyl attenuated the inhibitory capacity. Compound **10**, bearing two hydroxyls and one methoxy, along with the *p*-hydroxybenzyl substituent at C1 position on phenanthrene skeleton, presented the strongest inhibition (IC_50_ = 2.1 μM) and the highest selectivity towards BChE (SI > 34.7) among the compounds with similar structures. In addition, among the five phenanthrenes dimers, **14**–**16** displayed attractive activity, while **20** exerted weak inhibition potency and **22** presented moderate efficacy, indicating the influence of the linkage of two monomers and the substitution position of hydroxy/methoxy on BChE inhibition. In contrast, two phenanthrofurans (**18** and **19**) demonstrated obvious difference potencies, and it is speculated that the substitution sites of methoxy affect whether small molecules can penetrate deep to the bottom of the active pocket and bind to the residues of key amino acids. It is worth noting that all isolated bibenzyls showed poor anti-BChE capacity compared to phenanthrenes. Collectively, the above results demonstrated that phenanthrenes should be the main BChE inhibitory components of *B.* *striata*.

### Kinetic study of selective BChE inhibitors

**Figure 2:**
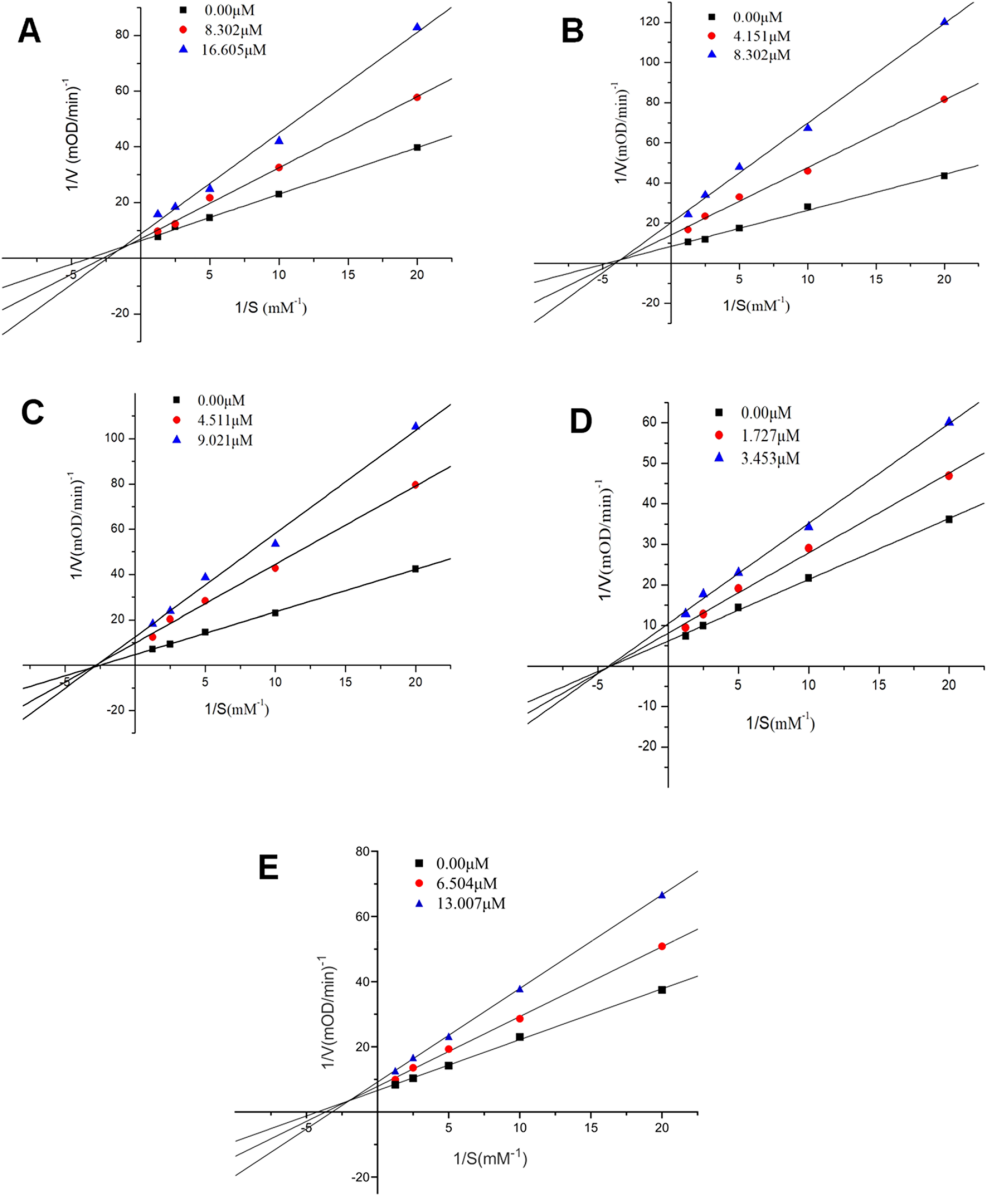
Lineweaver–Burk plots of five potent inhibitors against BChE. **6** (**A**), **8** (**B**), **10** (**C**), **12** (**D**) and **14** (**E**). ([S] concentration of BTCI).

To study mechanism of BChE inhibition, five most potent inhibitors were selected to further study the kinetics using Lineweaver–Burk plots. As shown in Fig. 2, with the increasing concentrations of compounds **6**, **8** and **14**, the V_max_ were decreased and K_m_ were increased, these results suggested that these three compounds were the mixed-type inhibitors where they can bind to the active site of BChE. Furthermore, with the increasing concentrations of **10** and **12**, the V_max_ were decreased and K_m_ kept as a constant value, therefore, **10** and **12** could be classified as noncompetitive inhibitors of BChE where both of them can bind to the enzyme or enzyme-substrate complex. The K_i_ values of five BChE inhibitors were estimated by the secondary plots of Lineweaver–Burk plots and determined as 14.0 μM, 4.7 μM, 5.4 μM, 6.6 μM, and 10.9 μM, respectively.

### Molecular docking study

**Figure 3:**
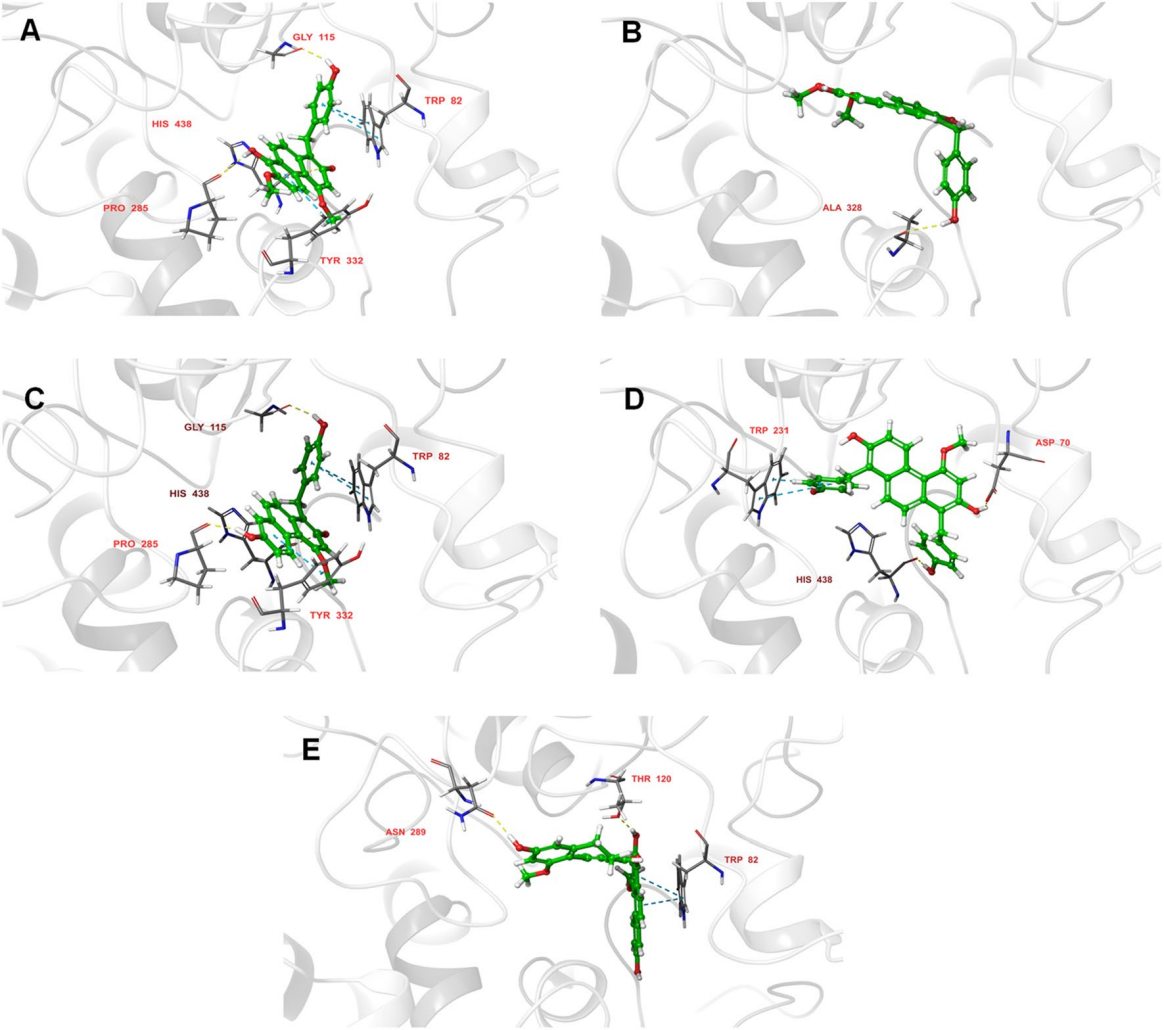
Interacting residues of BChE with five potent inhibitors (**A** for **6**, **B** for **8**, **C** for **10**, **D** for **12**, **E** for **14**) were shown in sticks colored by atom type, the carbon in green, hydrogen in white, nitrogen in blue, and oxygen in red. Interactions included non-covalent bonds and π interaction, the hydrogen bonds in yellow, π-π stacking in blue.

To further reveal the interaction modes of five inhibitors, the molecular docking was implemented using the Glide module of Schrödinger. The active site of BChE consists of a peripheral anion site (Asp70), a choline binding pocket (Trp82), an acyl-binding pocket (Val288, Leu286, Trp231) and catalytic residues (Ser198, His438 and Glu325)^30^. Trp82 and Ser198, in particular, are considered as key amino acid residues^31^. As depicted in Fig. 3C, compound **10** bind to the active pocket of BChE via multiple hydrogen bonds with His438, Pro285, Gly115 and π–π stacking with Tyr332 and Trp82. This finding is in good agreement with the results of kinetic study and give a reasonable explanation for its highest potency against BChE. In addition, **10** and the other four inhibitors shared similarities in binding to the enzyme, with the hydroxyl groups at the C-2 and C-7 positions (except for the C-2 hydroxyl group of **8**) establishing H-bonds with residues in the active pocket of the enzyme (Fig. 3). Accordingly, compound **6** was less active owing to methoxy substitution at C-7.

As mentioned above, the hydroxyl groups at C-2 and C-7 of the phenanthrene skeleton are essential for inhibitory activity. Interestingly, the *p*-hydroxybenzyl moiety of four ligands (**6**, **8**, **10** and **12**) engaged in hydrogen bond and π–π stacking interactions with Trp82 and His438 (Fig. 3). These observations indicated that the substituents with the donor or receptor of hydrogen bond on the C-1 and C-8 play a key role in improving binding affinity of phenanthrenes. Taken together, these docking results may explain why five phenanthrenes display the highest affinity for BChE in all phenanthrenes and bibenzyls.

### Molecular dynamic simulations

**Figure 4:**
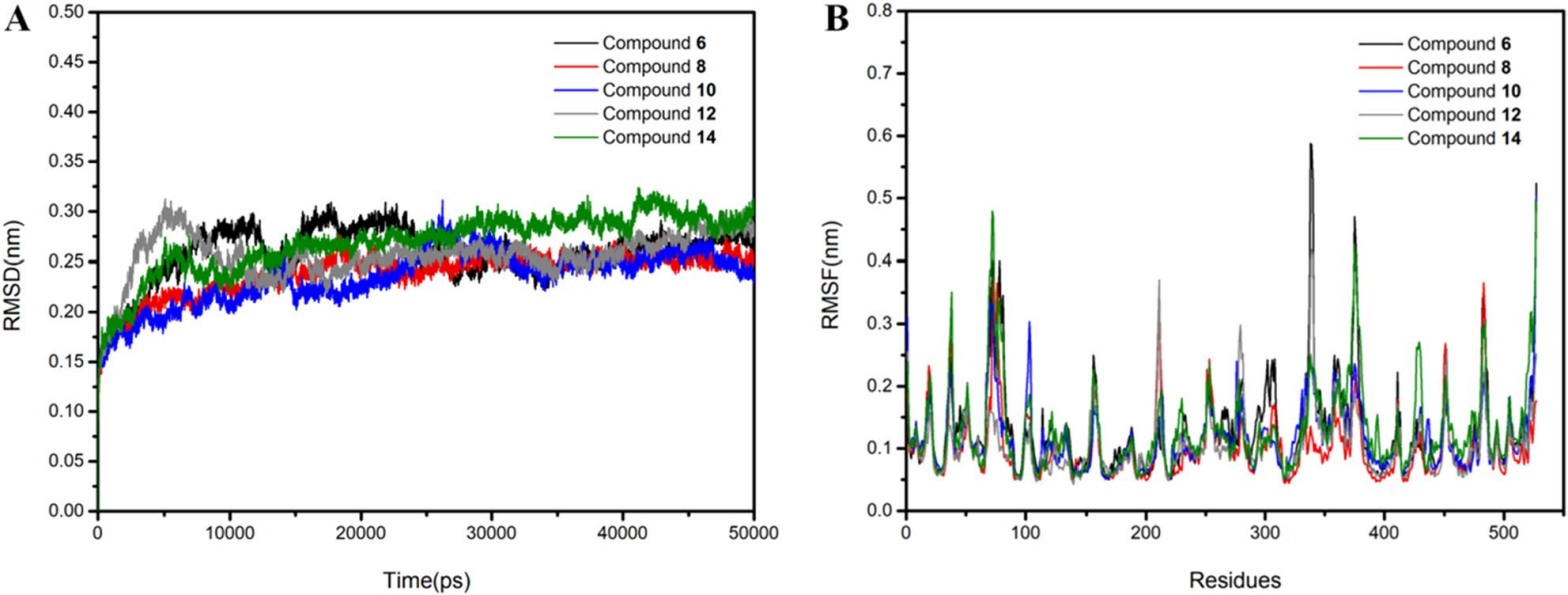
The RMSD (**A**) and RMSF (**B**) of five potent inhibitors with BChE.

Based on these promising results obtained from molecule docking, further insights concerning the dynamic behavior and binding mechanistic information were demonstrated by molecular dynamics simulation (MD) in GROMACS. The docking pose of five inhibitors with BChE (PDB:4TPK) for 50 ns were collected to generate RMSD (Root mean squared deviation) and RMSF (Root mean squared fluctuation) graphs (Fig. 4).

RMSD of five inhibitors were plotted to verify molecular interactions and structural stability. As depicted in Fig. 4A, all five complexes possessed stable RMSD values which were all less than 0.35 nm, indicating that the structure of each complex was relatively stable. The RMSD trajectory of BChE-**6** reached equilibrium within approximately 30 ns and remained until the end of the simulation. While the BChE-**8** system performed best with its RMSD rising gradually before 20 ns and stabilizing in subsequent simulations without significant fluctuations, stabilizing around 0.23 nm. Also, BChE-**10** system showed a gradual increase in RMSD from the beginning, a decreasing trend at about 15 ns, and was essentially stable after 30 ns. Meanwhile, the BChE-**12** system had the largest fluctuations at the beginning, but it started to stabilize around 20 ns until the end of the simulation. In particular, the BChE-**14** system exhibited an increasing trend at the beginning of the simulation, then largely stabilized from approximately 20 ns onwards and fluctuated in the 0.25–0.30 nm range. In a word, the RMSD plots indicated that the five inhibitors remained stable during the MD simulation by forming molecular interactions with surrounding residues.

RMSF can be used to calculate the fluctuation amplitude of a single residue and measure the degree of freedom of atomic motion in the process of dynamic simulation, and thus reflecting the flexibility of protein region motion. The RMSF of the C-alpha for the complexes of each inhibitor with BChE were calculated and the results obtained by calculating RMSF of the five systems in the equilibrium state are shown in Fig. 4B. The average fluctuation range was in the range of 0.1–0.6 nm. In short, no significant conformational changes in protein and molecular structure were observed during simulations and the vital residues including Trp82 and Phe329 in them were quite stable. The average RMSF values obtained for the active molecules also indicated good protein flexibility. Accordingly, all five inhibitors exhibited a good stability and inhibitory activity.

**Figure 5:**
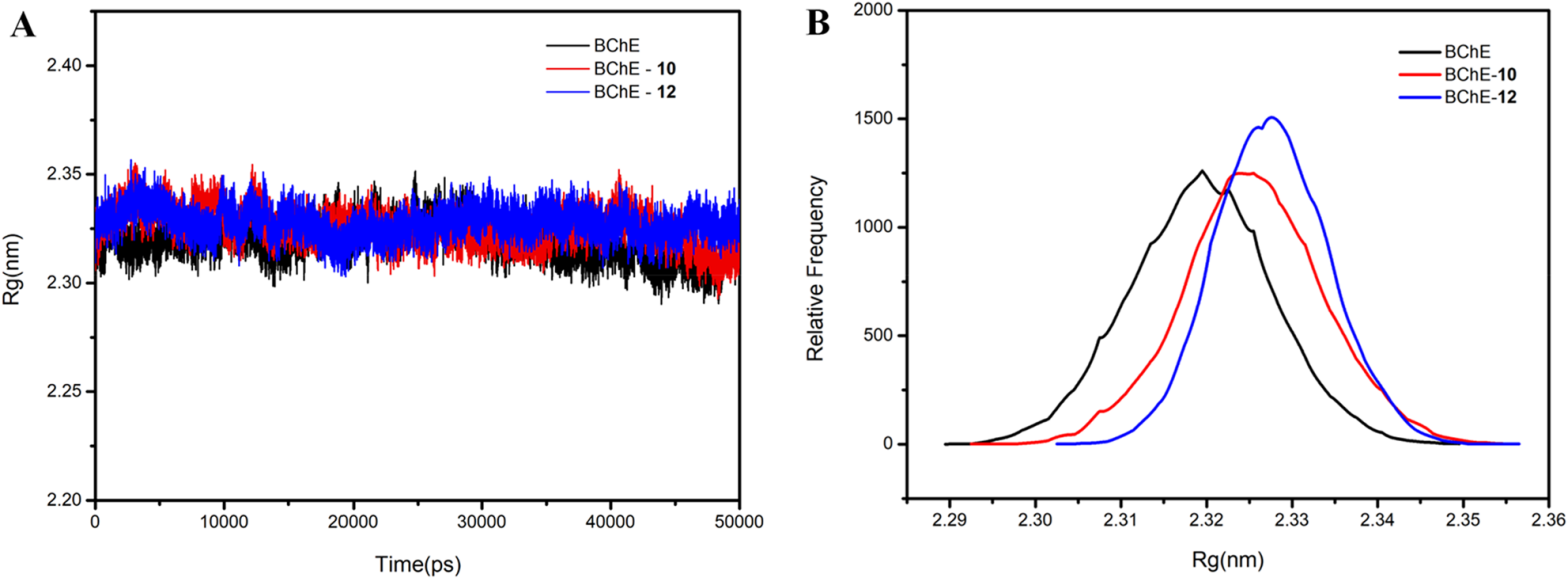
The Rg (**A**) and relative frequency (**B**) of **10** and **12** with BChE.

The radius of gyration (Rg) correlated with the relationship between the atomic mass and the center of gravity of a protein, which can be used to evaluate the structural compactness of the protein docking complexes. The stably folded proteins showed a relatively steady value of Rg, while the perturbation of protein conformation showed fluctuations in Rg values. As shown in the Fig. 5A, the Rg value of both (**10** and **12**) remain stable in the range of 2.30–2.35 nm. From the relative frequency analysis (Fig. 5B), the Rg values of protein complexes were slightly higher than those of individual BChE. But the offset value of Rg is only 0.01–0.015 nm. This suggested that the protein BChE became marginally looser bound to the compounds **10** and **12**, but the effect is limited.

**Figure 6:**
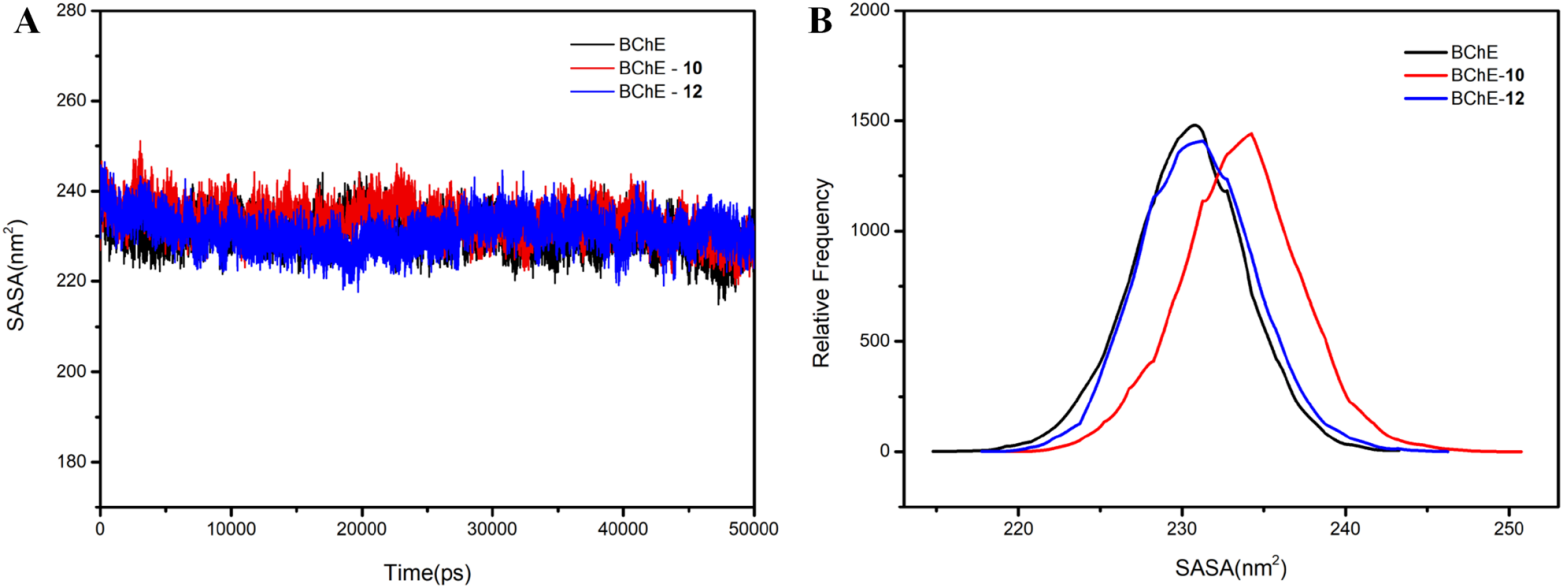
The SASA (**A**) and relative frequency (**B**) of **10** and **12** with BChE.

The solvent-accessible surface area (SASA), an important parameter to describe the hydrophobicity of proteins, was observed and shown in Fig. 6A. Results showed that SASA value of both (**10** and **12**) remain relatively stable in the range from 220 to 245 nm^2^ in the simulation time 0–50 ns. The relative frequency analysis (Fig. 6B) indicated that the SASA of two complexes were attained increasing compared with individual BChE. Notably, the SASA of molecule **10** was significantly increased compared to the slight SASA increase of molecule **12**. This implied that the binding of **10** led to the opening of hydrophobic cavity of BChE, while **12** had little effect on the hydrophobicity of BChE.

### In silico ADME prediction

To evaluate drug-like properties of compounds, the ADME (absorption, distribution, metabolism and excretion) properties of five inhibitors were predicted in silico using the QikProp v. 5.5 (Schrödinger). As showed in Table 2, five molecules performed well in the matter of acceptable absorption percentage, polar surface area, hydrogen bond acceptor/donor and oral bioavailability. It is important to consider that CNS (central nervous system) drugs usually exhibit low molecular weights (MWs), usually in the range of 400–600 Da or below. Marketed CNS-acting drugs have been reported to show a mean MW of 310 Da^32^. In the present study, all five inhibitors presented MWs within the established intervals. QPlogP_o/w_ predicts the water solubility of the molecules. The QPlogPo/w values of five ligands were lower than 5.311, signifying that they had good water solubility. Rather, the QPlogBB values which predict the ability of molecules to cross the blood–brain barrier were in an ideal range, indicating that five molecules could enter the central nervous system via oral administration. In addition, human oral absorption (HOA%) values exceed 90%, implying that these molecules were fully absorbed in the gastrointestinal tract.

**Table 2:** ADME prediction results of five potent inhibitors (IC_50 BChE_ < 10 μM).

Compounds	**6**	**8**	**10**	**12**	**14**	Galanthamine	Tacrine
CNS^a^	− 1	− 1	− 2	− 2	− 2	1	1
MW^b^	376.408	376.408	346.382	452.506	480.516	287.4	198.3
SASA^c^	596.087	604.374	578.639	634.062	756.242	509.3	419.9
volume^d^	1093.846	1102.818	1045.459	1235.779	1374.832	900	689
donor HB^e^	3.000	3.000	3.000	4.000	3.000	1	1.5
accpt HB^f^	3.750	3.750	3.000	3.750	4.250	5.2	2
QPlogPo/w^g^	3.726	3.724	3.477	4.207	5.311	2.01	2.50
QPlogS^h^	− 4.620	− 4.436	− 4.328	− 4.380	− 6.951	− 2.2	− 2.9
QPPCaco^i^	820	654	376	378	685	758	3374
QPlogBB^j^	− 0.763	− 0.996	− 1.145	− 1.270	− 1.163	0.38	0.10
metab^k^	6	6	5	7	8	4	3
QPlogKhsa^l^	0.496	0.431	0.431	0.594	1.043	0.01	0.02
%HOA^m^	100	100	93	100	96	90	100
PSA^n^	69.918	65.339	70.653	84.554	72.965	43.30	31.57
ROF^o^	0	0	0	0	1	0	0
ROT^p^	0	0	0	1	2	0	0

^a^Predicted central nervous system activity (− 2–2).

^b^Molecular weight of the molecule (130–725).

^c^Total Solvent Accessible Surface Area, in square angstroms, using a probe with a 1.4 Å radius (300.0–1000.0).

^d^Total solvent-accessible volume, in cubic angstroms, using a probe with a 1.4 Å radius (500.0–2000.0).

^e^Estimated number of hydrogen bonds that would be donated by the solute (0.0–6.0).

^f^Estimated number of hydrogen bonds that would be accepted by the solute (2.0–20.0).

^g^Predicted octanol/water partition coefficient (− 2.0–6.5).

^h^Predicted aqueous solubility (− 6.5–0.5).

^i^Predicted apparent Caco-2 cell (a model for the gut-blood barrier) permeability in nm/sec (< 25 poor, > 500 great).

^j^Predicted brain/blood partition coefficient (− 3.0–1.2).

^k^Number of primary metabolites (1–8).

^l^Prediction of binding to human serum albumin (− 1.5–1.5).

^m^Predicted qualitative Human Oral Absorption (> 80% is high, < 25% is poor).

^n^Van der Waals surface area of polar nitrogen and oxygen atoms (7.0–200.0).

^o^Number of violations of Lipinski’s Rule of Five (molecular weight < 500, QPlogPo/w < 5, number of hydrogen bond donor < 5, number of hydrogen bond acceptors HB < 10).

^p^Number of violations of Jorgensen's rule of three (QPlogS >  − 5.7, QPCaco > 22 nm/s, number of primary metabolites < 7).

## Conclusion

*Bletilla* species are well known as a rich source of bibenzyls, phenanthrenes and dihydrophenanthrenes^10^. The significant inhibition effect of several simple phenanthrene derivatives on BChE activity, antioxidant activity, and Aβ self-induced aggregation have been reported in our previous work^27^. However, no inhibition of ChEs by phenanthrenes derived from the tubers of *B.* *striata* has been documented. In current study, we focused on discovery of anti-cholinesterase phytochemicals from *B.* *striata* and their biological activity evaluation, molecular modeling and dynamics simulation*.* Using a bioassay-guided isolation approach, 22 compounds were obtained from the promising ethyl acetate extract of *B.* *striata*, and further structure determination categorized them into two major groups (bibenzyls and phenanthrenes). This is the first report of phenanthrenes and bibenzyls with ChEs inhibitory activity in this species. It is noteworthy that the potency of phenanthrenes is higher than that of bibenzyls. Interestingly, phenanthrenes bearing *p*-hydroxybenzyl showed higher potency than simple hydroxy or methoxy-substituted phenanthrenes, since the presence of *p*-hydroxybenzyl favored their binding with key amino acid residues in active pocket of BChE. Five phenanthrenes (**6**, **8**, **10**, **12** and **14**) presented the most potent BChE inhibitory activity and exhibited mixed/noncompetitive type of inhibition, which was supported by the adopted in silico studies. Their potential interaction mechanism with BChE was demonstrated by molecular docking and molecular dynamics simulation. In summary, current findings suggested that phenanthrene compounds from *B.* *striata* have significant potential to be developed as novel anti-AD lead compounds.

## Methods

### General experimental procedures

NMR spectra were recorded on Bruker AVANCE III-400 spectrometer using CDCl_3_ and CD_3_OD as solvents. Chemical shifts were reported in parts per million (ppm, δ) downfield from tetramethylsilane. HR-TOF-MS were determined by a quadruple time of flight (Q-TOF) premier spectrometer coupled with an ESI source (Waters Co., Ltd, Milford, USA). Column chromatography procedures were performed using silica gel (200–400 mesh, Qingdao Marine Chemical Ltd., China), MCI resin CHP 20P (Mitsubishi Chemistry Co. Ltd., Japan), Toyopearl gel HW-40F (TOSOH Co., Ltd., Japan), and Sephadex LH-20 (25–100 μm, Pharmacia Fine Chemical Industries Co. Ltd., Sweden). Thin-layer chromatography (TLC) was performed using silica gel GF_254_ precoated plates (Qingdao Marine Chemical Ltd., China). After spraying with a color reagent (10% vanillin-H_2_SO_4_ and 10% H_2_SO_4_ in EtOH), heating revealed the spots. All reagents were purchased from commercial sources without further purification.

### Plant material

The dried tubers of *B.* *striata* were purchased from the local herbal market in Chengdu (Sichuan Province, China) in July 2015, and identified by Dr. Wenbing Liu. A voucher specimen (NO. P00035) has been deposited in the Department of Pharmaceutics and Bioengineering, School of Chemical Engineering, Sichuan University, Chengdu, China. All the herbs tested in the research are permitted and legal for trade, commercialization in China. Therefore specific permission was not needed from the Local Authority. All methods were carried out in accordance the Chinese Pharmacopoeia (2020 edition).

### Extraction, bioactivity-guided isolation and characterization

The dried and powdered tubers of *B*. *striata* (2 kg) were extracted by 95% ethanol (5 × 10 L) in warm water bath (50 °C). Then, ethanol was evaporated by rotary evaporator, yielding 392.3 g dark residue. The residue was suspended in water and partition with PE, EtOAc and water saturated *n*-BuOH respectively, 28.5 g, 60.25 g and 188.9 g resulting extracts were obtained. The EtOAc extract presented more potent BChE inhibition was further subjected to silica gel CC (60 × 350 mm) and gradient eluting with PE/acetone (v/v = 10:1–0:1), eleven subfractions (Fr. A–K) were obtained. Subfractions J (12.84 g) and K (10.08 g) were subjected to the MCI resin CC (70 × 320 mm) and gradient eluted by acetone (v/v = 10:90–100:0 and v/v = 40:60–75:25) to afford Fr.J1–J11 and Fr.K1–K9 respectively. Fr.J10 (2.39 g) was subjected to the HW-40F CC (24 × 930 mm) and eluting with methanol to give subfractions Fr.J10a–J10f. Further isolation by MCI resin and HW-40F gel CC, and preparative TLC (PTLC) to give 1-(*p*-hydroxybenzyl)-4, 7-dimethoxyphenanthrene-2,8-diol (**6**)^33^, shanciol F (**17**)^34^, 3-(4-hydroxybenzyl)-4-methoxy- 9,10-dihydrophenanthrene-2,7-diol (**7**)^35^, bleformin A (**8**)^36^, shancidin (**9**)^37^, 1-[(4-hydroxyphenyl)methyl]-4-methoxy-2,7-phenanthrenediol (**10**)^38^, 3,3′-dihydroxy-4-(4-hydroxybenzyl)-5-methoxybibenzyl (**5**)^37^, 3′,5-dihydroxy-2-(4-hydroxybenzyl)-3-methoxybibenzyl (**3**)^38^, isoarundinin II (**4**)^39^, 2,7-dihydroxy-1,3-bi(p-hydroxybenzyl)-4-methoxy-9,10-dihydrophenanthrene (**11**)^40^, pholidotol D (**21**)^41^, flavanthrin (**22**)^42^, 1,8-bis(4-hydroxybenzyl)-4-methoxyphenanthrene-2,7-diol (**12**)^43^, bleformin B (**13**)^36^, bletilol D (**18**)^29^. Fr. J11 was further separated by HW-40F CC to give two subfractions, gymconopin D (**1**)^38^, bulbocol (**2**)^44^ and 9-(4′-hydroxy-3′-methoxyphenyl)-10-(hydroxymethyl)-11-methoxy-5,6,9, 10-tetrahydrophenanthro[2,3-b] furan-3-ol (**19**)^45^ were obtained from the latter by repeated PTLC. Bletilol E (**20**)^29^, blestrin D (**14**)^46^, blestrin C (**16**)^46^, and blestrin A (**15**)^47^ was obtained from Fr. K7 by HW-40F CC and repeated PTLC.

### Enzyme inhibition

Cholinesterase inhibitory activities were evaluated by a microplate assay based on the modified Ellman's method^48^. Firstly, 25 μL of 15 mM ATCI/BTCI (in ultrapure water) were added into the wells of plates, then the 125 μL of 3 mM DNTB in PBS (pH 8.0), 65 μL PBS (pH 8.0) and 10 μL of various concentration samples (in methanol) were successively added to the well. After all the reagents added, the changes of the absorbance were detected eight times at 405 nm every 45 s as the spontaneous hydrolysis rate of ATCI/BTCI. Galantamine and tacrine (hydrochloride) were used as positive control.

After the determination of spontaneous hydrolysis rate of ATCI/BTCI, 25 μL of 0.226 U/mL ChE (AChE from electric eel and BChE from equine serum) in PBS (pH 8.0) were added to each well of the 96-well plates and then the plates were shaken 15 s under the medium speed. Finally, changes in the absorbance at 405 nm were determined by the method mentioned above to obtain the apparent enzymatic hydrolysis rate. The actual enzymatic hydrolysis rate was corrected by deducting the spontaneous hydrolysis rate of ATCI/BTCI. The enzymatic hydrolysis rate determined when 10 μL of methanol added to the well instead of the samples were set as blank. The concentration of the methanol in final reaction mixture is lower than 4% and has no influence on AChE/BChE. All the tests were achieved in triplicate and compounds showing more than 50% inhibition rate at 25 μg/mL (final concentration in the reaction system) were further determined the IC_50_ values. The corresponding IC_50_ measurement concentration were set based on the inhibition rate at 25ug concentration, ranging from 0.04 to 50 ug/ml. The least square method of inhibitor versus Response-variable slope (four parameters) model in GraphPad Prism 8.0 (GraphPad Software, USA) was used for calculation and fitting the IC50 values. The inhibition percentage of ChE (%) calculated using the following formula:

1 $$ {\text{Inhibition}}\left( \% \right) = \left( {1 - {\text{V}}_{{{\text{sample}}}} /{\text{V}}_{{{\text{blank}}}} } \right) \times 100\% $$

V_blank_ and V_sample_ represent the hydrolysis rate of the blank control and test sample respectively.

### Kinetic characterization of BChE inhibition

The Lineweaver–Burk plots analyses was performed to determine the inhibition mode of five potential inhibitors (IC_50_ < 10 μM). Five increasing concentrations of BTCI as a substrate in the presence of three different concentrations of compound were used in the kinetic measurement. The concentrations of the enzyme and DNTB used in the determination are 0.226 U/mL and 3 mM respectively. The inhibition constants (*K*_i_) of the inhibitors were determined by the secondary plots which constructed using the slopes of the Lineweaver–Burk plots.

### Docking study

The molecular docking of active compounds was performed using the Glide module of Schrödinger. The X-ray crystal structure of human BChE (PDB ID: 4TPK) was obtained from Protein Data Bank. The five compounds were prepared by LigPrep module. The Protein Preparation Wizard of Maestro was used to prepared the complex structure of BChE, which retain one protein conformation, supplement incomplete residues, add hydrogen and keep the active site ligand only. The pH value of Protein and Compounds were set as the default values 7.0 ± 2.0. Then we used the Receptor Grid Generation module to define the active site of the protein through the cocrystallized ligand, and it was excluded from the grid generation. The redocking of cognate ligand was performed to evaluate the reliability of the docking model and the RMSD value was 1.95 Å (< 2 Å), which means the docking model is credible. The other parameters were set at the default values in Schrödinger.

### Molecular dynamic simulations

To further understand the mode of action of the inhibitors in the biological environment as well as to test the stability of the complexes with the hit compounds, MD simulation was carried out on GROMACS 2019.5. It was employed to obtain the trajectories of five complexes, which were used to obtain root mean square deviations and fluctuations (RMSD/RMSF) thus to evaluate the stability of each complex. The molecular dynamics experiment of 10 and 12 were repeated three times.

The topology of the protein was generated by GROMACS using the GROMOS96 force field, and the topologies of the ligands were obtained from the Automated Topology Builder website at https://atb.uq.edu.au/index.py. To maintain the periodic boundary conditions, the minimum distance between the protein atoms and the cell wall was set to 1 Å throughout the MD simulation process with the cubic cell. The simple point charge solvent model (spc216) was employed to solvate the system, and the species of the solvent was water. The processed system might not be in the electrically neutral state, and the charge of the system could be balanced by adding counter-ions such as Cl^−^ or Na^+^ to achieve the physiologically neutral state. The energy of the system was minimized to eliminate steric clashes, create an index and modify the .mdp file for the thermal coupling group as well as the subsequent position limitation. The position restraint dynamic under NVT (constant volume) and NPT (constant pressure) conditions at 300 K were brought into operation by a V-rescale which was modified by a Berendsen thermostat. The Particle Mesh Ewald method was employed to compute the long-range Coulombic and Lennard–Jones interaction energies for the protein–ligand interactions and the ligand dynamic. Different modules in the GROMACS package were brought into effect to analyze the results of the MD simulation to acquire the RMSD and RMSF. The simulation method used in this study is the same as that previously described in our previously published study^49^.

### ADME prediction

ADME properties are all used to assess the drug-forming properties of drugs. QikProp is a module in Schrödinger for predicting ADME properties of molecules. QikProp contains two basic models, fast and normal. The results were obtained for a variety of properties including partition coefficients (QPlogP octanol/water), predicted water solubility (QPlogS), percentage oral absorption in humans, volume, hydrogen bond donors and acceptors, etc. Forty-four properties of the molecule can be obtained after prediction. Before performing the QikProp calculations, molecular pre-processing was completed and the parameters were set as default.

### Statistical analysis

All analyses were carried out in triplicates. Data obtained were presented as mean and standard deviation values (S.E.M.). The IC_50_ values were calculated with GraphPad Prism 8.0.

## Supplementary Information

Supplementary Information.

## Data Availability

All data is provided in the manuscript or supplementary material.

## Abbreviations

AD: Alzheimer’s disease
AChE: Acetylcholinesterase
BChE: Butyrylcholinesterase
ChEs: Cholinesterases
ADME: Absorption, distribution, metabolism and excretion
CNS: Central nervous system
CAS: Catalytic active site
MD: Molecular dynamics simulation
RMSD: Root mean squared deviation
RMSF: Root mean squared fluctuation
Rg: Radius of gyration
SASA: Solvent-accessible surface area

## References

[1] Prince, M., Comas-Herrera, A., Knapp, M., Guerchet, M. & Karagiannidou, M. *World**Alzheimer**Report**2016:**Improving**Healthcare**for**People**Living**with**Dementia:**Coverage,**Quality**and**Costs**Now**and**in**the**Future* (2016).

[2] Hampel H (2018). The cholinergic system in the pathophysiology and treatment of Alzheimer's disease. Brain.

[3] Brus B (2014). Discovery, biological evaluation, and crystal structure of a novel nanomolar selective butyrylcholinesterase inhibitor. J. Med. Chem..

[4] Coban G (2016). 1H-benzimidazole derivatives as butyrylcholinesterase inhibitors: Synthesis and molecular modeling studies. Med. Chem. Res..

[5] Williams P, Sorribas A, Howes M-JR (2011). Natural products as a source of Alzheimer's drug leads. Nat. Prod. Rep..

[6] Deshpande P, Gogia N, Singh A (2019). Exploring the efficacy of natural products in alleviating Alzheimer’s disease. Neural Regen. Res..

[7] Ortiza JE, Garrob A, Pignic NB (2018). Cholinesterase-inhibitory effect and in silico analysis of alkaloids from bulbs of Hieronymiella species. Phytomedicine.

[8] Namdaung U (2018). 2-Arylbenzofurans from Artocarpus lakoocha and methyl ether analogs with potent cholinesterase inhibitory activity. Eur. J. Med. Chem..

[9] de Souza LG (2016). Coumarins as cholinesterase inhibitors: A review. Chemico-Biol. Int..

[10] He X (2017). *Bletilla*
*striata*: Medicinal uses, phytochemistry and pharmacological activities. J. ethnopharma..

[11] Wu JN (2005). An Illustrated Chinese Materia Medica.

[12] Luo Y, Diao H, Xia S, Dong L, Chen J, Zhang J (2010). A physiologically active polysaccharide hydrogel promotes wound healing. J. Biomed. Mater. Res..

[13] Ren, H., He, Y. & Yang, L. Advances of chemical constituents and pharmacological activities of *Bletilla**striata*. Asia Pac. Trad. Med. 134–140 (2009).

[14] Woo KW, Park JE, Choi SU, Kim KH, Lee KR (2014). Phytochemical constituents of *Bletilla*
*striata* and their cytotoxic activity. Helv. Chim Acta.

[15] Yu H, Dai B, Qian C, Ding Z, Jiang F, Jing B, Li M (2016). Antibacterial activity of chemical constituents isolated from fibrous roots of *Bletilla*
*striata*. J. Chin. Med. Mater..

[16] Yamaki M, Honda C, Kato T, Bai L, Takagi S (1997). The steroids and triterpenoids from *Bletilla*
*striata* (Natural Medicine Note). Nat. Med..

[17] Sun A, Liu J, Pang S, Lin J, Xu R (2016). Two novel phenanthraquinones with anti-cancer activity isolated from *Bletilla*
*striata*. Bioorg. Med. Chem. Lett..

[18] Yang L (2014). A new macrolide and six cycloartane triterpenoids from the tubers of *Bletilla*
*striata*. Biochem. System. Ecol..

[19] Han G (2001). Studies on the chemical constituents of *Bletilla*
*striata*. J. Pharm. Pract..

[20] Wang W, Meng H (2015). Cytotoxic, anti-inflammatory and hemostatic spirostane-steroidal saponins from the ethanol extract of the roots of *Bletilla*
*striata*. Fitoterapia.

[21] Sun A, Pang S, Wang G (2016). Separation of chemical constituents from *Bletilla*
*striata* and their antitumor activities. Chin. J Pharm..

[22] Qian C (2015). Antibacterial biphenanthrenes from the fibrous roots of *Bletilla*
*striata*. J. Nat. Prod..

[23] Tu YB (2016). Anticholinesterases and antioxidant alkamides from Piper nigrum fruits. Nat. Prod. Res..

[24] Li Q (2017). Cholinesterase, β-amyloid aggregation inhibitory and antioxidant capacities of Chinese medicinal plants. Ind. Crops Prod..

[25] Wu CH, Tu YB, Li ZY, Li YF (2019). Highly selective carbamate-based butyrylcholinesterase Inhibitors derived from a naturally occurring pyranoisoflavone. Bioorg. Chem..

[26] Kasimu, R., et al. *Extract**Product**Useful**in**Medicinal**Composition**and**Preparation**of**Drug**for**Treating**Alzheimer**Disease,**Prepared**by**Eluting**Bletilla**striata**Product**by**Petroleum**Ether-Acetone**Mixed**Solvent,**and**Drying**Product*. CN104906370-A.

[27] Tu YB, Huang JW, Li YF (2018). Anticholinesterase, antioxidant, and beta-amyloid aggregation inhibitory constituents from *Cremastra*
*appendiculata*. Med. Chem. Res..

[28] Tóth B, Hohmann J, Vasas A (2018). Phenanthrenes: A promising group of plant secondary metabolites. J. Nat. Prod..

[29] Kang YY (2019). Two new stilbenoids from *Bletilla*
*striata*. J. Asian Nat. Prod. Res..

[30] Jiang Y, Gao H (2019). Pharmacophore-based drug design for the identification of novel butyrylcholinesterase inhibitors against Alzheimer's disease. Phytomedicine.

[31] Sharma P, Srivastava P, Seth A (2019). Comprehensive review of mechanisms of pathogenesis involved in Alzheimer's disease and potential therapeutic strategies. Prog. Neurobiol..

[32] Pajouhesh H, Lenz GR (2005). Medicinal chemical properties of successful central nervous system drugs. NeuroRx.

[33] Xiao SJ (2016). Three new 1-(p-hydroxybenzyl) phenanthrenes from *Bletilla*
*striata*. J. Asian Nat. Prod. Res..

[34] Bai L, Masukawa N, Yamaki M, Takagi S (1998). Four stilbenoids from *Pleione*
*bulbocodioides*. Phytochemistry.

[35] Yamaki M, Bai L, Inoue K, Takagi S (1990). Benzylphenanthrenes from *Bletilla*
*striata*. Phytochemistry.

[36] Lin CW (2016). Chemical constituents of the rhizomes of *Bletilla*
*formosana* and their potential anti-inflammatory activity. J. Nat. Prod..

[37] Wang X, Cui B, Wang C, Li S (2014). Chemical constituents from *Pleione*
*yunnanensis*. China J. Chin. Mater. Med..

[38] Matsuda H, Morikawa T, Xie H, Yoshikawa M (2004). Antiallergic phenanthrenes and stilbenes from the tubers of *Gymnadenia*
*conopsea*. Planta Med..

[39] Majumder P, Ghosal S (1993). Two stilbenoids from the orchid *Arundina*
*bambusifolia*. Phytochemistry.

[40] Yang M (2015). Studies on the chemical constituents of *Monomeria*
*barbata*. J. Yunnan Univ. (Natural Sciences Edition).

[41] Rueda DC (2014). Identification of dihydrostilbenes in *Pholidota*
*chinensis* as a new scaffold for GABAA receptor modulators. Bioorg. Med. Chem..

[42] Yamaki M, Bai L, Inoue K, Takagi S (1989). Biphenanthrenes from *Bletilla*
*striata*. Phytochemistry.

[43] Bai L, Kato T, Inoue K, Yamaki M, Takagi S (1991). Blestrianol A, B and C, biphenanthrenes from *Bletilla*
*striata*. Nat. Med..

[44] Bai L, Masukawa N, Yamaki M, Takagi S (1998). A polyphenol and two bibenzyls from *Pleione*
*bulbocodioides*. Phytochemistry.

[45] Liu X, Yuan Q, Guo Y (2009). Two new stilbenoids from *Pleione*
*bulbocodioides*. J. Asian Nat. Prod. Res..

[46] Liu L, Li J, Zeng K, Jiang Y, Tu P (2016). Five new biphenanthrenes from *Cremastra*
*appendiculata*. Molecules.

[47] Bai L, Yamaki M, Inoue K, Takago S (1990). Blestrin A and B, bis(dihydrophenanthrene) ethers from *Bletilla*
*striata*. Phytochemistry.

[48] Ellman GL, Courtney KD, Andres V, Featherstone RM (1961). A new and rapid colorimetric determination of acetylcholinesterase activity. Biochem. Pharmacol..

[49] Lu T (2021). Discovery, biological evaluation and molecular dynamic simulations of butyrylcholinesterase inhibitors through structure-based pharmacophore virtual screening. Future Med. Chem..

