# Supplementary material for: Biological evaluation, molecular modeling and dynamics simulation of phenanthrenes isolated from Bletilla striata as butyrylcholinesterase inhibitors
Source: Sci Rep. 2022 Aug 11;12:13649. doi: 10.1038/s41598-022-17912-7 (PMC9372051; doi:10.1038/s41598-022-17912-7)
Supplement: Supplementary file 1 — Supplementary Information. [file 41598_2022_17912_MOESM1_ESM.docx]

**Biological evaluation, molecular modeling and dynamics simulation of Phenanthrenes isolated from Bletilla striata as butyrylcholinesterase inhibitors**

Yi Liu ^1^, Yanbei Tu ^2^, Yunyao Kang ^1^, Chao Zhu ^1^, Chuanhai Wu ^1^, Gang Chen ^3,4^, Zerong Liu ^3,4^,

Yanfang Li ^1,^*

^1^ School of Chemical Engineering, Sichuan University, Chengdu, 610065, China

^2^ School of pharmacy, Jiangsu University, Zhenjiang, 212012, China

^3^ Central Nervous System Drug Key Laboratory of Sichuan Province, Luzhou, 646106, China

^4^ Sichuan Credit Pharmaceutical CO., Ltd. Luzhou, 646106, China

* Corresponding author：E-mail address: lyf471@vip.163.com.

**Figure S1.** Isolation scheme of anticholinesterases bio-guide fractionation of B. striata tubers.

**Figure S2.** The three times parallel RMSD of 10(A) and 12(B) with BChE.

**Figure S3.** Percentage inhibition of BChE induced by compounds 6(A), 8(B), 10(C), 12(D), 14(E) in comparison to Galantamine(F).

**Table S1** Inhibition activity of extract/fractions of *B. striata* against two cholinesterases.

**Table S2.** HPLC method.

The representative ^1^H NMR, ^13^C NMR, 2D-NMR, HR ESI-MS spectrum and HPLC chromatogram of compounds **1-22.**

***

***

**Figure S1.** Isolation scheme of anticholinesterases bio-guide fractionation of *B. striata* tubers.


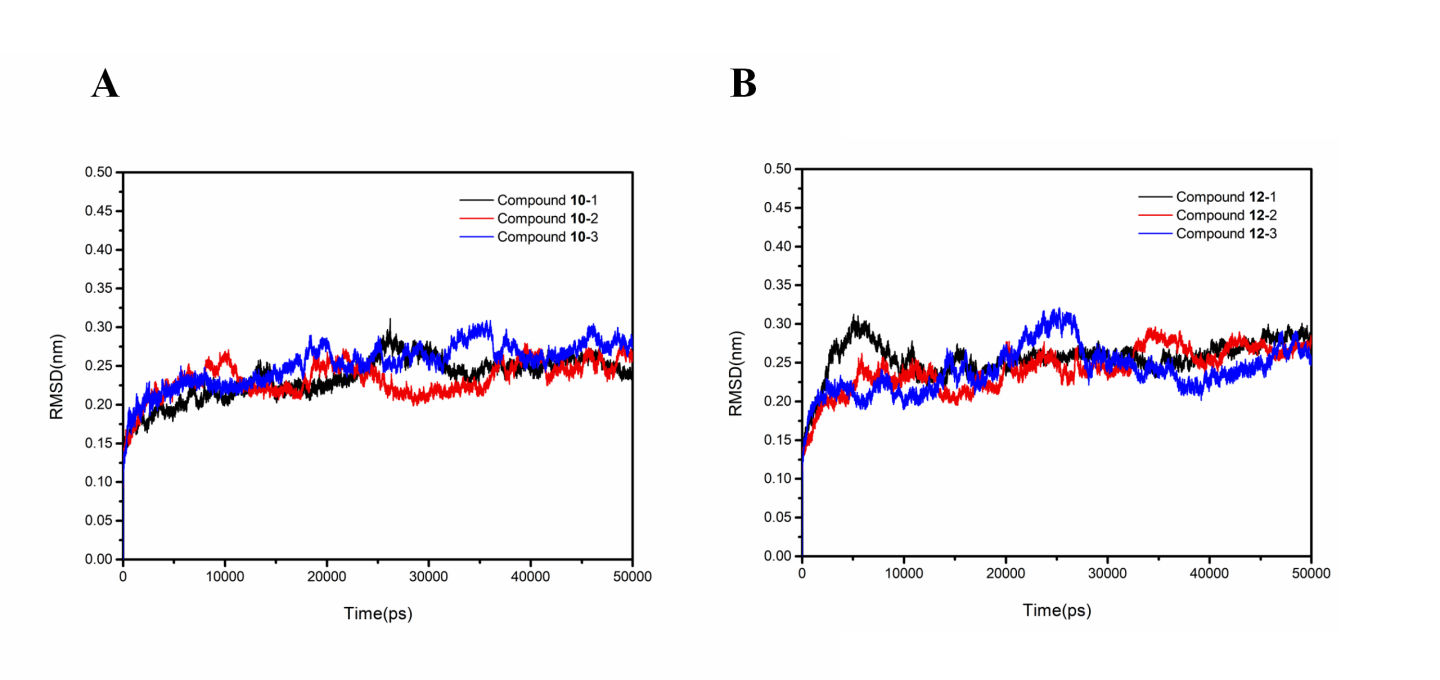


**Figure S2.** The three times parallel RMSD of **10**(A) and **12**(B) with BChE.


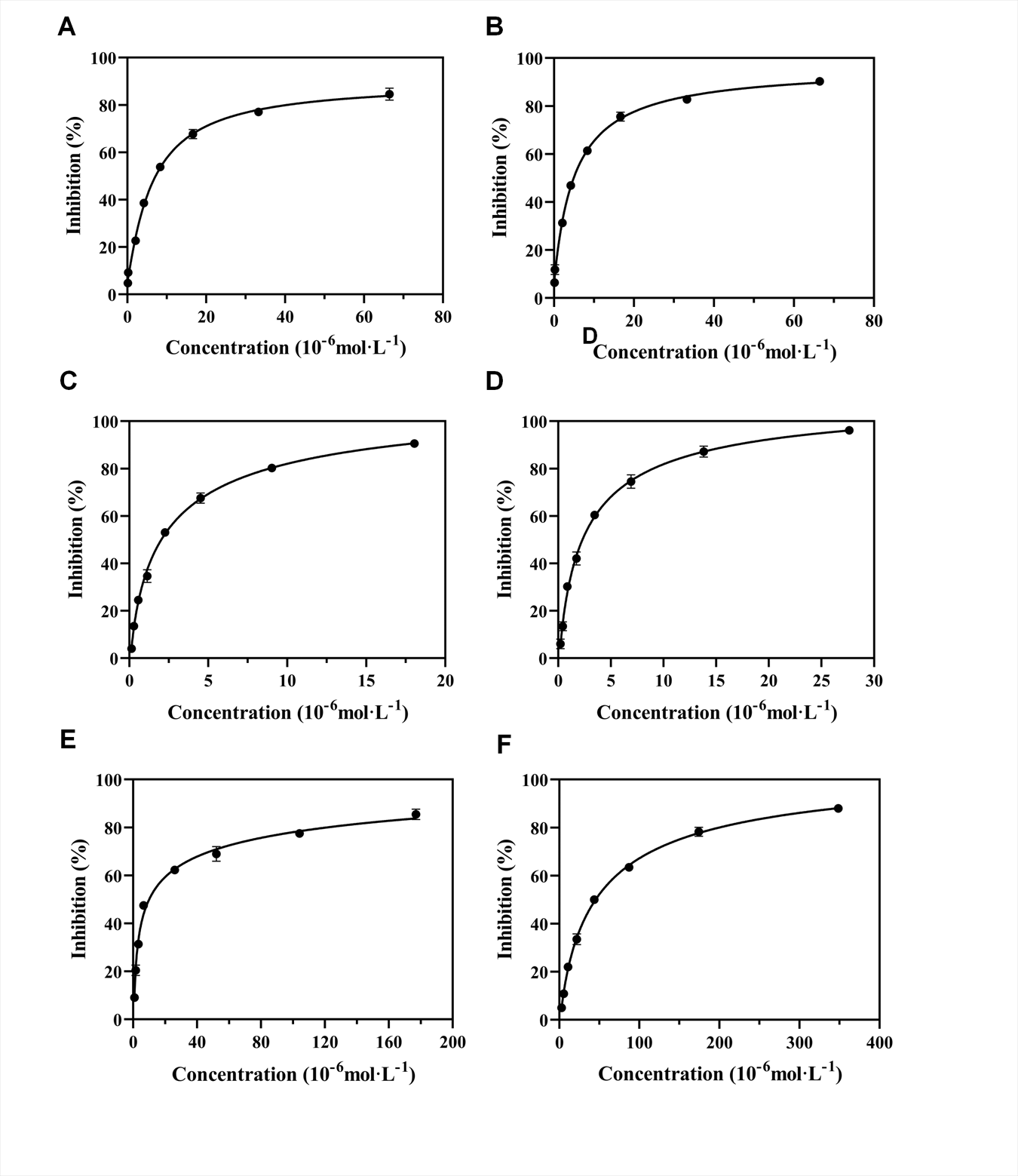


**Figure S3.** Percentage inhibition of BChE induced by compounds **6**(A), **8**(B), **10**(C), **12**(D), **14**(E) in comparison to galantamine (F).

Table S1 Inhibition activity of extract/fractions of *B. striata* against two cholinesterases.

| extracts/fractions | IC_50_ (*μ*g/mL) | | Inhibition against BChE(%) | |
| --- | --- | --- | --- | --- |
|  | BChE | AChE | 50 μg/mL | 25 μg/mL |
| *95% EtOH* | 8.6 ± 0.2 | 296.4 ± 22.5 |  |  |
| *PE* | 187.9 ±11.5 | 355.6 ± 15.2 |  |  |
| *EtOAc* | 2.3 ± 0.1 | 224.0 ± 18.9 |  |  |
| *n-BuOH* | 213.2 ±12.4 | 479.8 ± 25.7 |  |  |
| *Water* | >600 | >600 |  |  |
| *galantamine ^a^* | 13.2 ± 0.8 | 4.3 ± 0.3 |  |  |
| Fr.A |  |  | 20.5 ± 5.1 | 8.9 ± 4.0 |
| Fr.B |  |  | 23.5 ± 9.9 | 10.4 ± 2.2 |
| Fr.C |  |  | 37.2 ± 1.5 | 30.6 ± 4.6 |
| Fr.D |  |  | 53.1 ± 16.1 | 43.4 ± 11.3 |
| Fr.E |  |  | 27.4 ± 10.1 | 20.4 ± 5.7 |
| Fr.F |  |  | 32.0 ± 3.4 | 17.6 ± 1.8 |
| Fr.G |  |  | 39.8 ± 8.2 | 25.6 ± 6.9 |
| Fr.H |  |  | 60.1 ± 2.5 | 43.3 ± 4.8 |
| Fr.I |  |  | 67.2 ± 2.4 | 47.2 ± 5.3 |
| Fr.J |  |  | 87.1 ± 3.6 | 76.8 ± 3.9 |
| Fr.K |  |  | 96.5 ± 2.2 | 94.4 ± 2.7 |
| Fr.J1 |  |  |  | 3.0 ± 1.1 |
| Fr.J2 |  |  |  | 3.3 ± 2.7 |
| Fr.J3 |  |  |  | 2.0 ± 0.3 |
| Fr.J4 |  |  |  | 2.5 ± 1.5 |
| Fr.J5 |  |  |  | 2.0 ± 0.6 |
| Fr.J6 |  |  |  | 17.6 ± 3.1 |
| Fr.J7 |  |  |  | 55.7 ± 2.8 |
| Fr.J8 |  |  |  | 58.5 ± 0.7 |
| Fr.J9 |  |  |  | 80.7 ± 1.8 |
| Fr.J10 |  |  |  | 84.5 ± 0.7 |
| Fr.J11 |  |  |  | 85.0 ± 3.2 |
| Fr.K1 |  |  |  | 1.0 ± 0.8 |
| Fr.K2 |  |  |  | 4.5 ± 3.9 |
| Fr.K3 |  |  |  | 16.5 ± 6.5 |
| Fr.K4 |  |  |  | 91.0 ± 2.6 |
| Fr.K5 |  |  |  | 90.3 ± 0.2 |
| Fr.K6 |  |  |  | 95.1 ± 2.9 |
| Fr.K7 |  |  |  | 81.2 ± 5.5 |
| Fr.K8 |  |  |  | 29.5 ± 6.0 |
| Fr.K9 |  |  |  | 14.6 ± 4.6 |

^a^ positive control

**Table S2.** HPLC method**.**

The purities of compounds were determined by the HPLC methods shown in following table.

The peak aera was determined according UV (280 nm) or ELSD detector.

| Equipment | Alltech Modells 201 with a ELSD 6000 detector | | |
| --- | --- | --- | --- |
|  |  |  |  |
| Column | Nacalai tesque COSMOSIL Packed column AR-Ⅱ (4.6 ID × 250 mm) | | |
|  |  |  |  |
| Method | CH_3_OH/ 0.1% formic acid, from 40% (v/v) of CH_3_OH gradient in the beginning to 100%(v/v) of CH_3_OH gradient in 40 minutes | | |
|  |  |  |  |
|  | flow rate: 1.0 mL /min | | |
|  |  |  |  |
|  |  |  |  |
| Results | Compounds | Retention time (min) | Relative purity (%) |
|  | **1** | 14.04 | 96.03 |
|  | **2** | 16.93 | 97.98 |
|  | **3** | 18.25 | 96.84 |
|  | **4** | 15.15 | 97.99 |
|  | **5** | 18.09 | 98.92 |
|  | **6** | 20.00 | 98.84 |
|  | **7** | 15.92 | 99.14 |
|  | **8** | 8.90 | 99.22 |
|  | **9** | 10.60 | 99.12 |
|  | **10** | 10.18 | 99.43 |
|  | **11** | 11.63 | 98.87 |
|  | **12** | 11.64 | 97.55 |
|  | **13** | 11.90 | 98.91 |
|  | **14** | 9.45 | 98.18 |
|  | **15** | 24.07 | 96.30 |
|  | **16** | 26.45 | 97.79 |
|  | **17** | 13.32 | 95.69 |
|  | **18** | 17.86 | 92.32 |
|  | **19** | 13.17 | 99.11 |
|  | **20** | 26.82 | 97.93 |
|  | **21** | 13.17 | 99.11 |
|  | **22** | 15.68 | 99.16 |


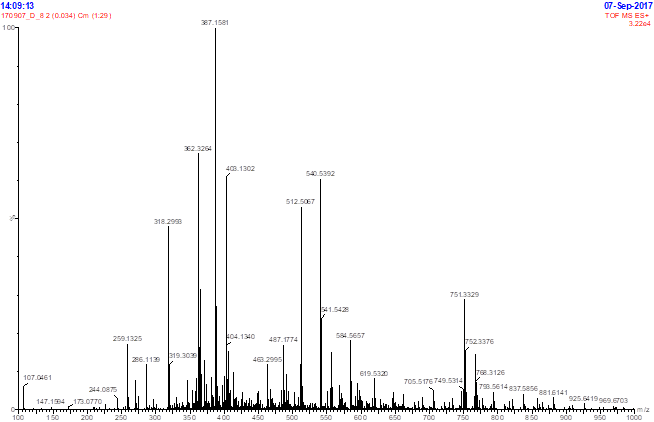


HR-ESI-MS spectrum of compound **1**


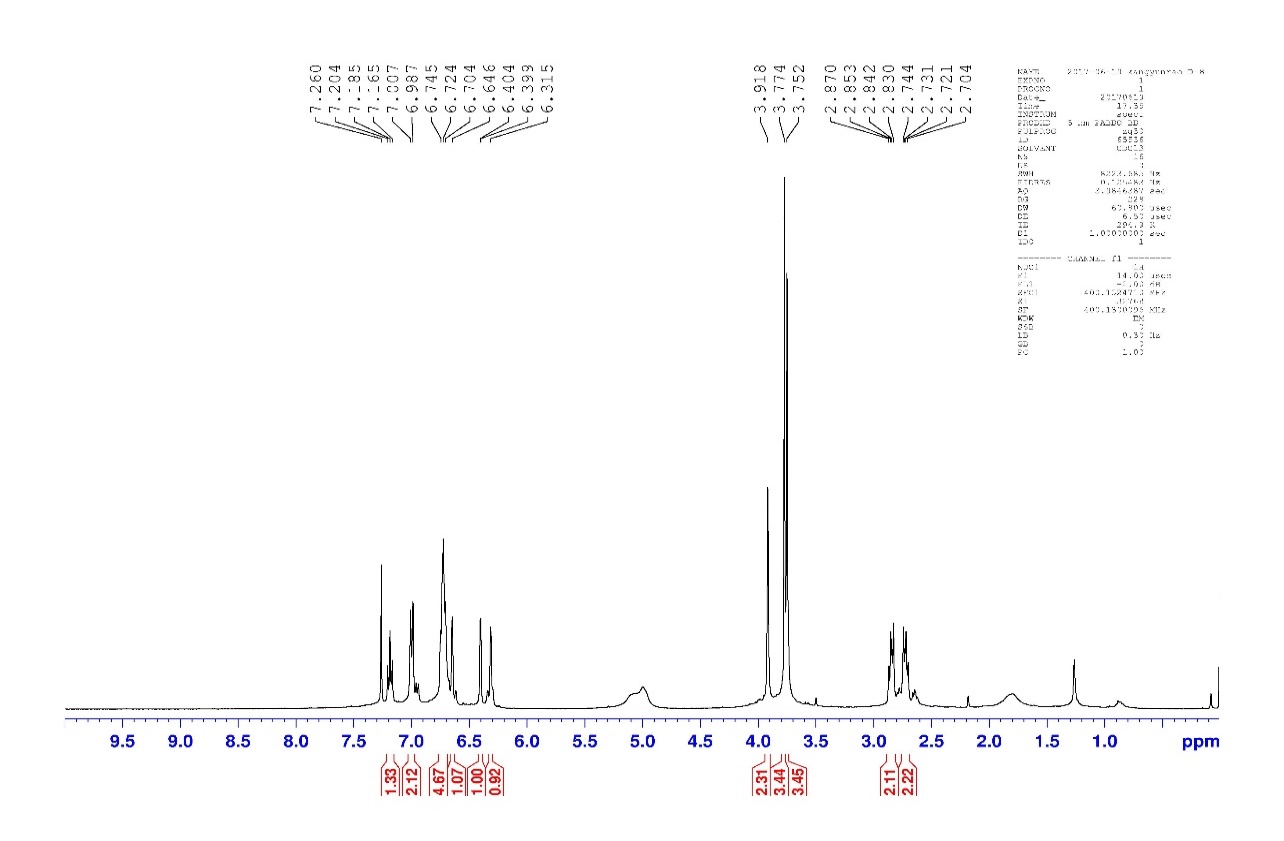


^1^H NMR (400 MHz, CDCl_3_) spectrum of compound **1**

**
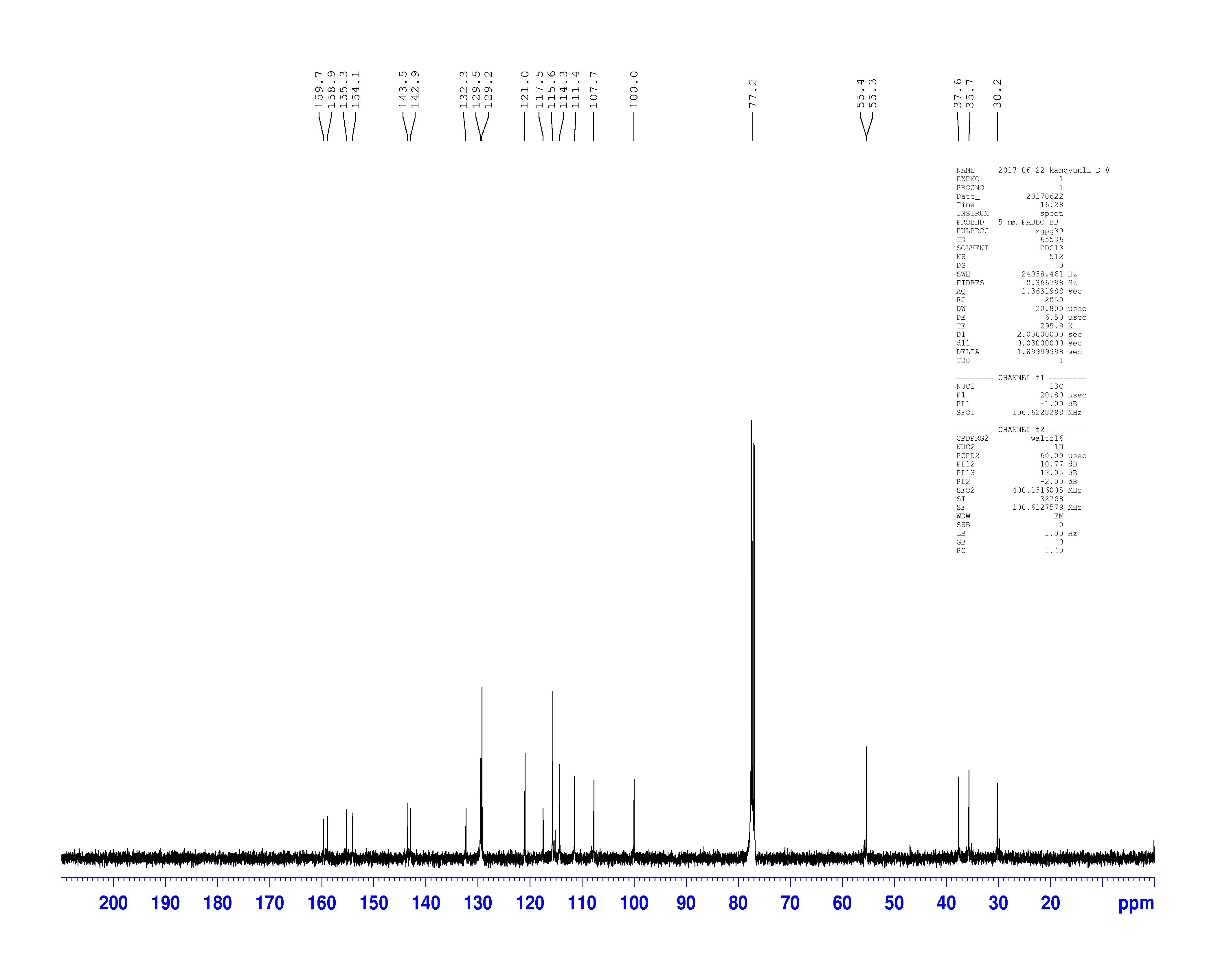
**

^13^C NMR (100 MHz, CDCl_3_) spectrum of compound **1**

**
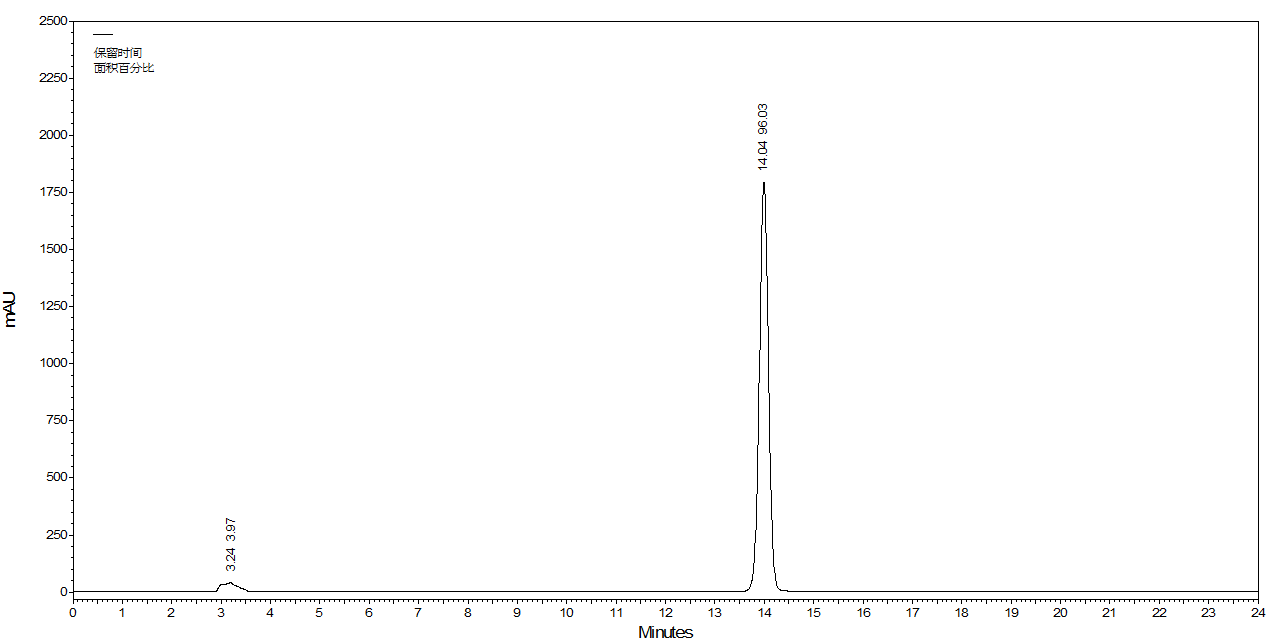
**

HPLC chromatogram of compound **1**

HR-ESI-MS spectrum of compound **2**


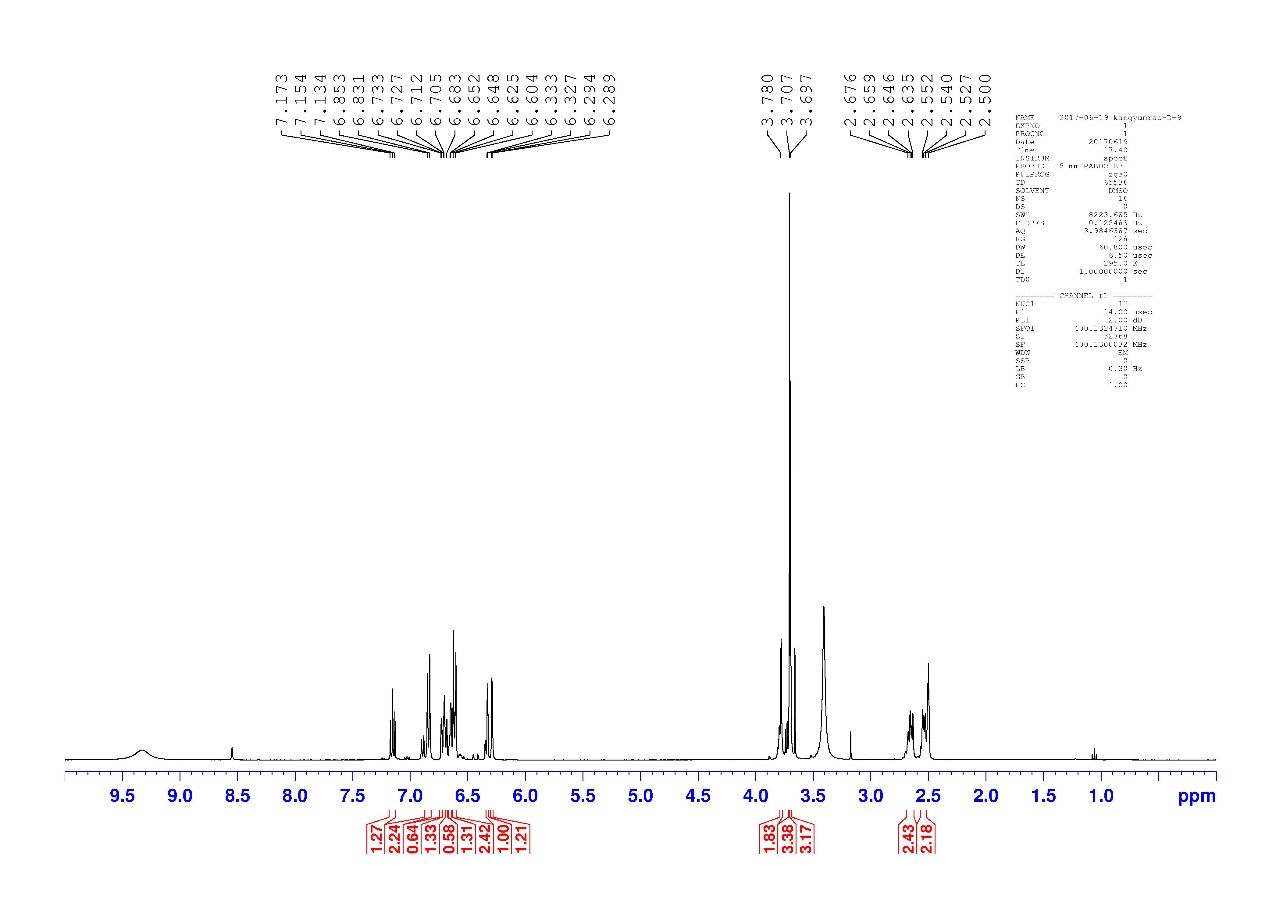


^1^H NMR (400 MHz, DMSO-*d*_6_) spectrum of compound **2**


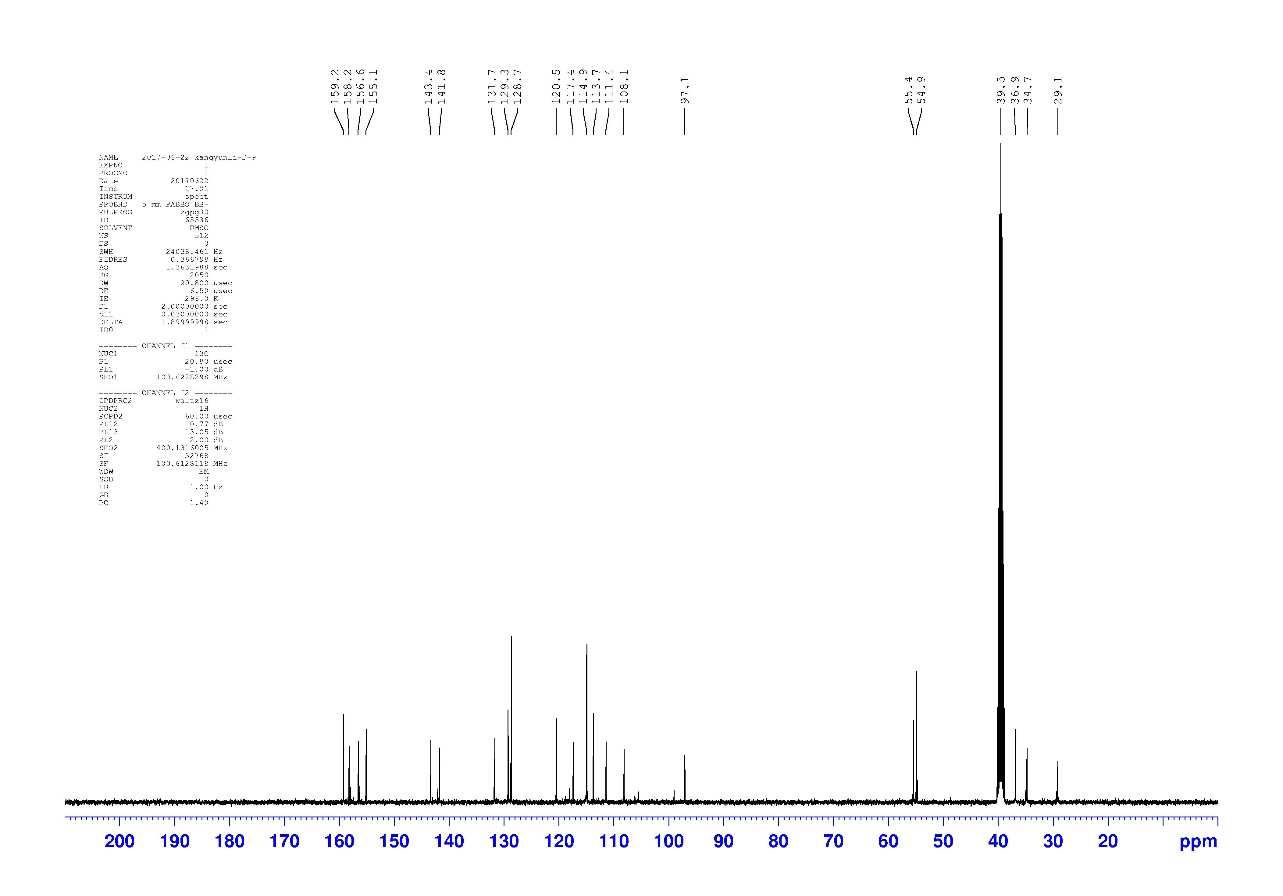


^13^C NMR (100 MHz, DMSO-*d*_6_) spectrum of compound **2**


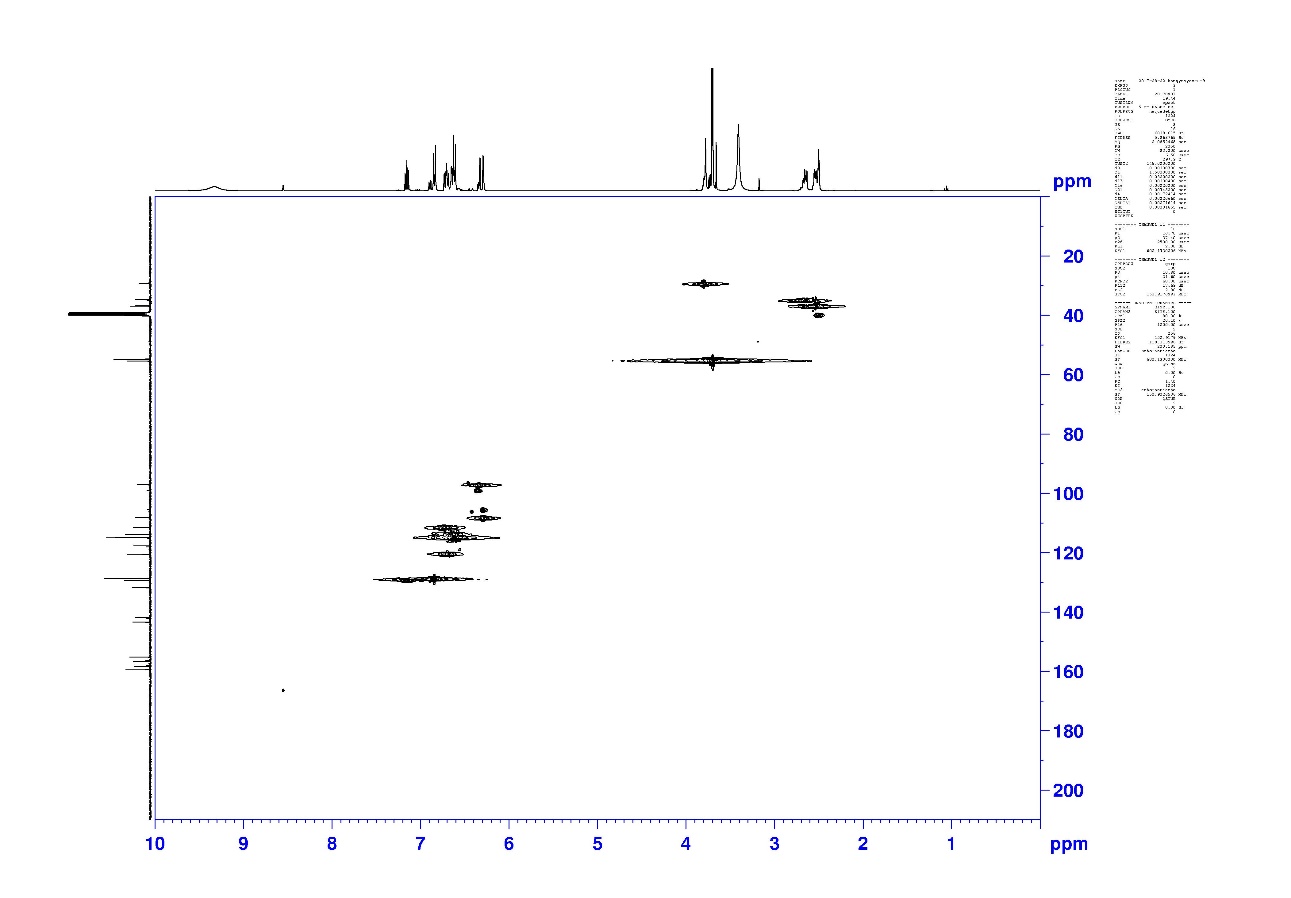


HSQC spectrum of compound **2**

^
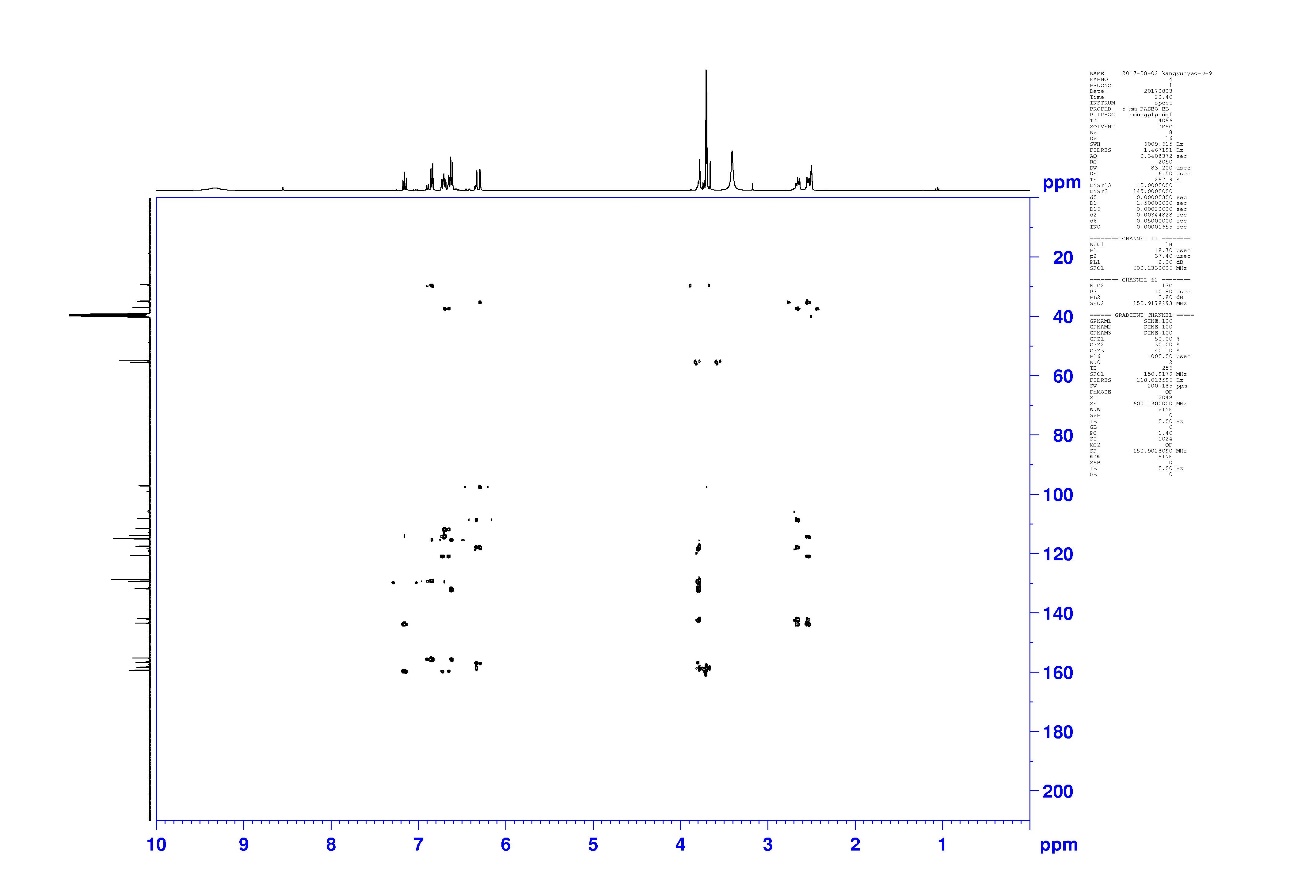
^

HMBC spectrum of compound **2**


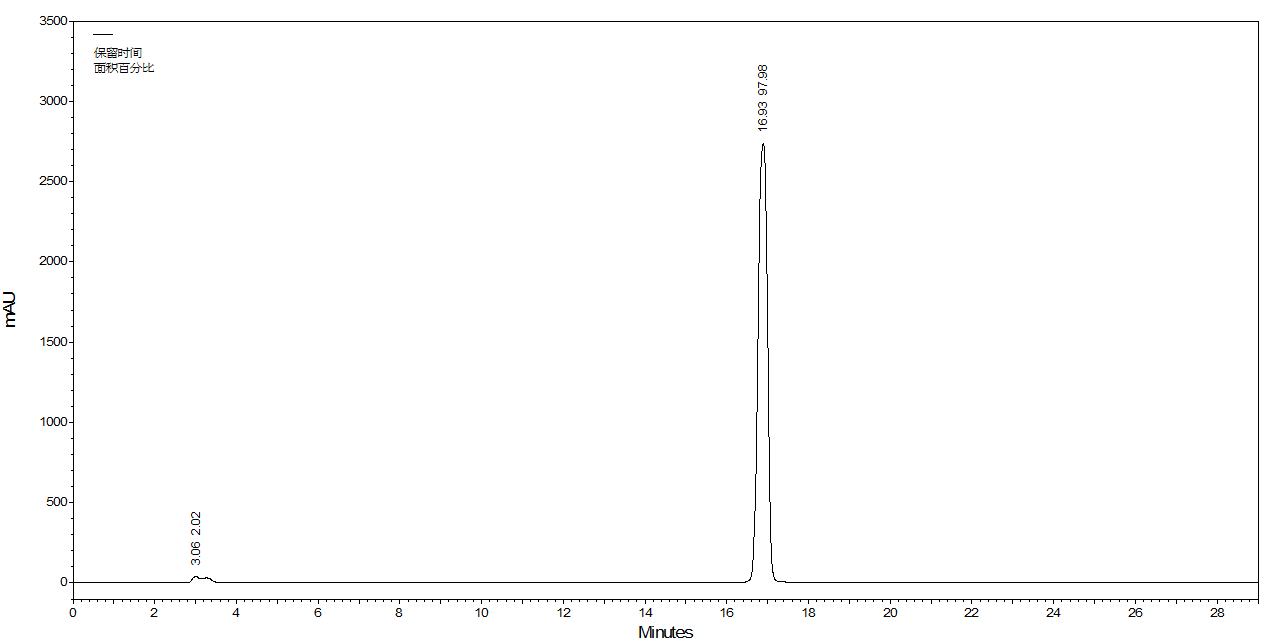


HPLC chromatogram of compound **2**

HR-ESI-MS spectrum of compound **3**


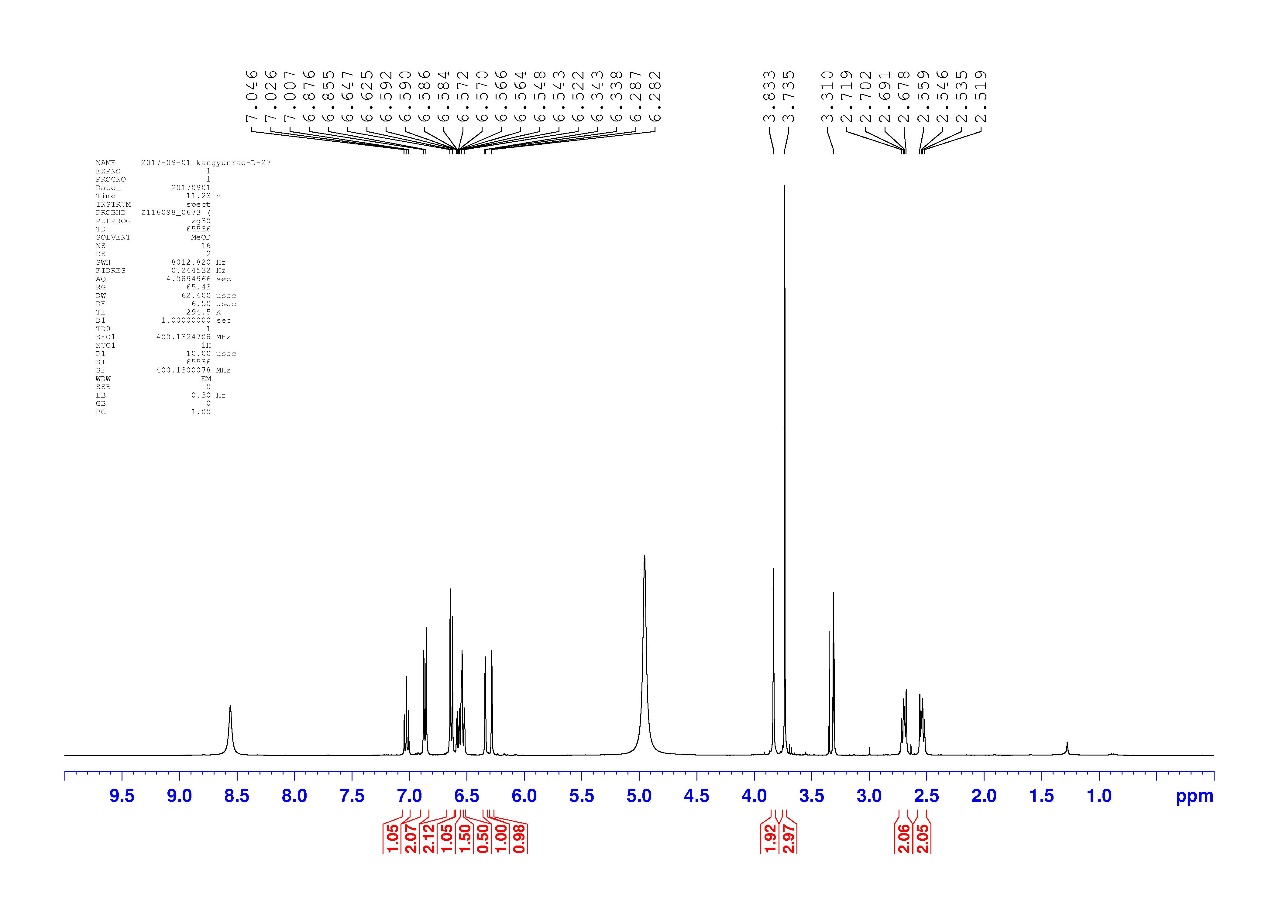


^1^H NMR (400 MHz, CD_3_OD) spectrum of compound **3**


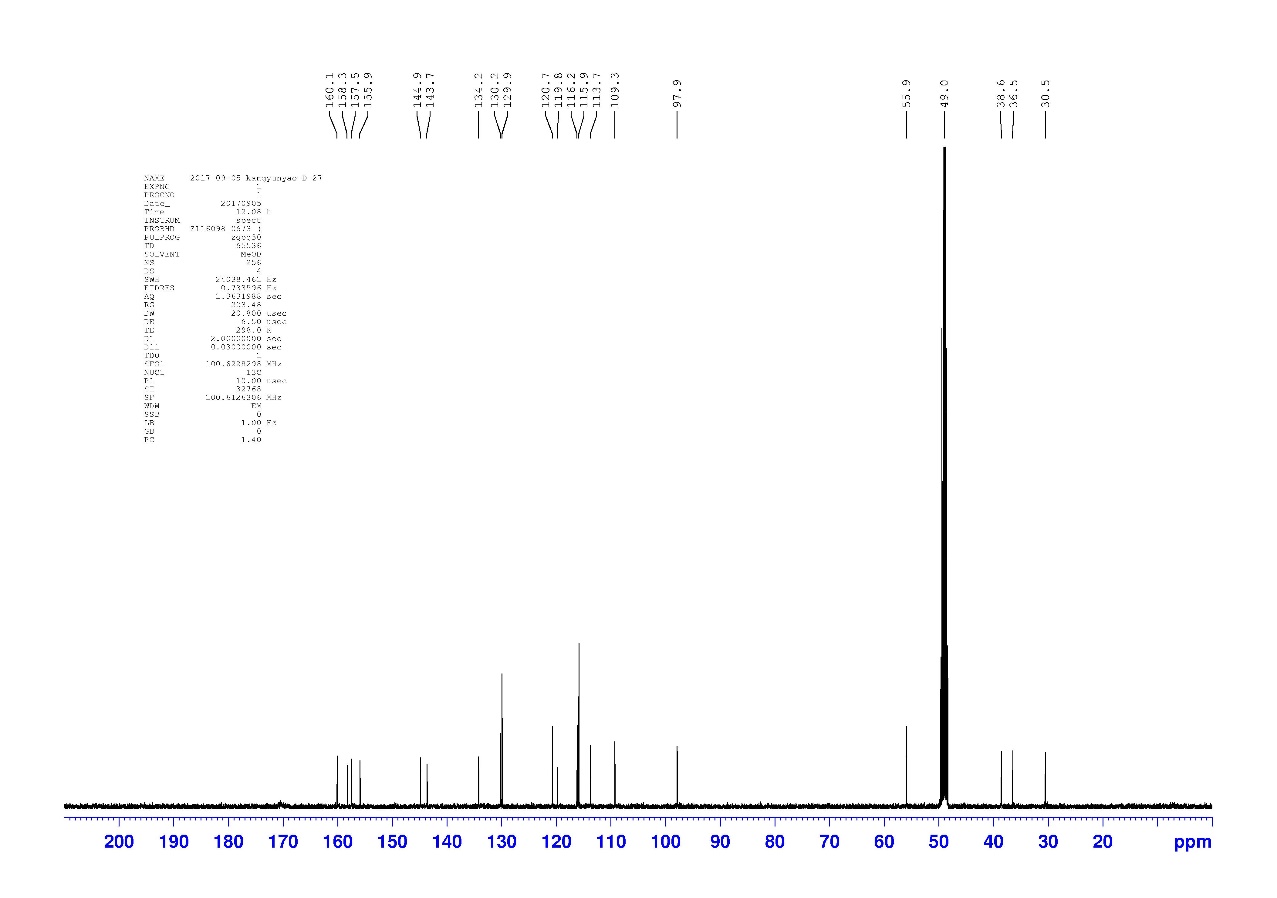


^13^C NMR (100 MHz, CD_3_OD) spectrum of compound **3**


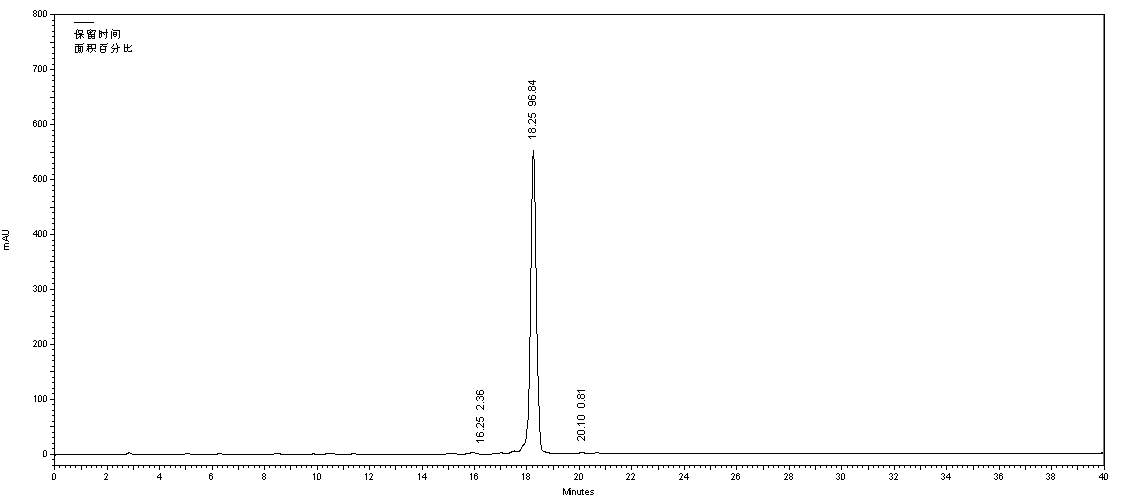


HPLC chromatogram of compound **3**

HR-ESI-MS spectrum of compound **4**


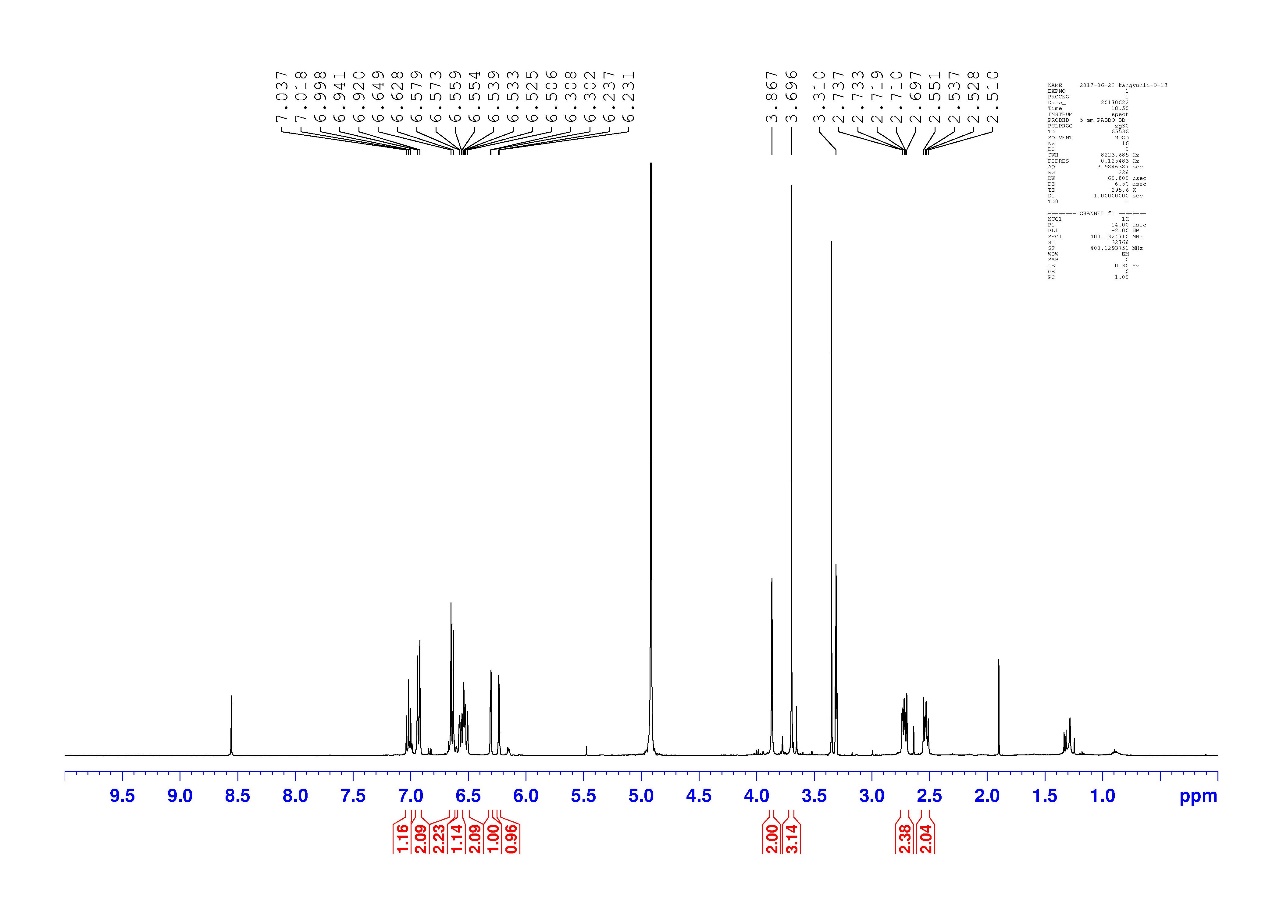


^1^H NMR (400 MHz, CD_3_OD) spectrum of compound **4**


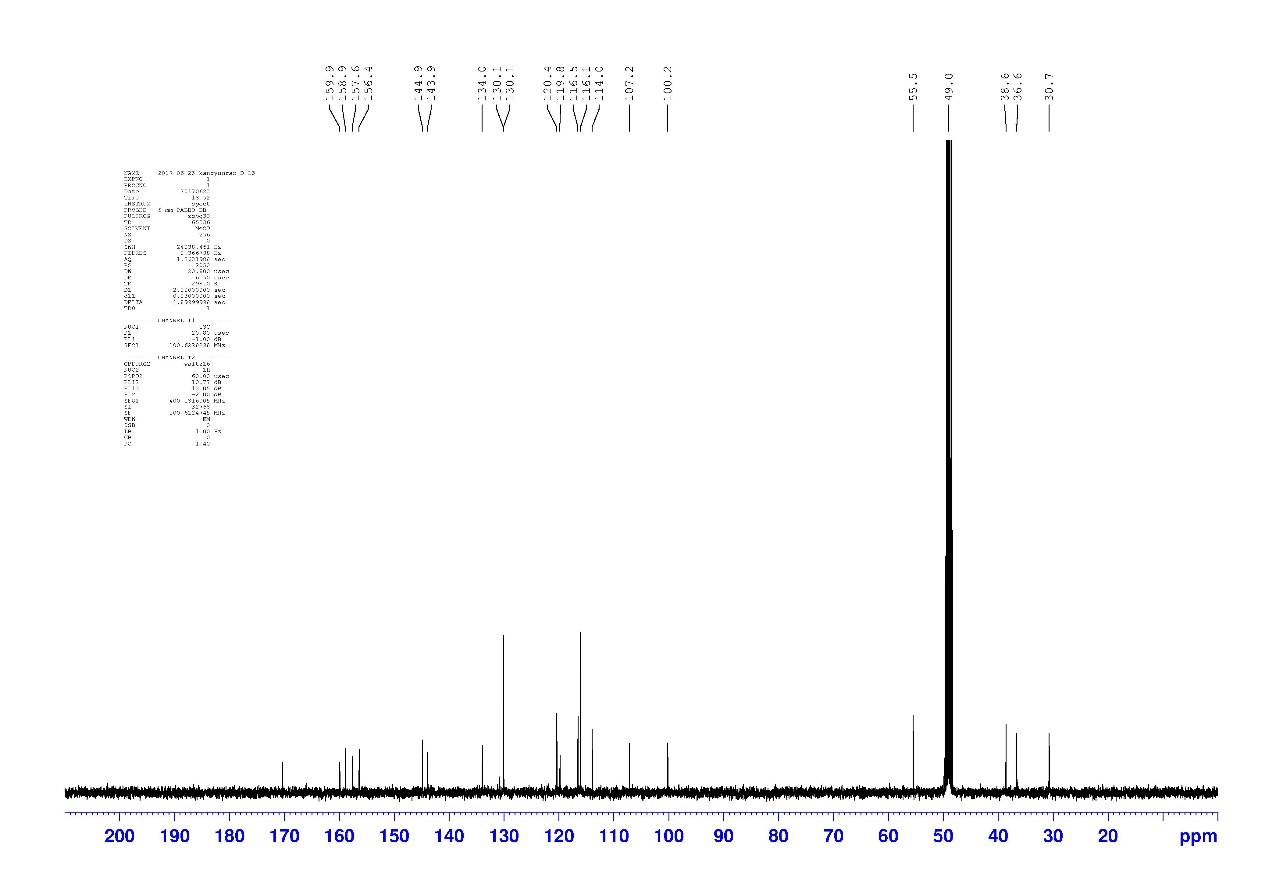


^13^C NMR (100 MHz, CD_3_OD) spectrum of compound **4**

**
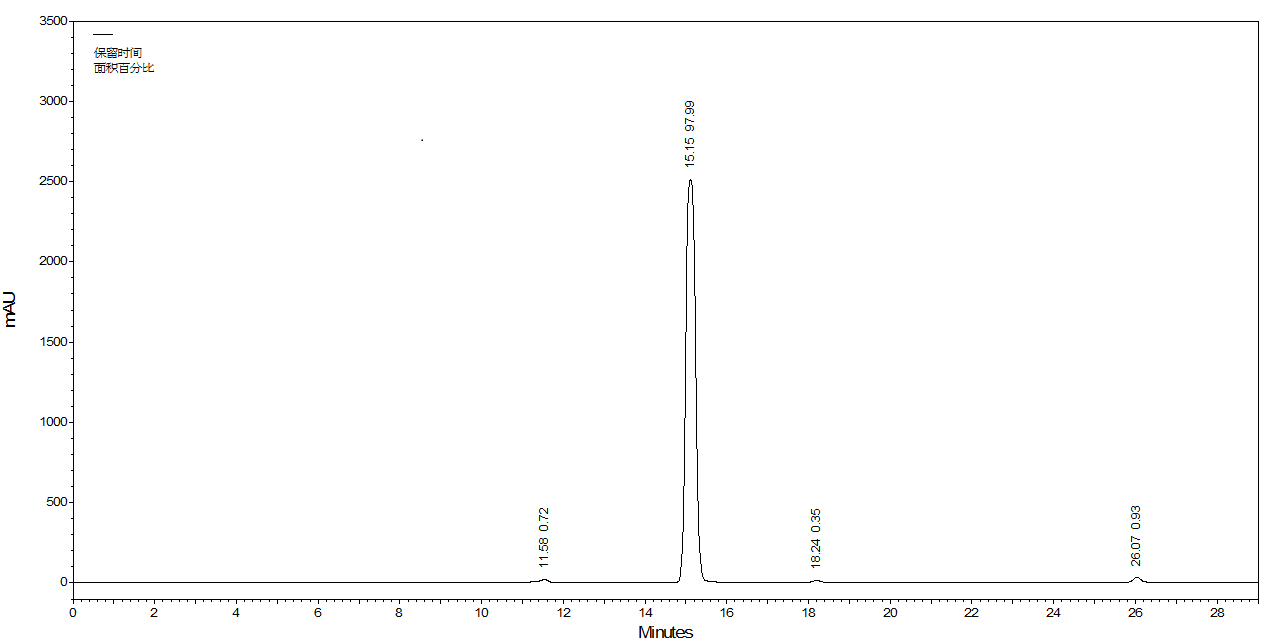
**

HPLC chromatogram of compound **4**


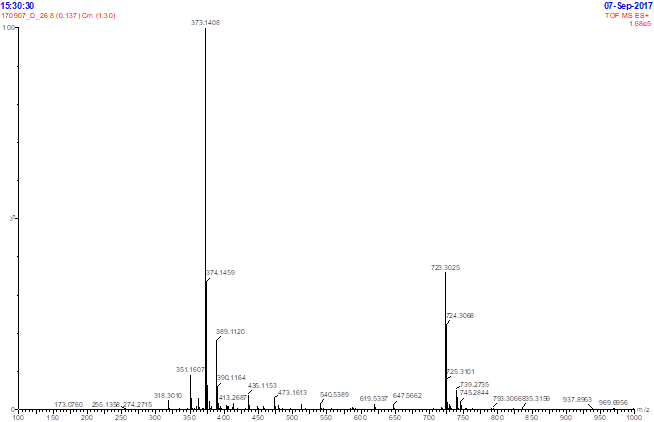


HR-ESI-MS spectrum of compound **5**


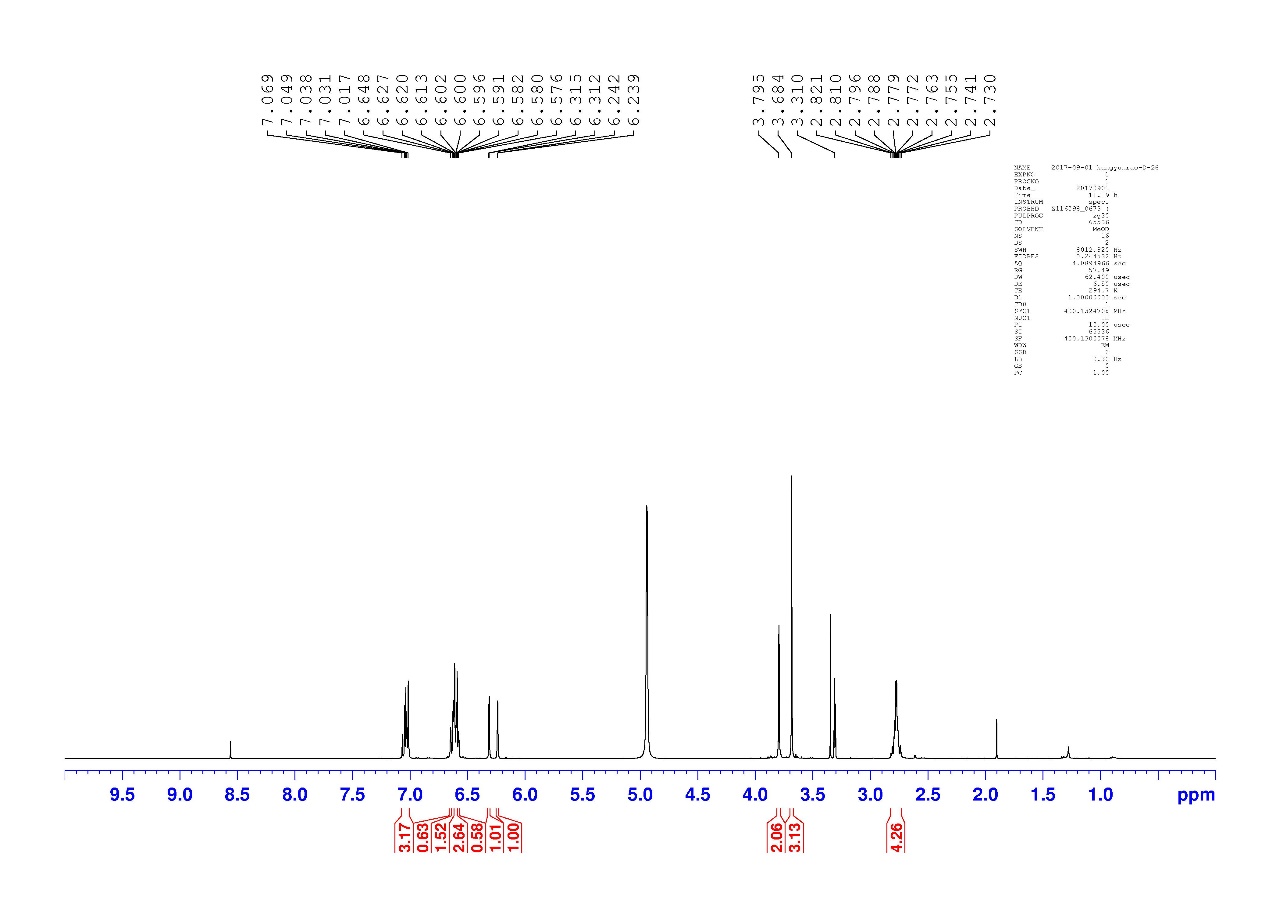


^1^H NMR (400 MHz, CD_3_OD) spectrum of compound **5**


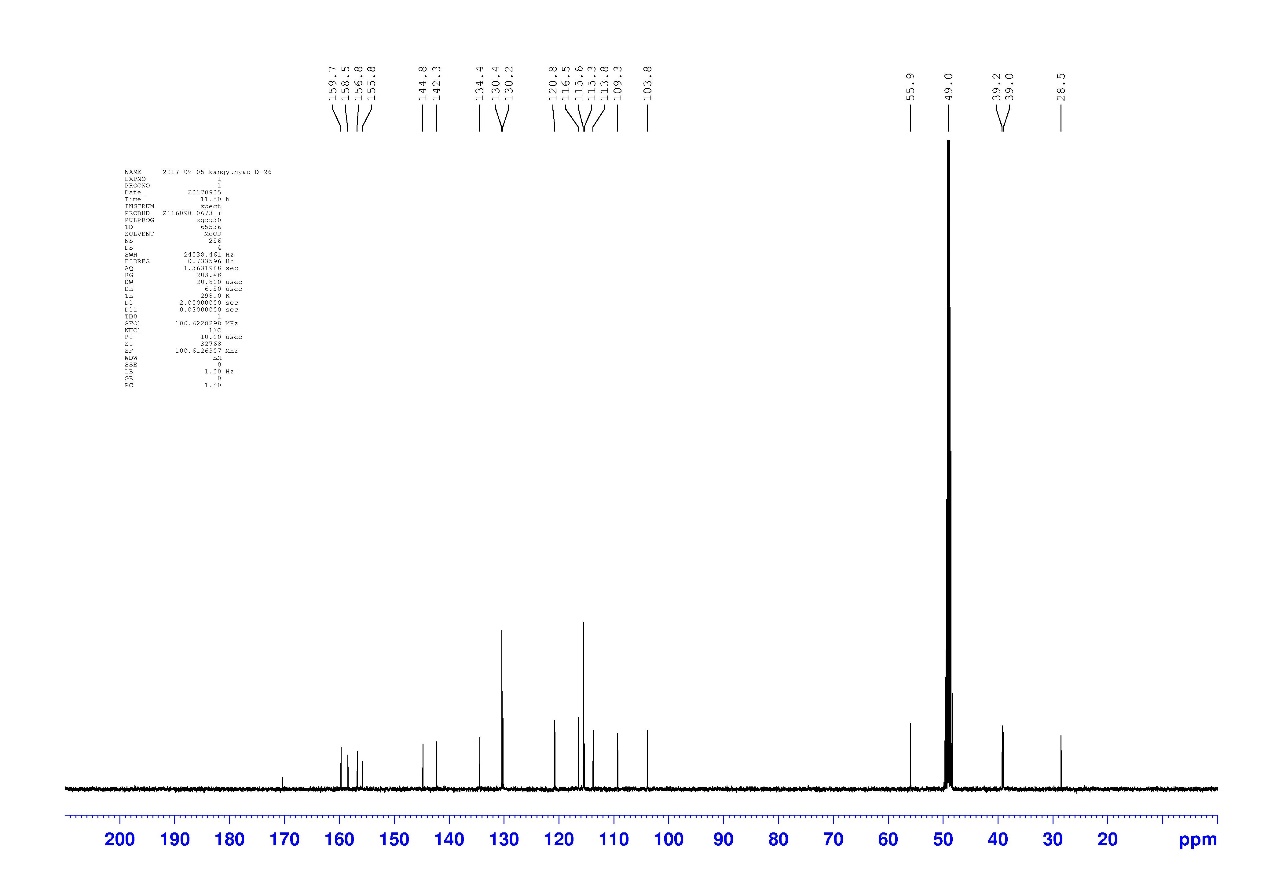


^13^C NMR (100 MHz, CD_3_OD) spectrum of compound **5**


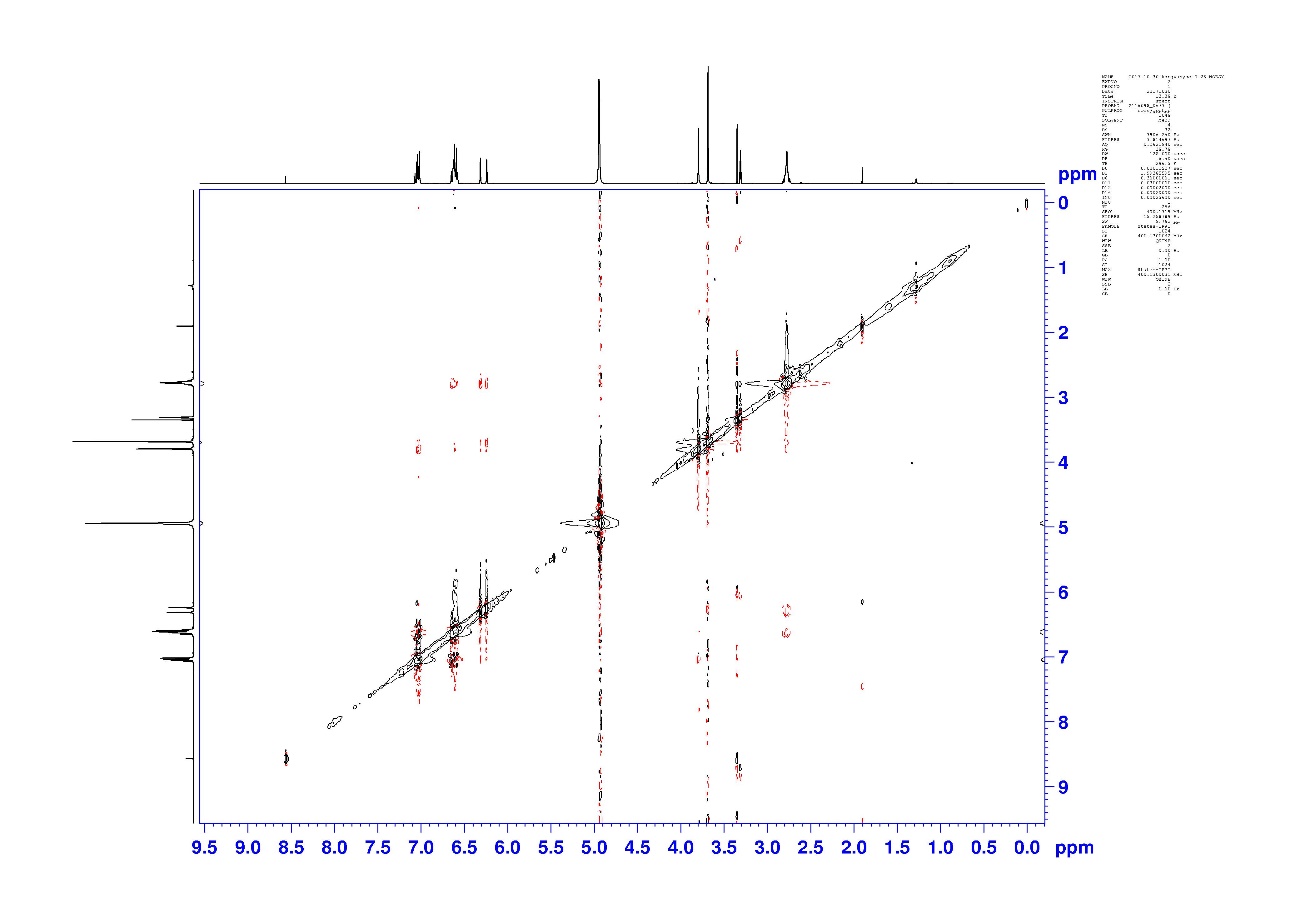


NOESY spectrum of compound 5


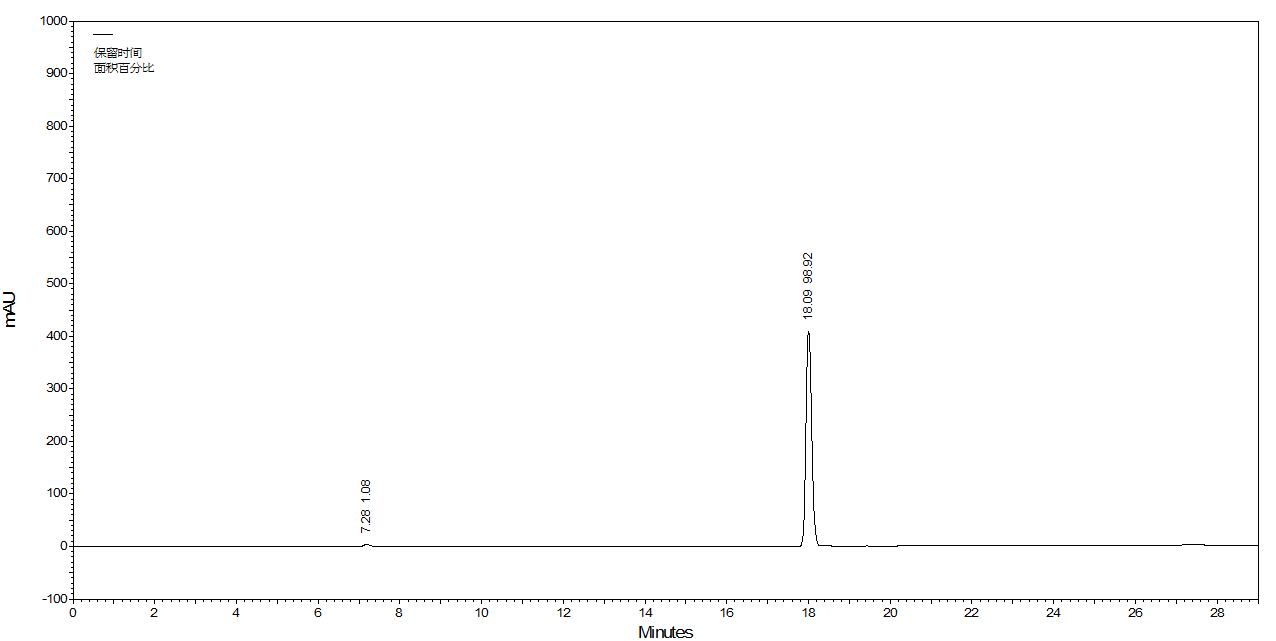


HPLC chromatogram of compound **5**

HR-ESI-MS spectrum of compound **6**


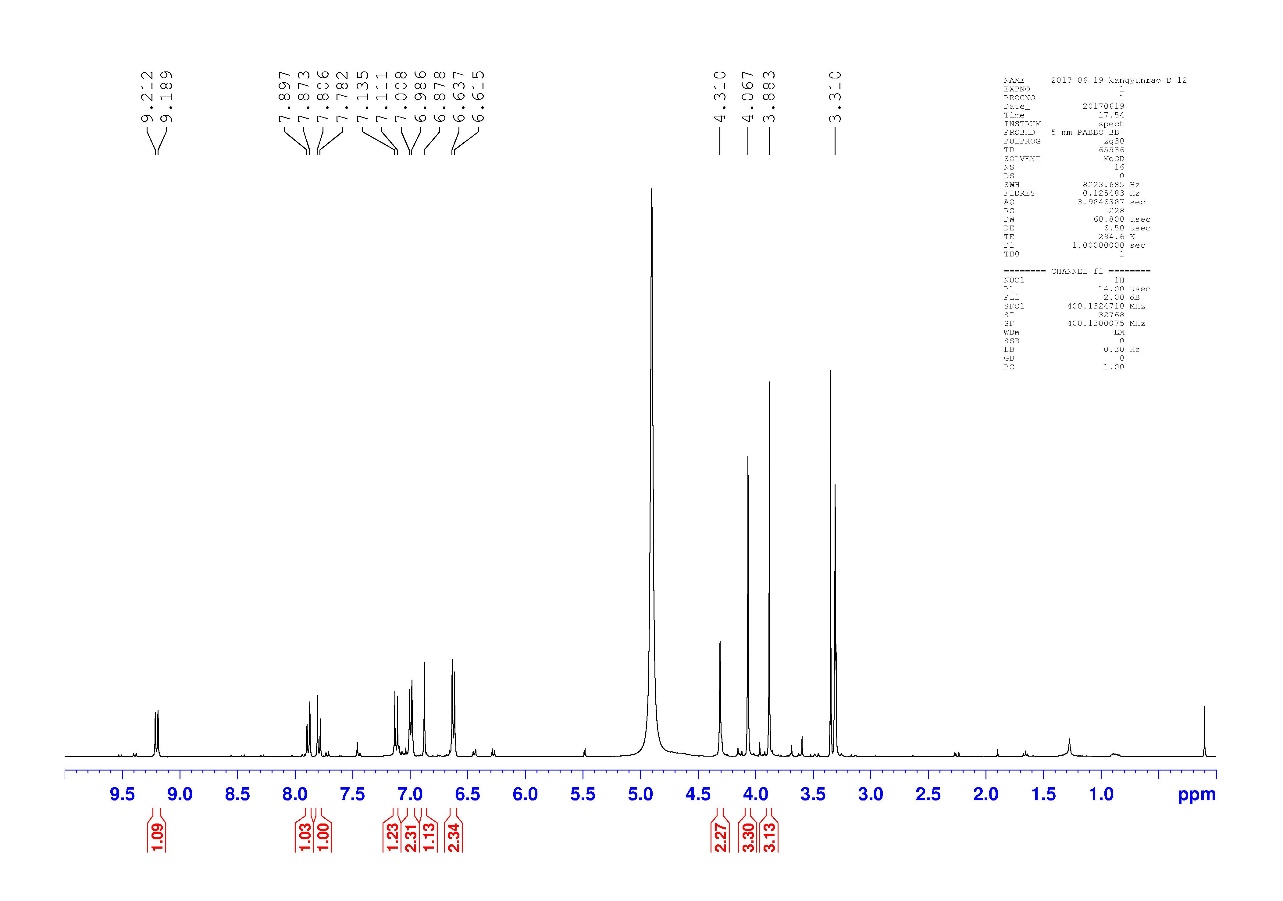


^1^H NMR (400 MHz, CD_3_OD) spectrum of compound **6**


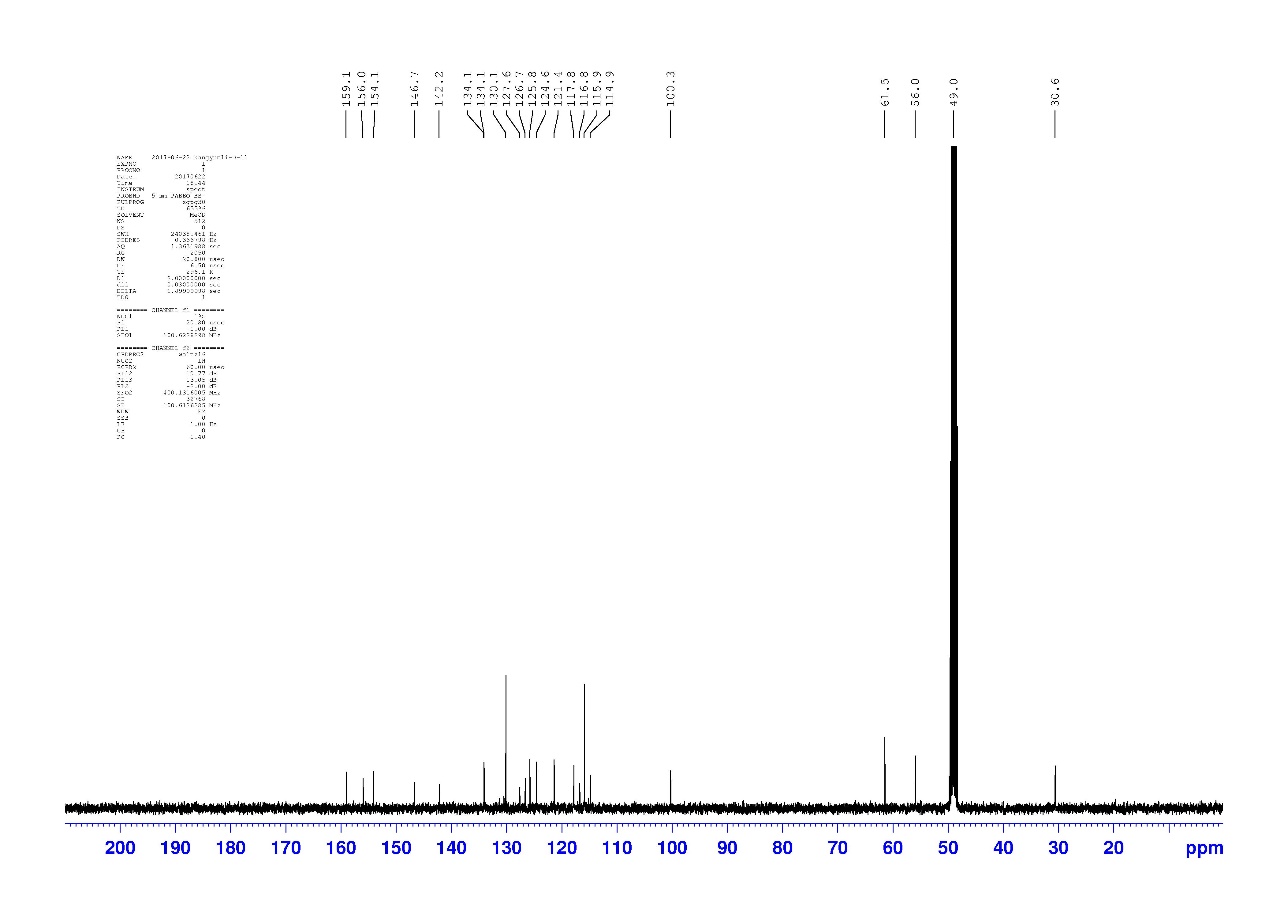


^13^C NMR (100 MHz, CD_3_OD) spectrum of compound **6**

**
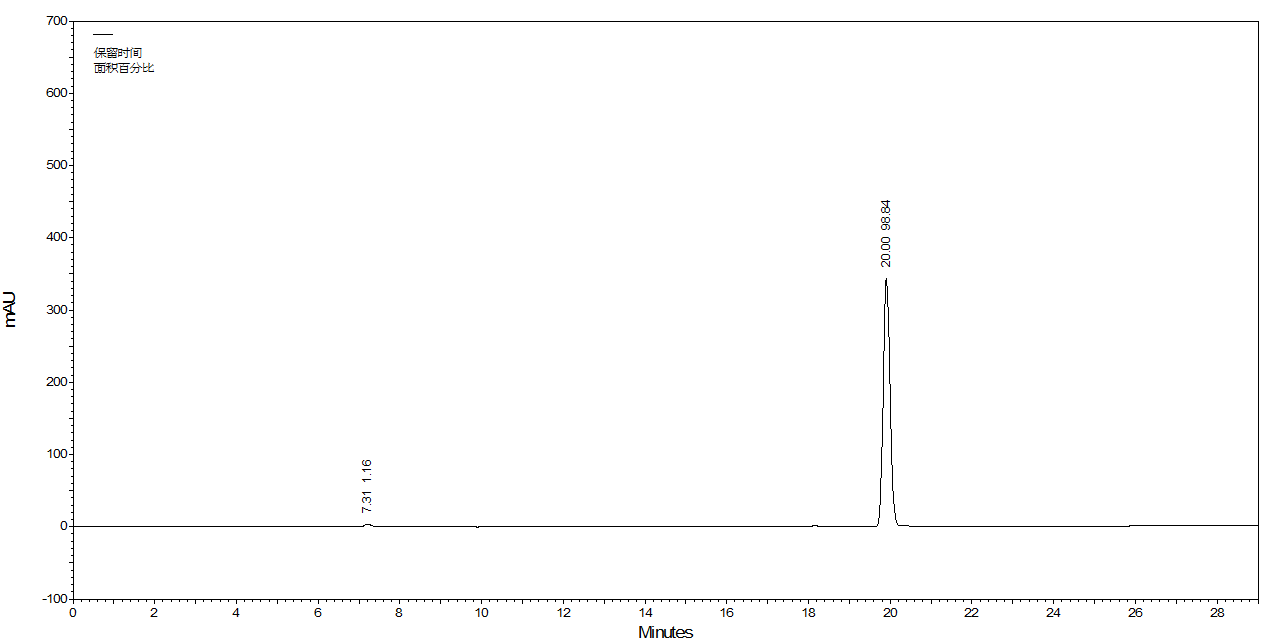
**

HPLC chromatogram of compound **6**


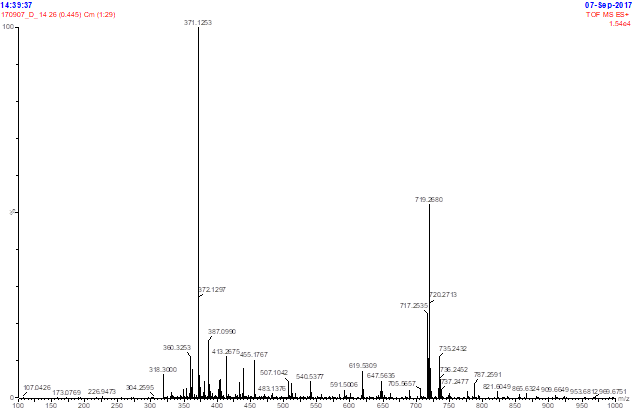


HR-ESI-MS spectrum of compound **7**


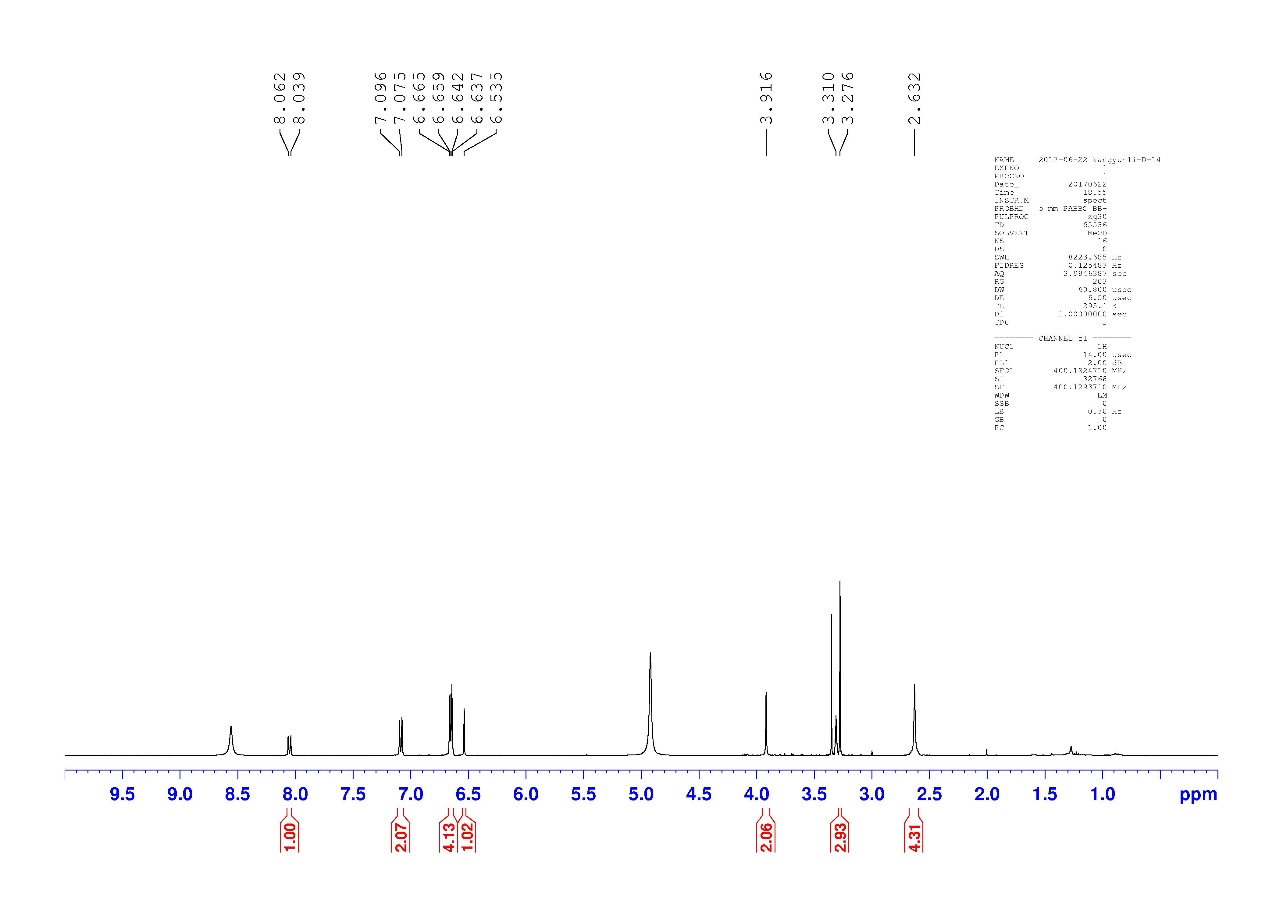


^1^H NMR (400 MHz, CD_3_OD) spectrum of compound **7**


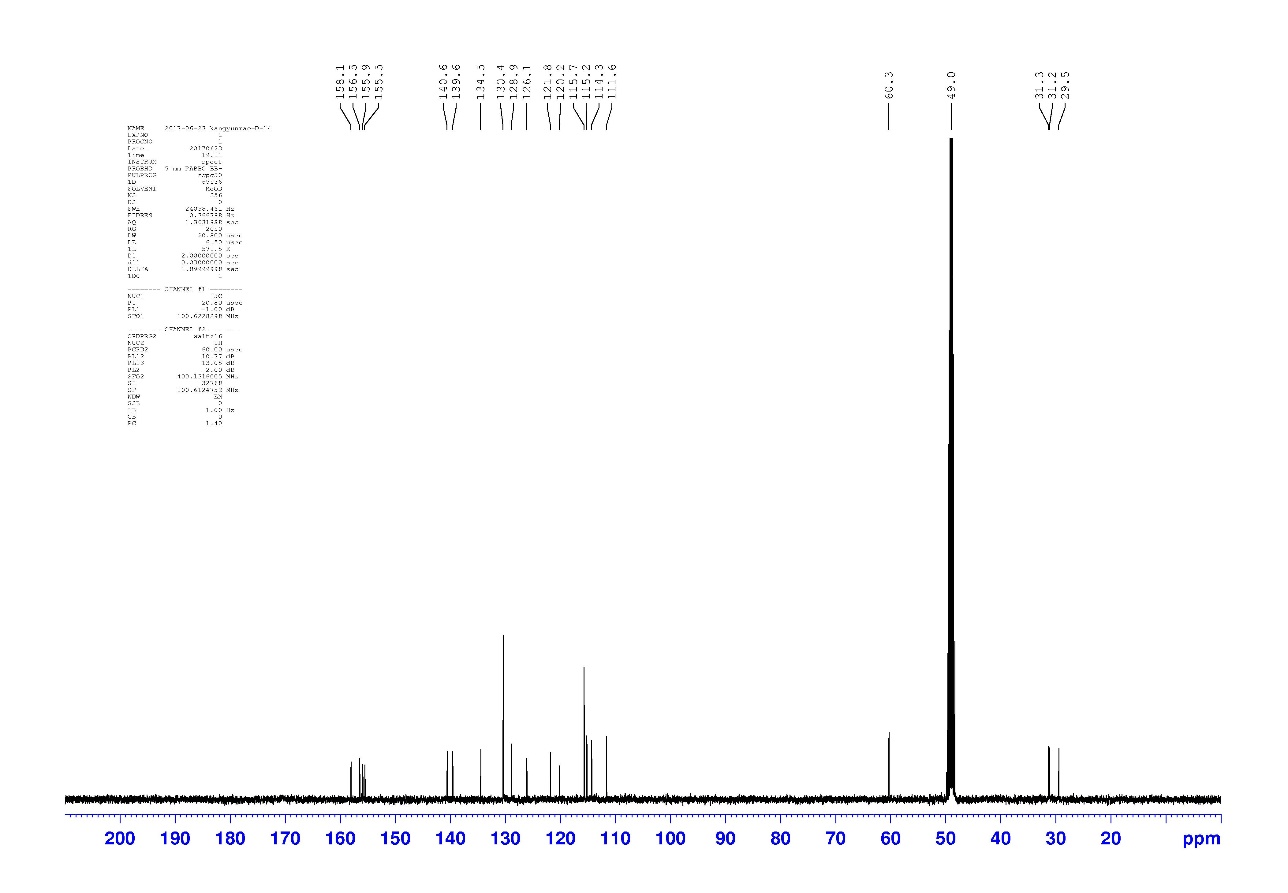


^13^C NMR (100 MHz, CD_3_OD) spectrum of compound **7**


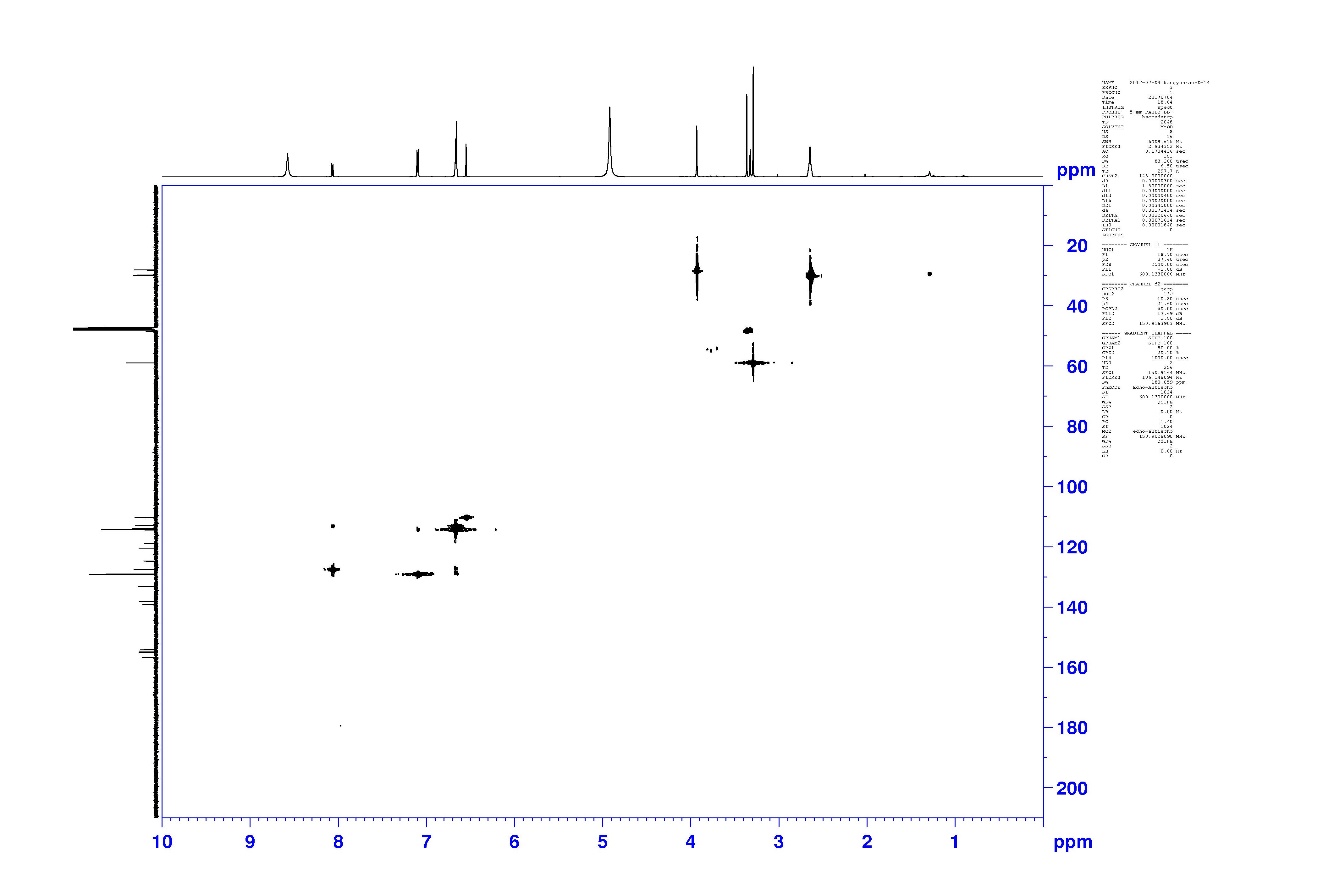


HSQC spectrum of compound **7**


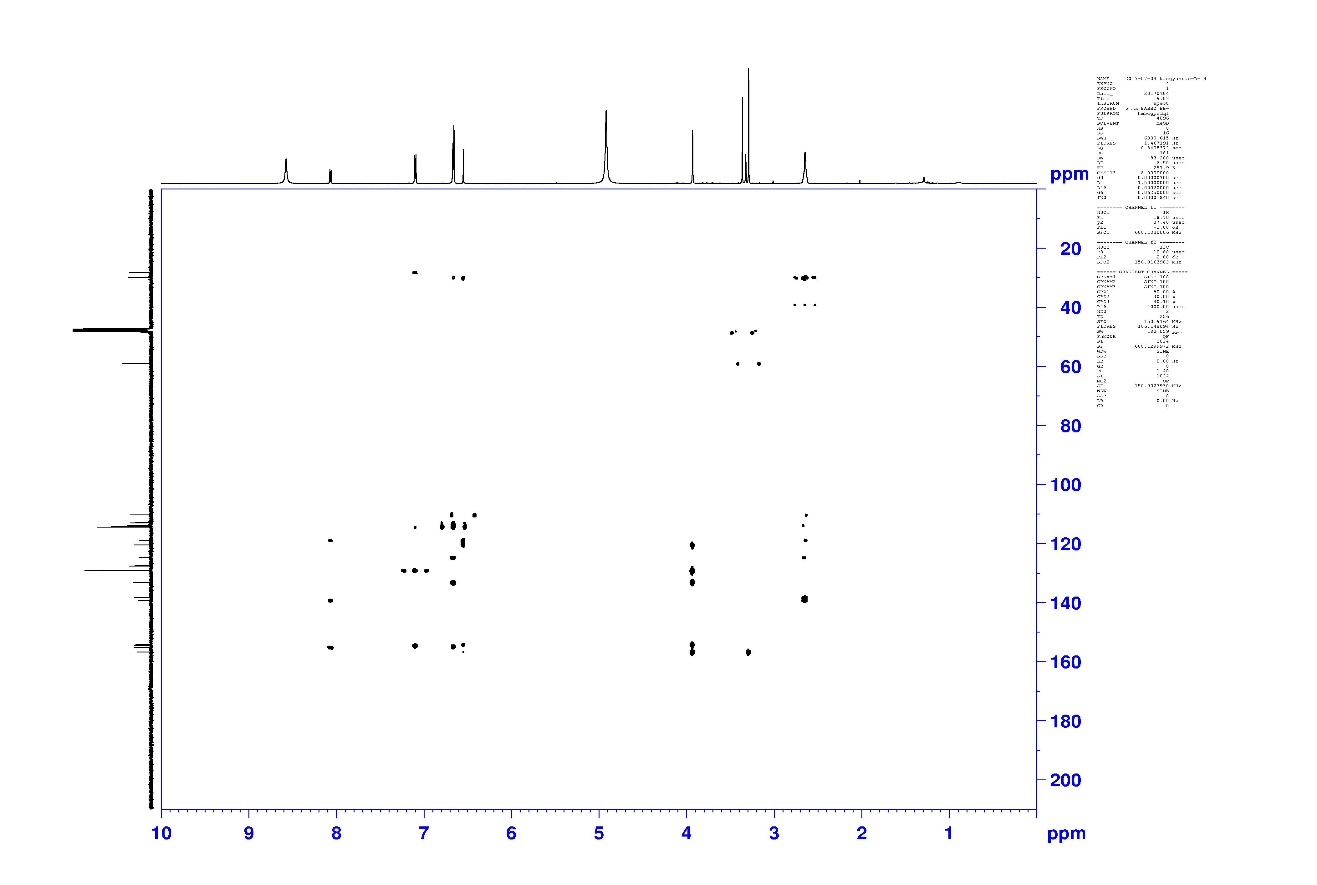


HMBC spectrum of compound **7**

**
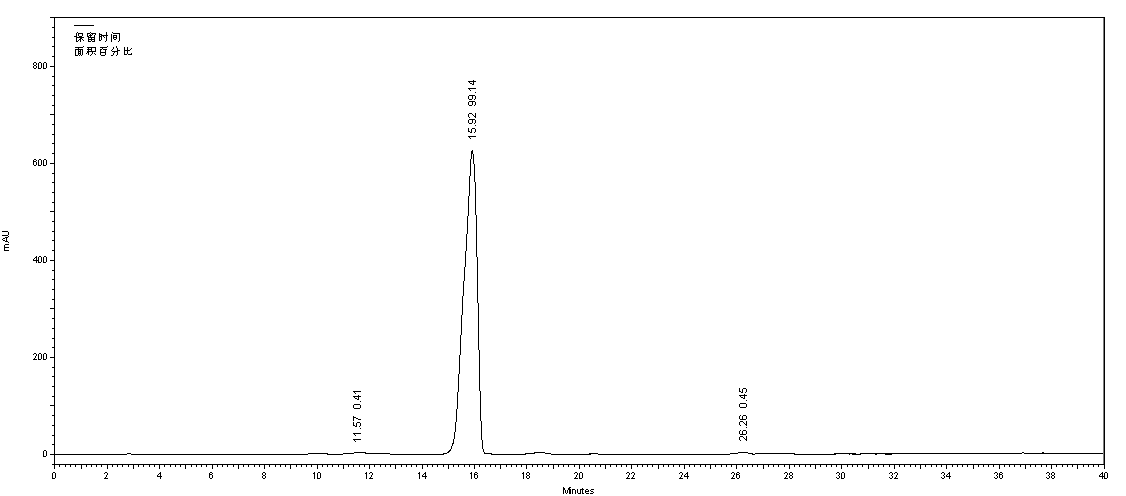
**

HPLC chromatogram of compound **7**

HR-ESI-MS spectrum of compound **8**


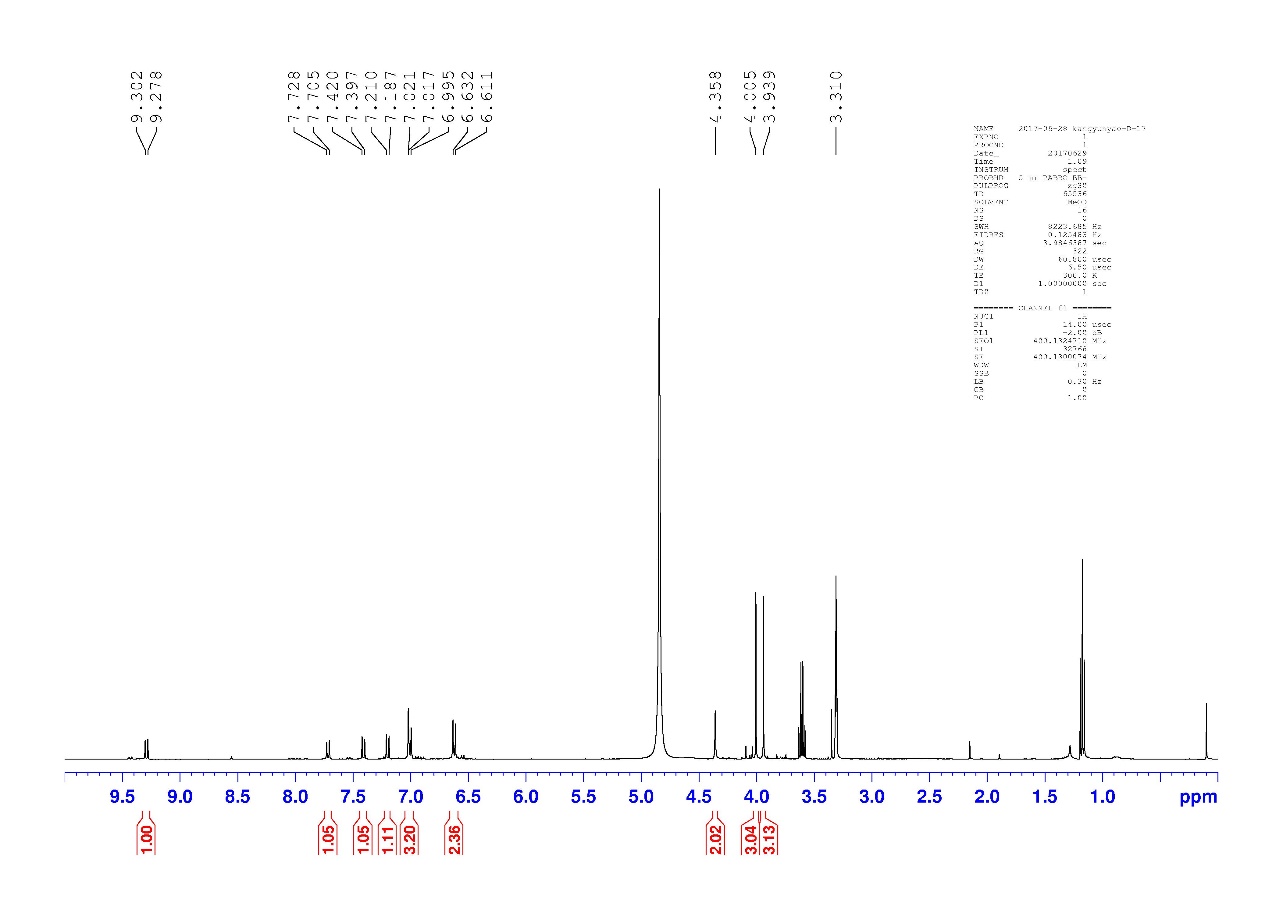


^1^H NMR (400 MHz, CD_3_OD) spectrum of compound **8**


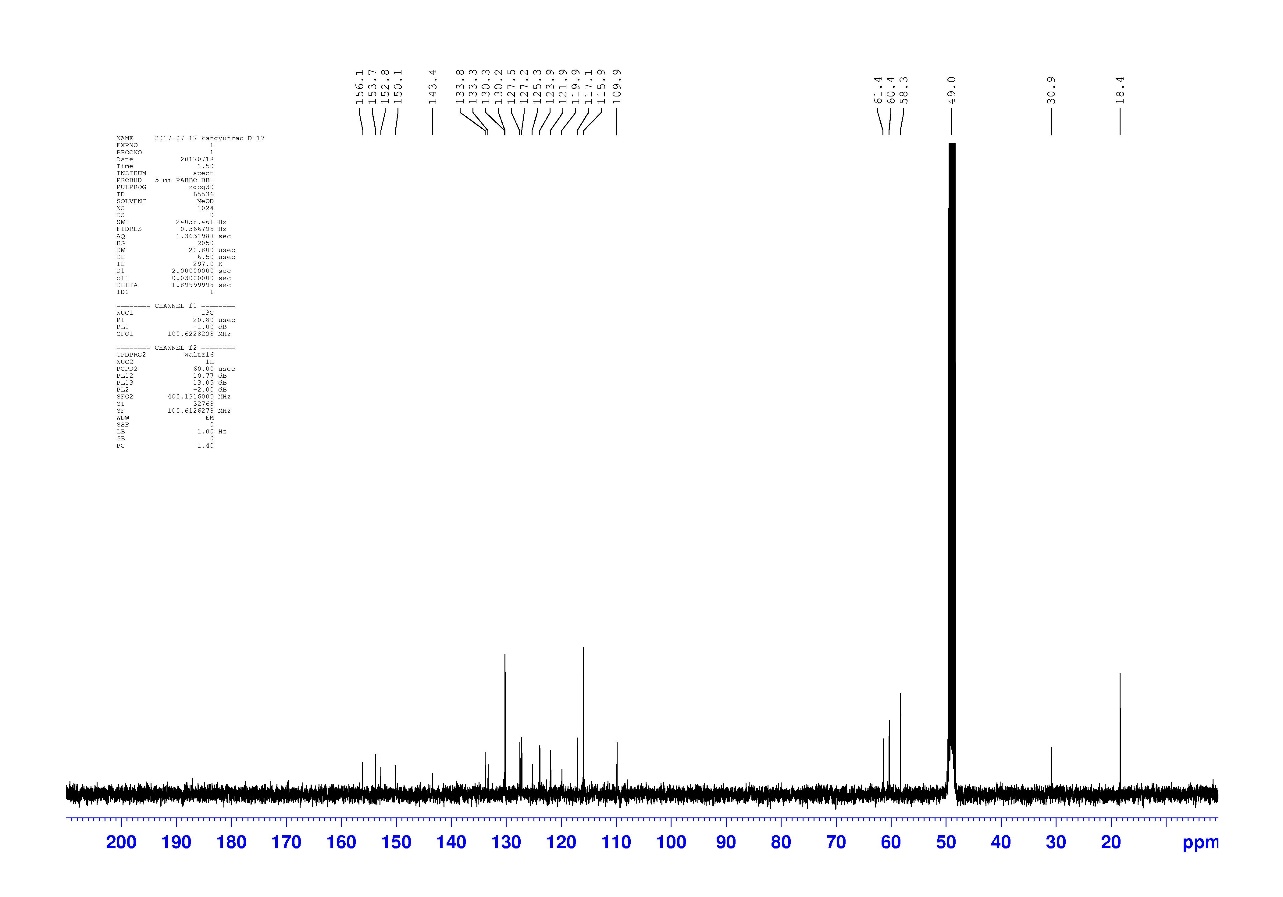


^13^C NMR (100 MHz, CD_3_OD) spectrum of compound **8**

**
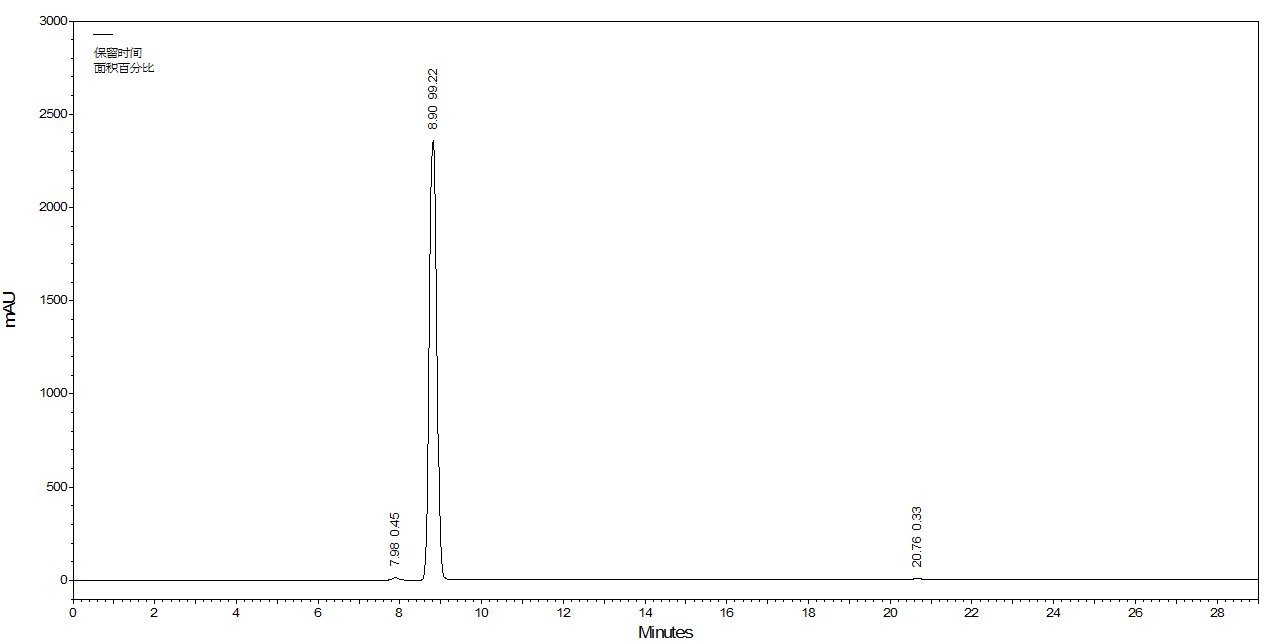
**

HPLC chromatogram of compound **8**


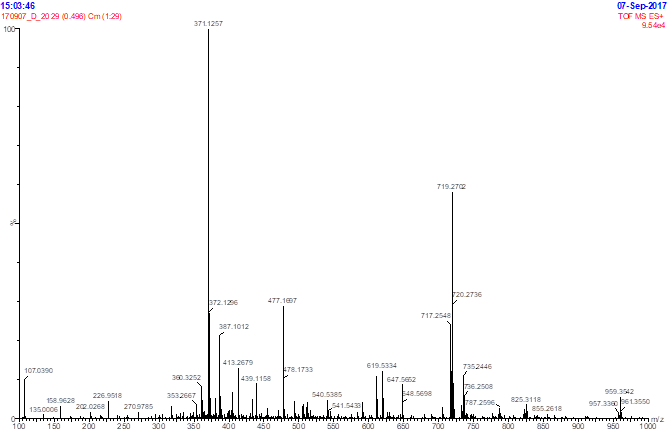


HR-ESI-MS spectrum of compound **9**


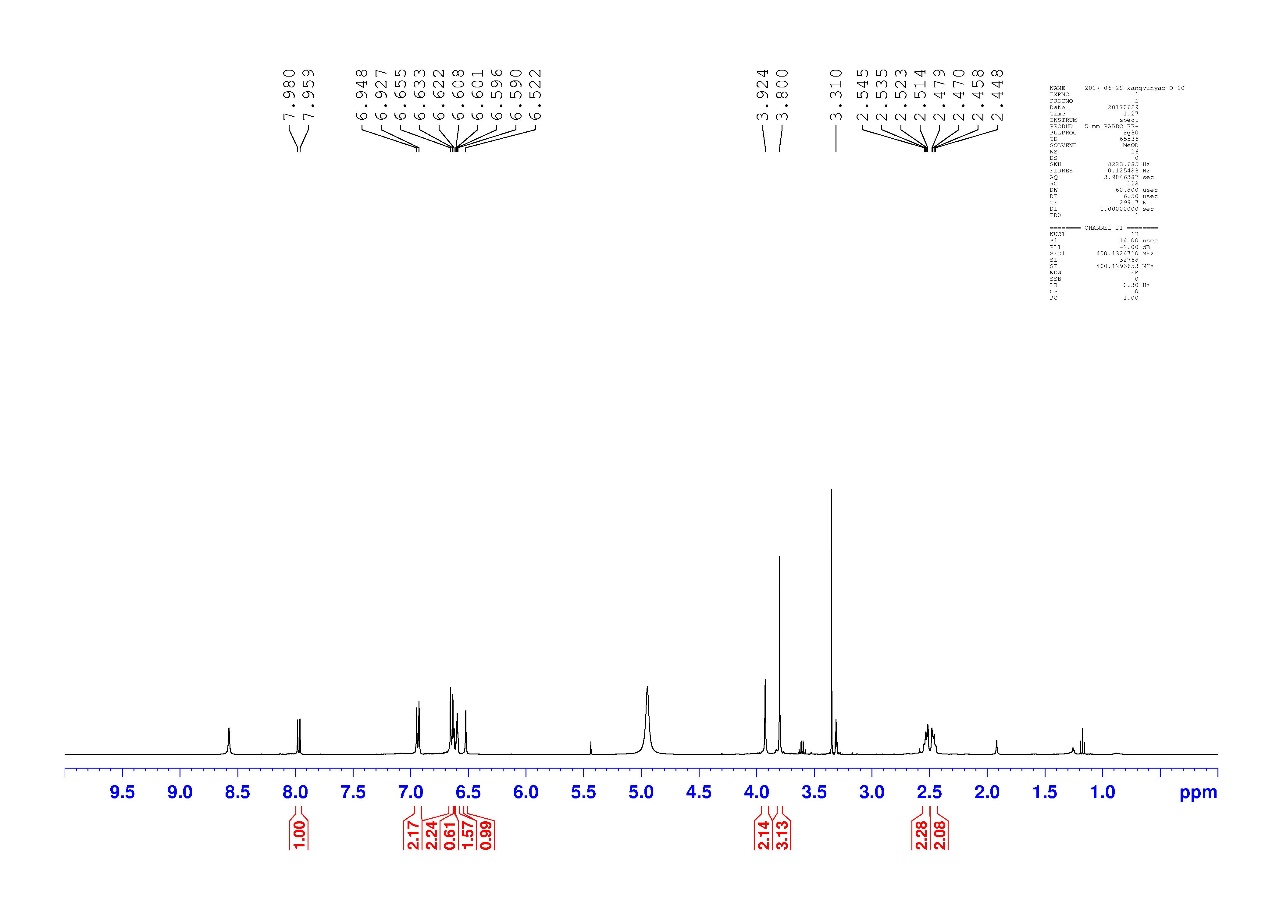


^1^H NMR (400 MHz, CD_3_OD) spectrum of compound **9**


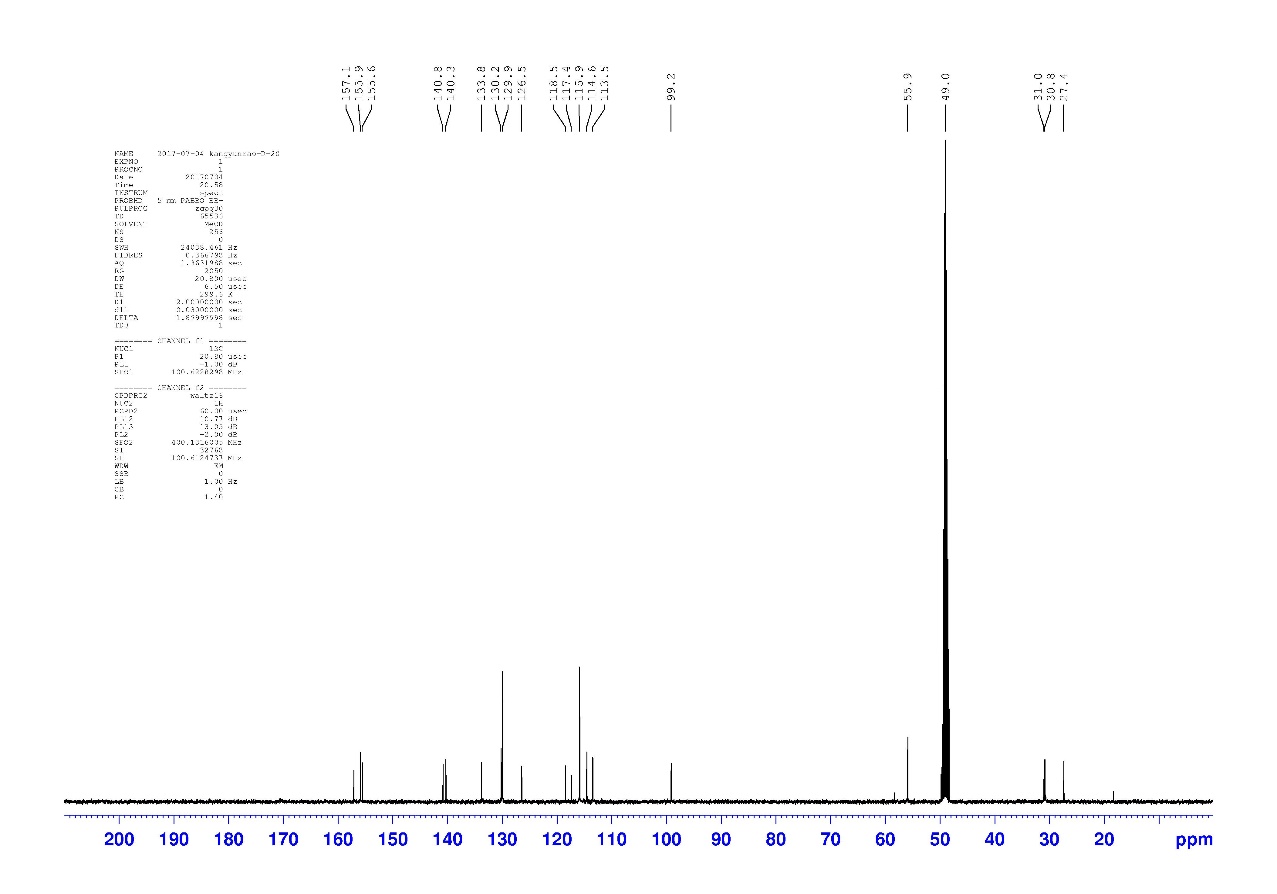


^13^C NMR (100 MHz, CD_3_OD) spectrum of compound **9**

**
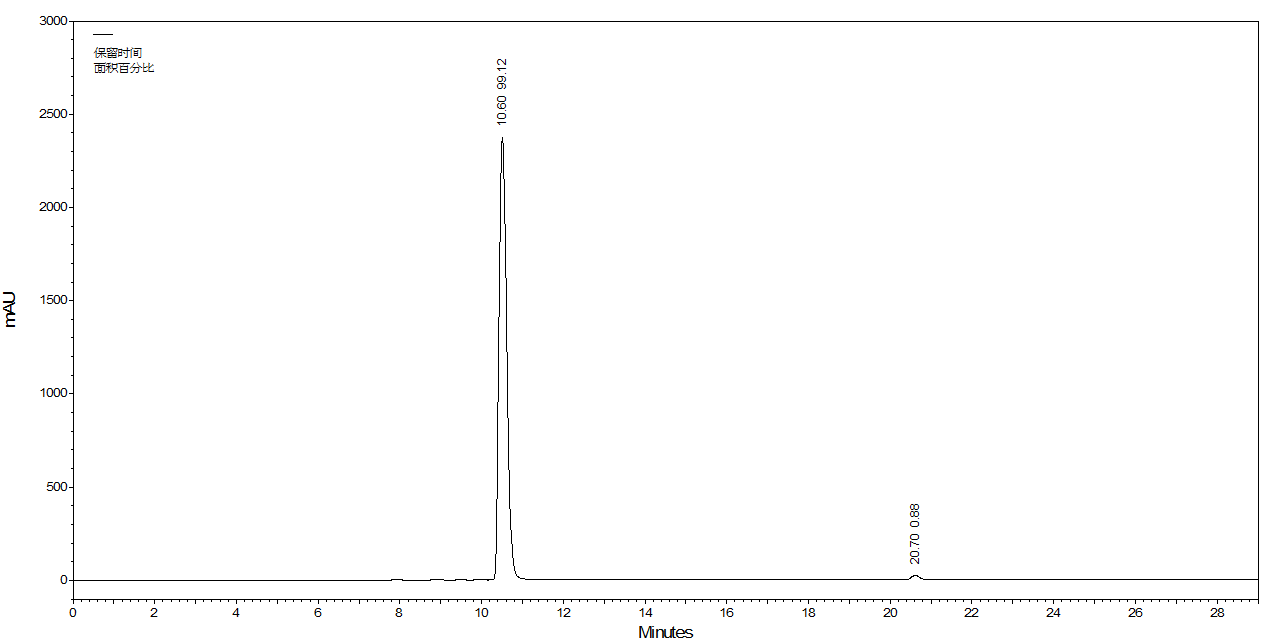
**

HPLC chromatogram of compound **9**

HR-ESI-MS spectrum of compound **10**


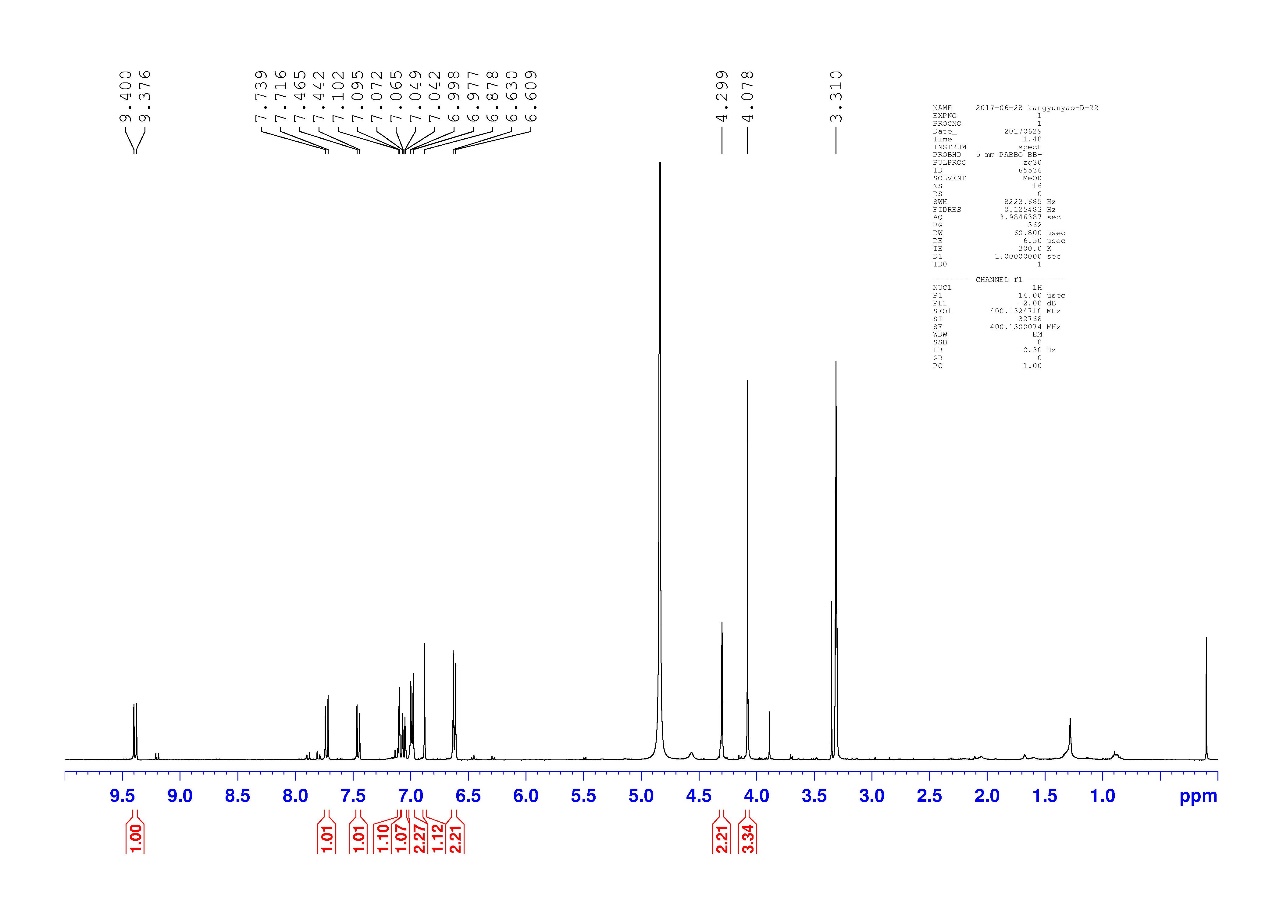


^1^H NMR (400 MHz, CD_3_OD) spectrum of compound **10**


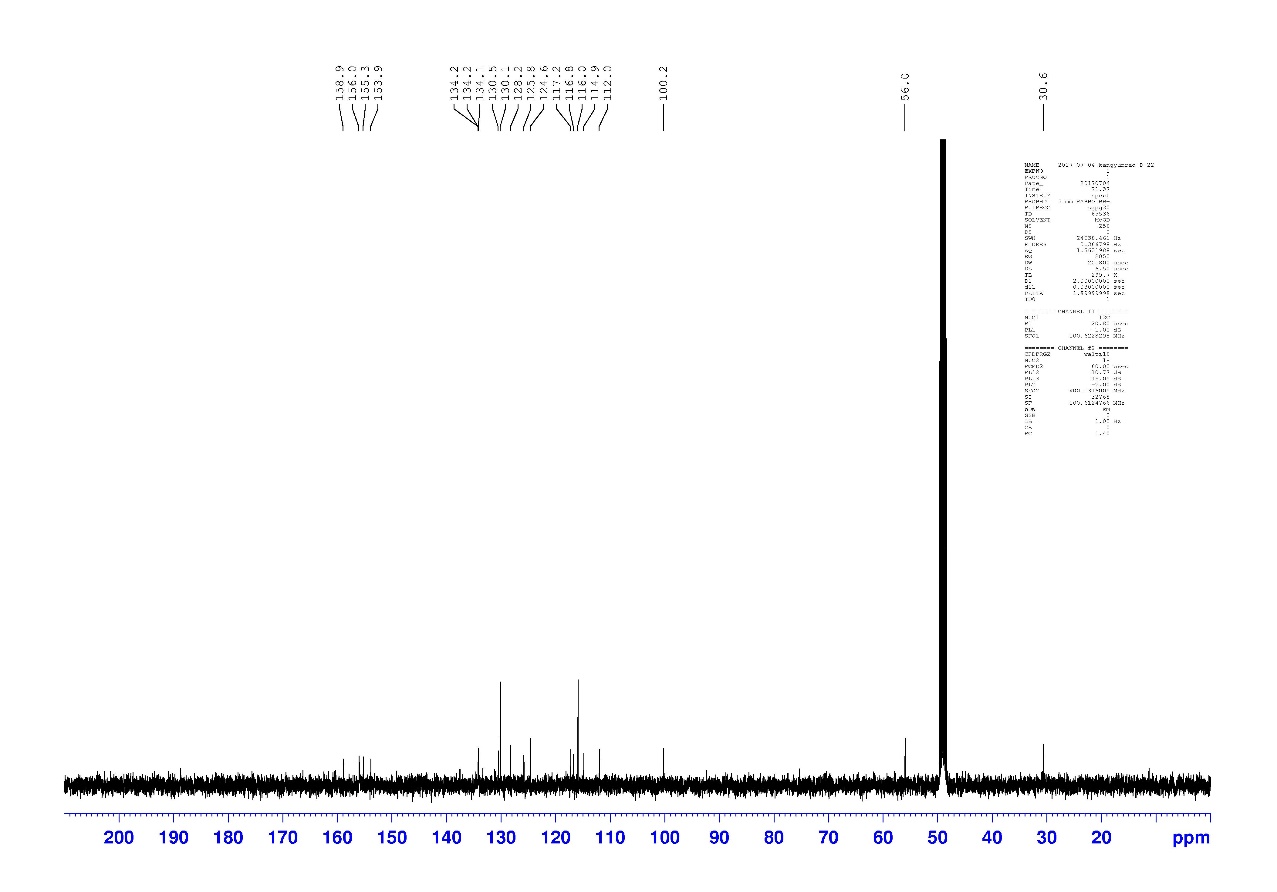


^13^C NMR (100 MHz, CD_3_OD) spectrum of compound **10**

**
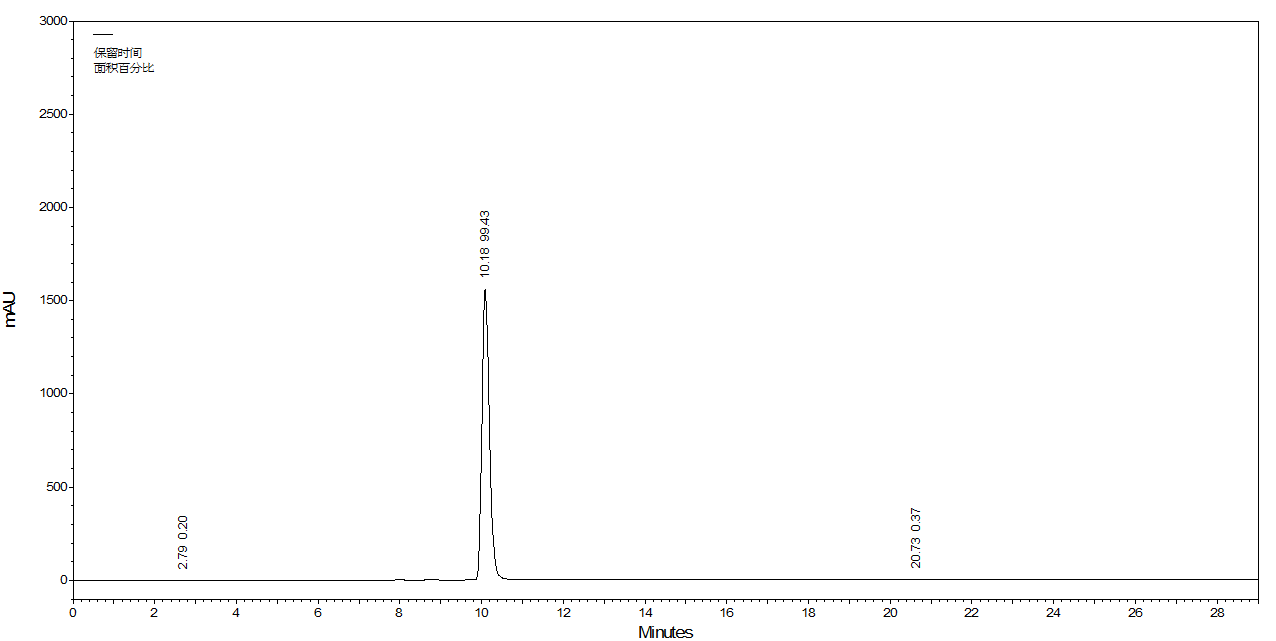
**

HPLC chromatogram of compound **10**


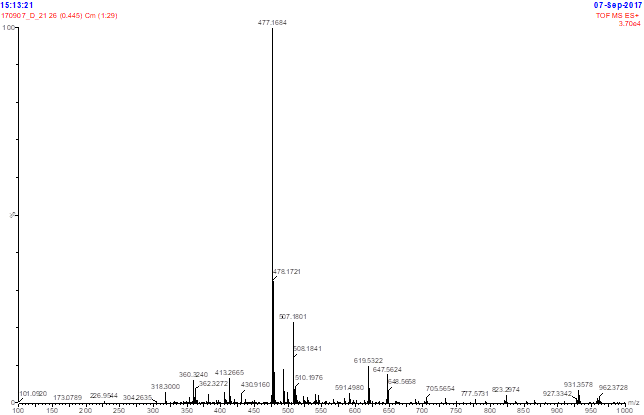


HR-ESI-MS spectrum of compound **11**


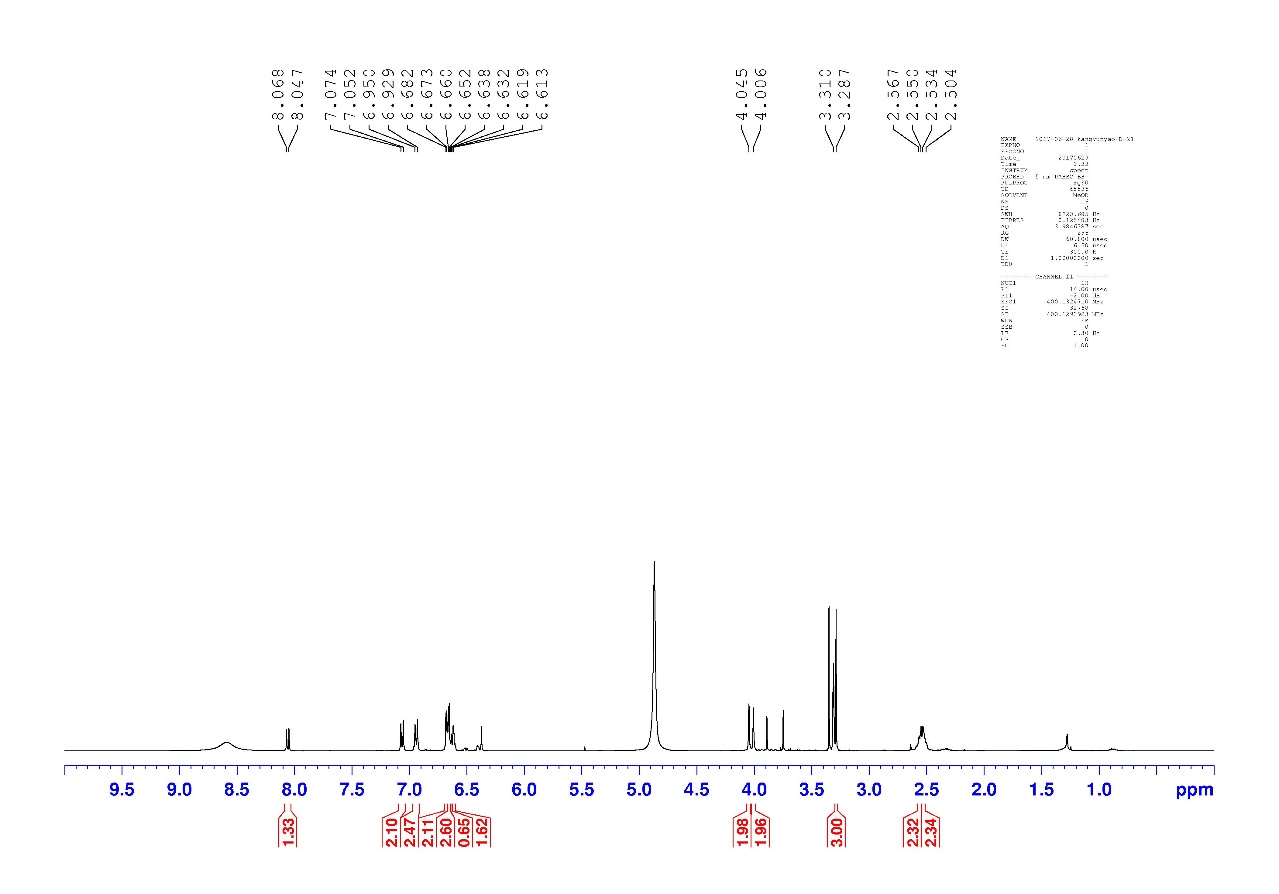


^1^H NMR (400 MHz, CD_3_OD) spectrum of compound **11**


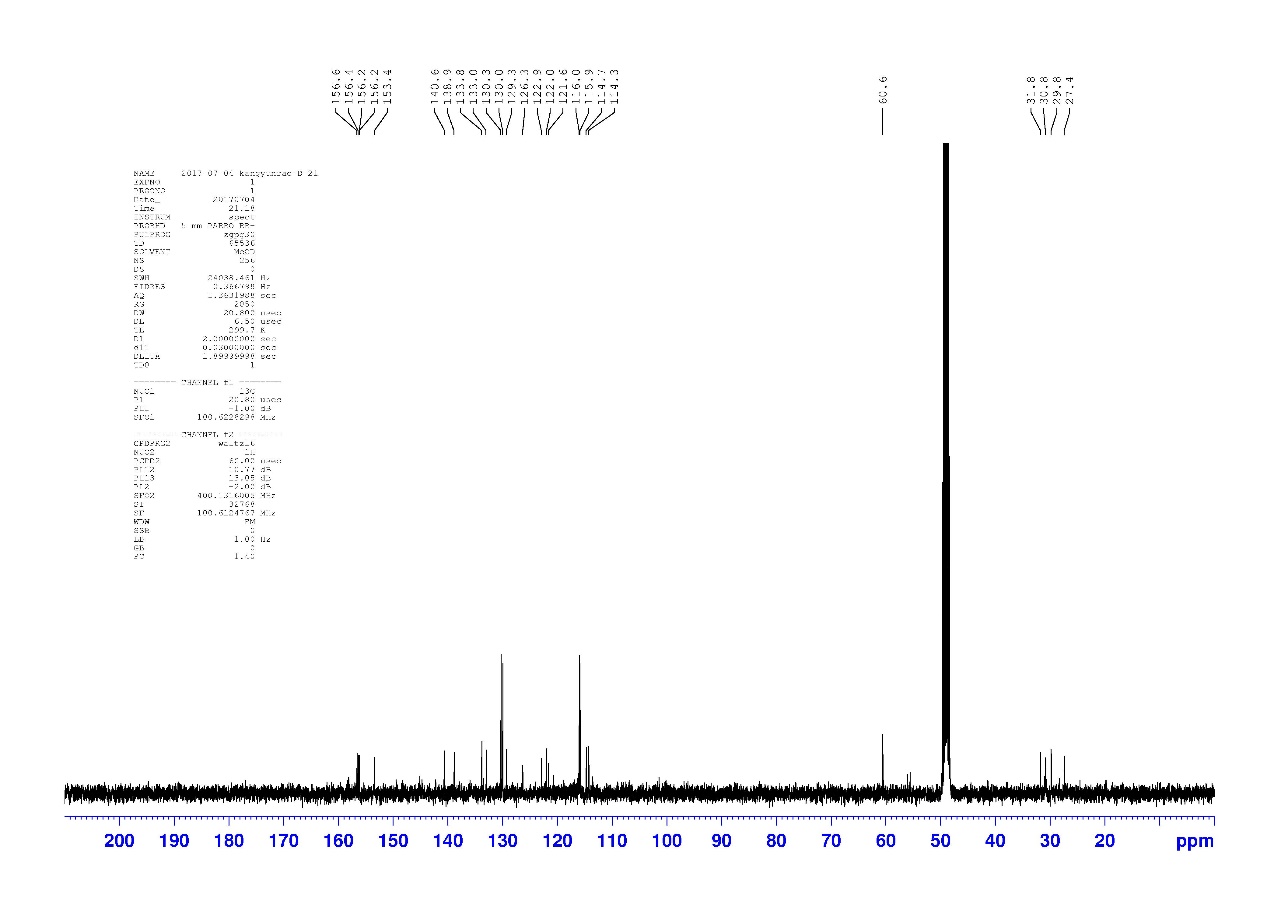


^13^C NMR (100 MHz, CD_3_OD) spectrum of compound **11**


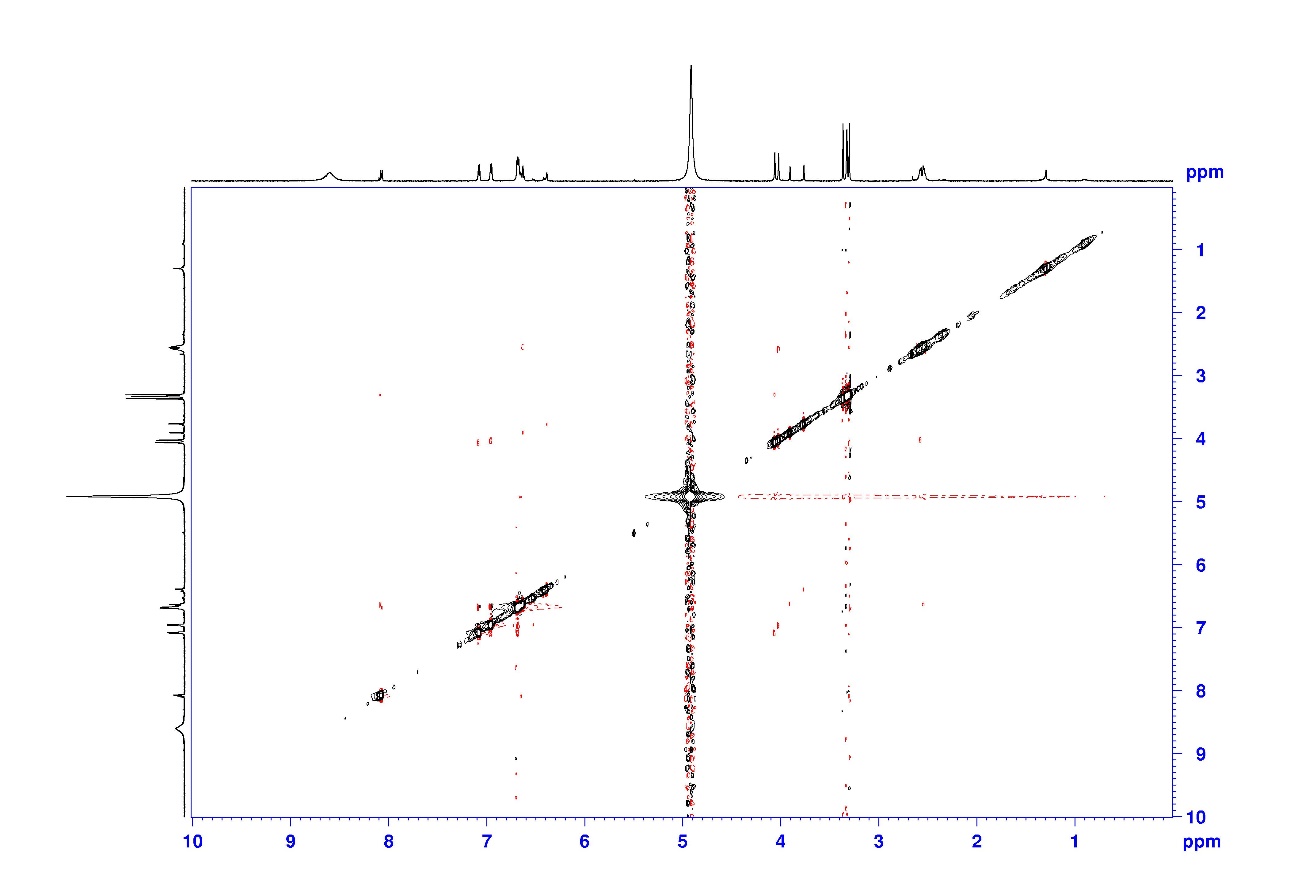


NOESY spectrum of compound **11**

**
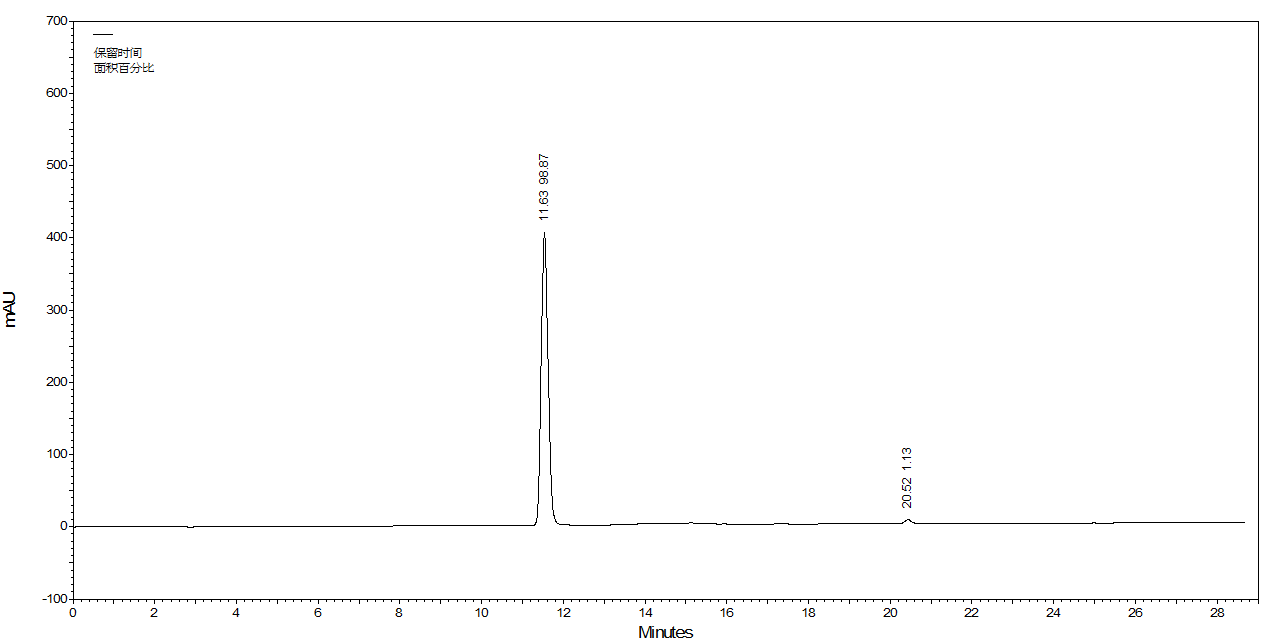
**

HPLC chromatogram of compound 11

HR-ESI-MS spectrum of compound **12**


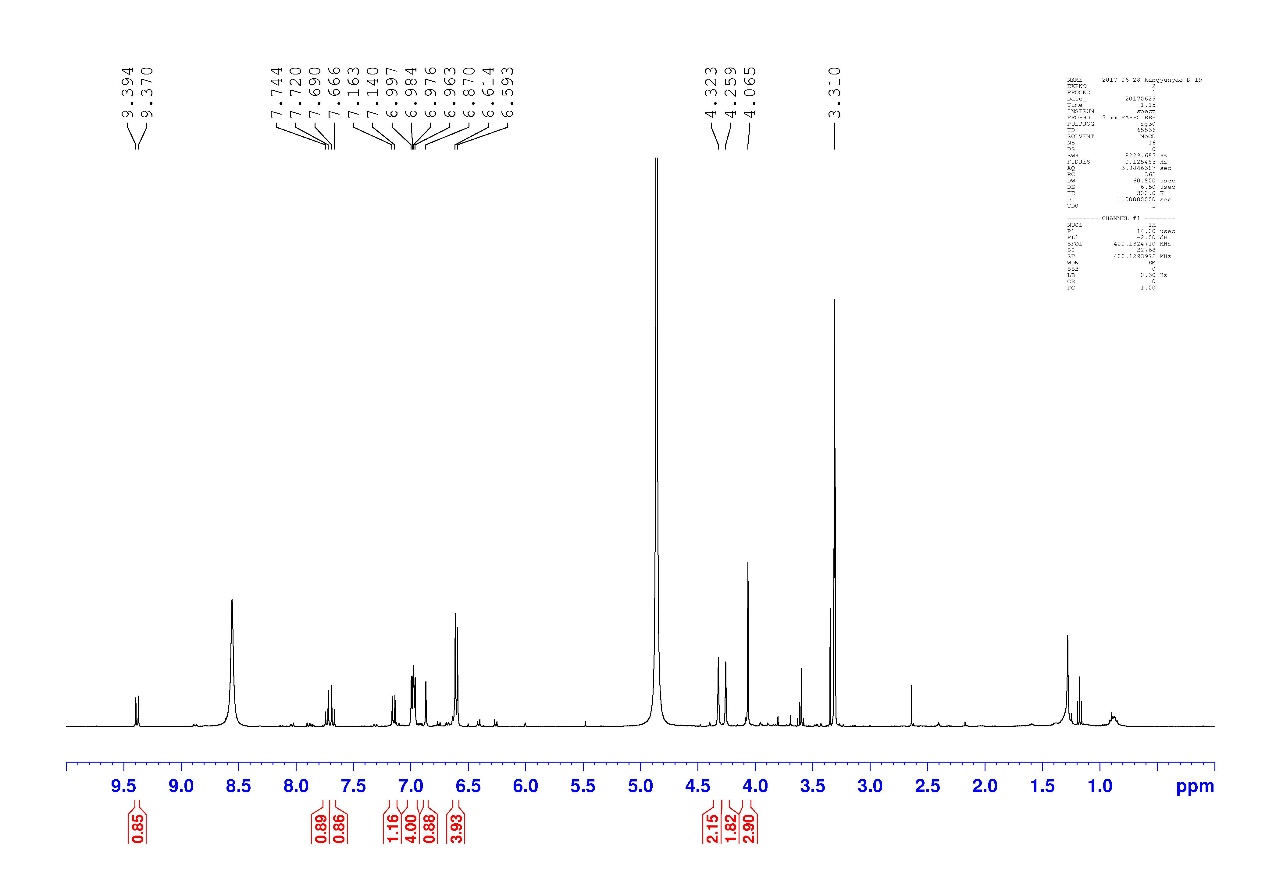


^1^H NMR (400 MHz, CD_3_OD) spectrum of compound **12**

**
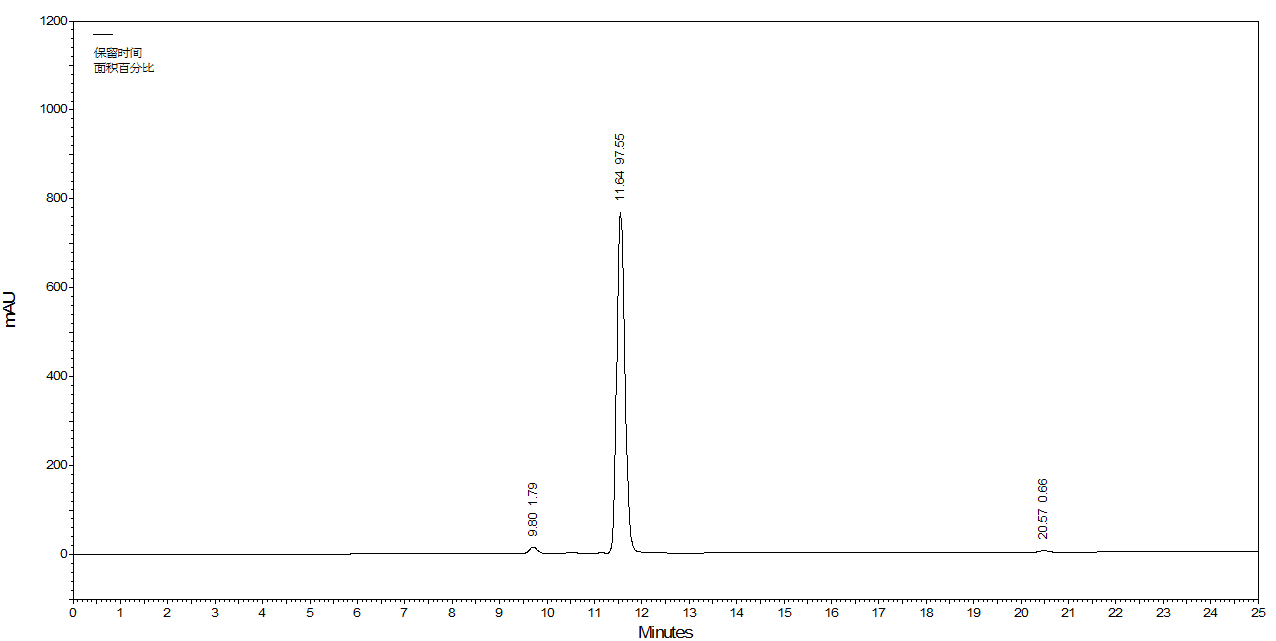
**

HPLC chromatogram of compound 1**2**

HR-ESI-MS spectrum of compound **13**


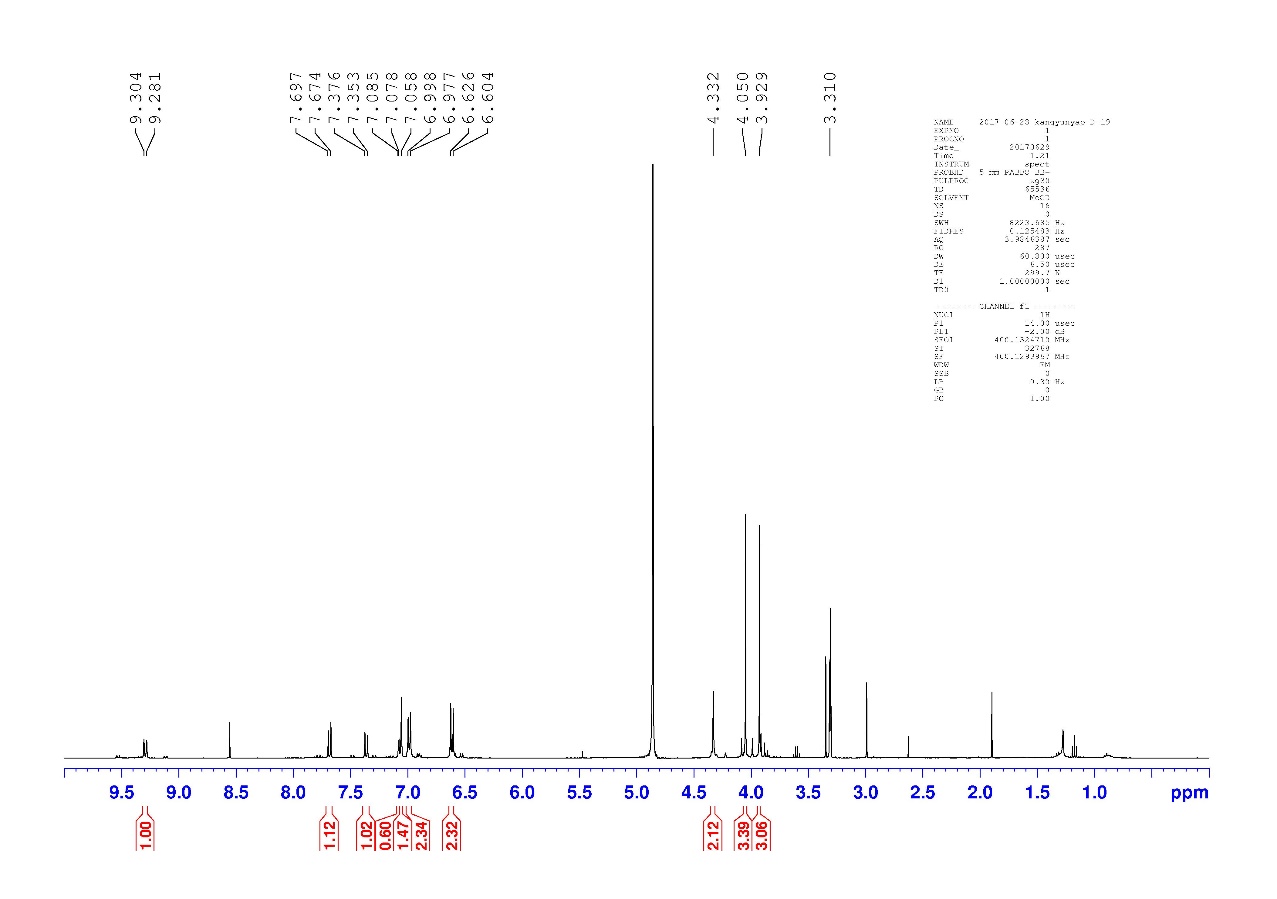


^1^H NMR (400 MHz, CD_3_OD) spectrum of compound **13**


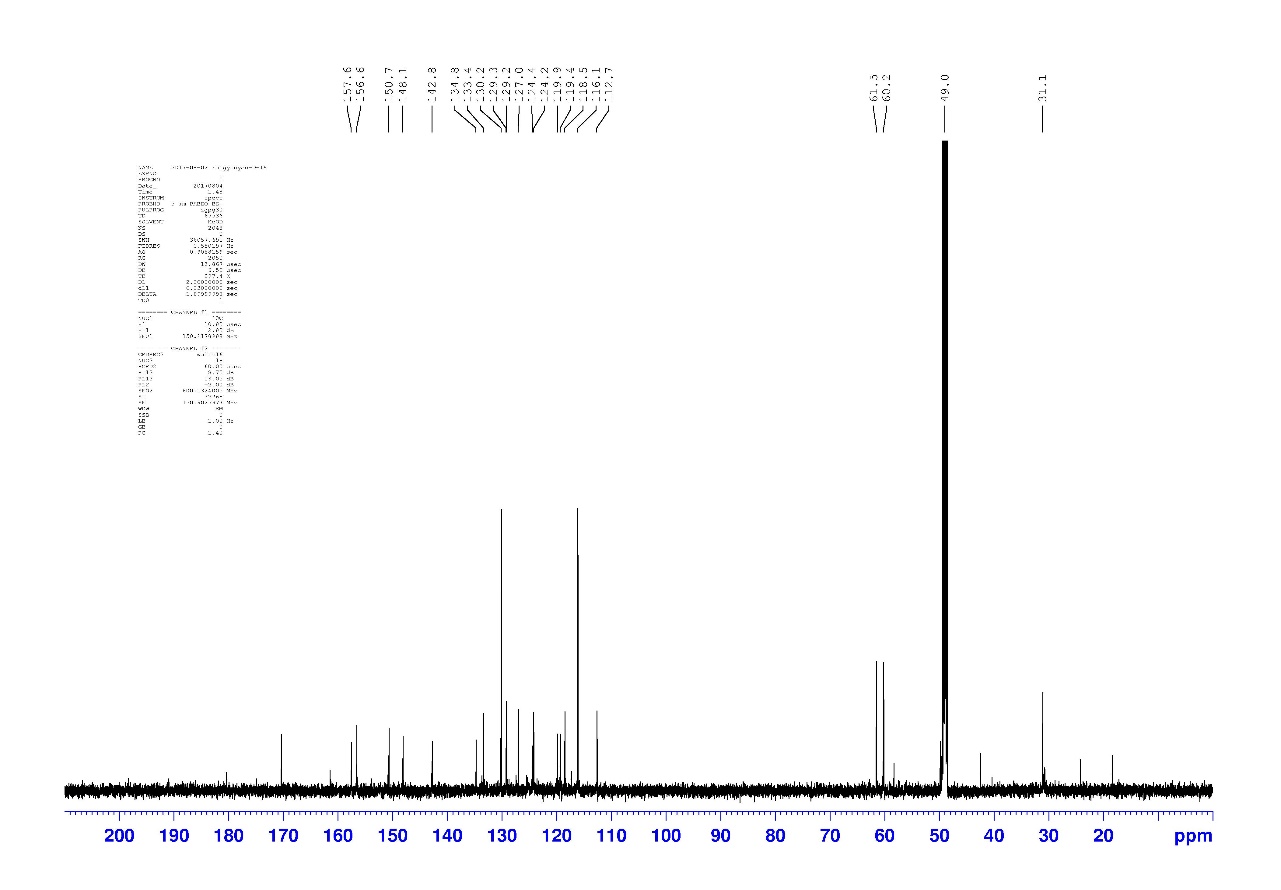


^13^C NMR (100 MHz, CD_3_OD) spectrum of compound **13**


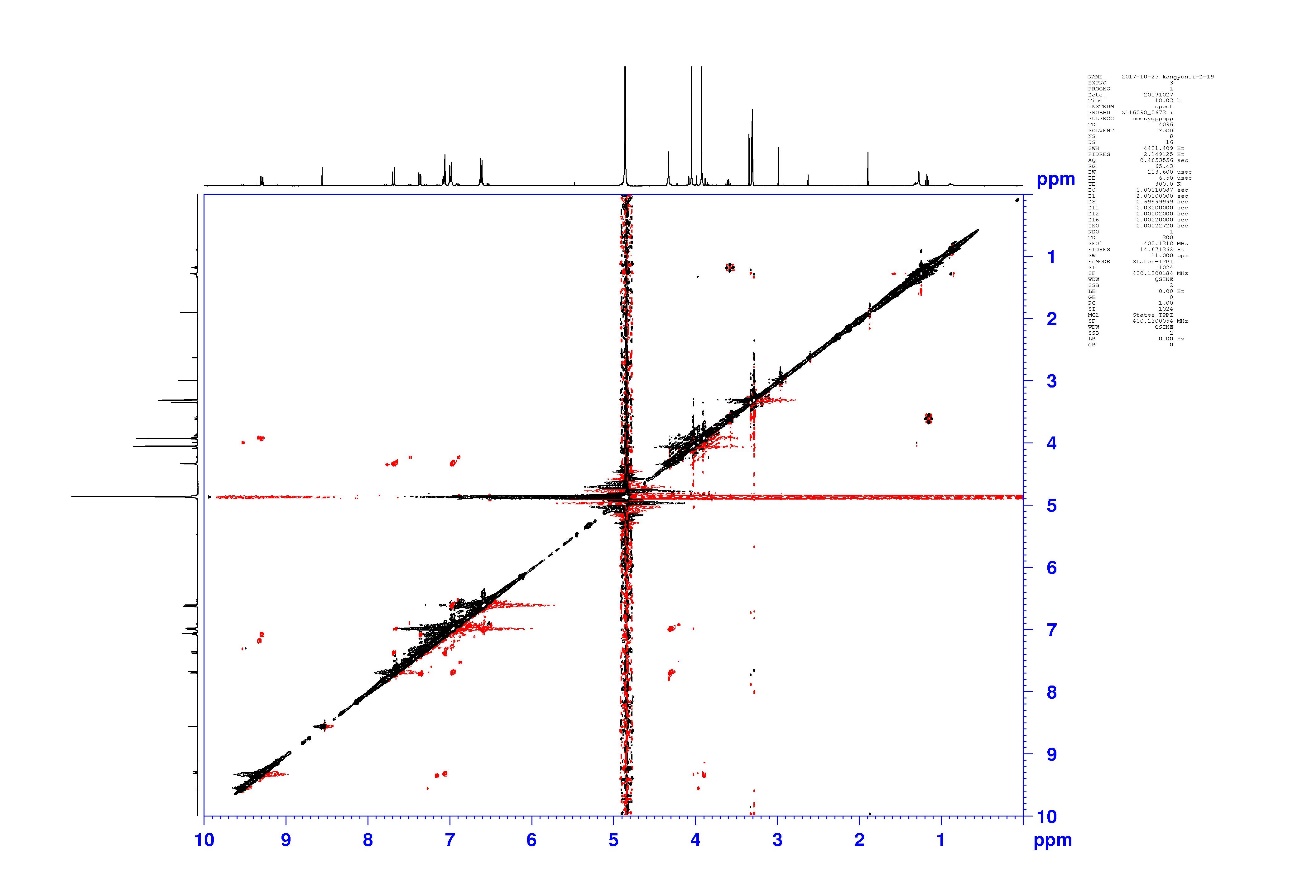


NOESY spectrum of compound **13**

**
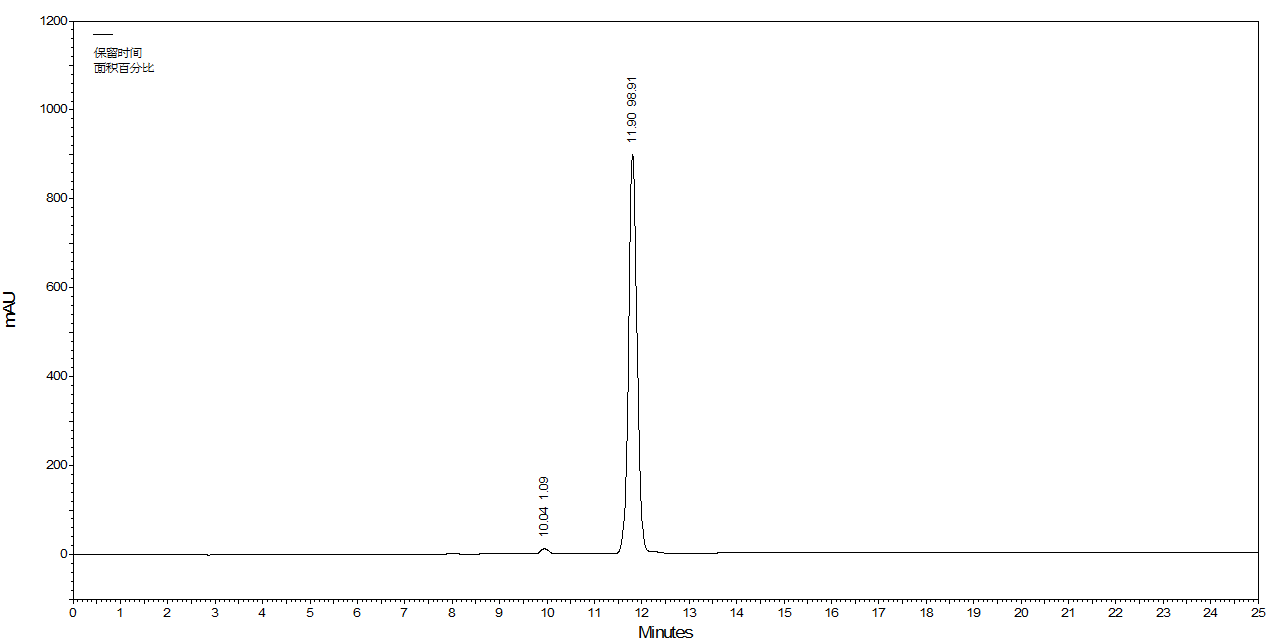
**

HPLC chromatogram of compound **13**

HR-ESI-MS spectrum of compound **14**


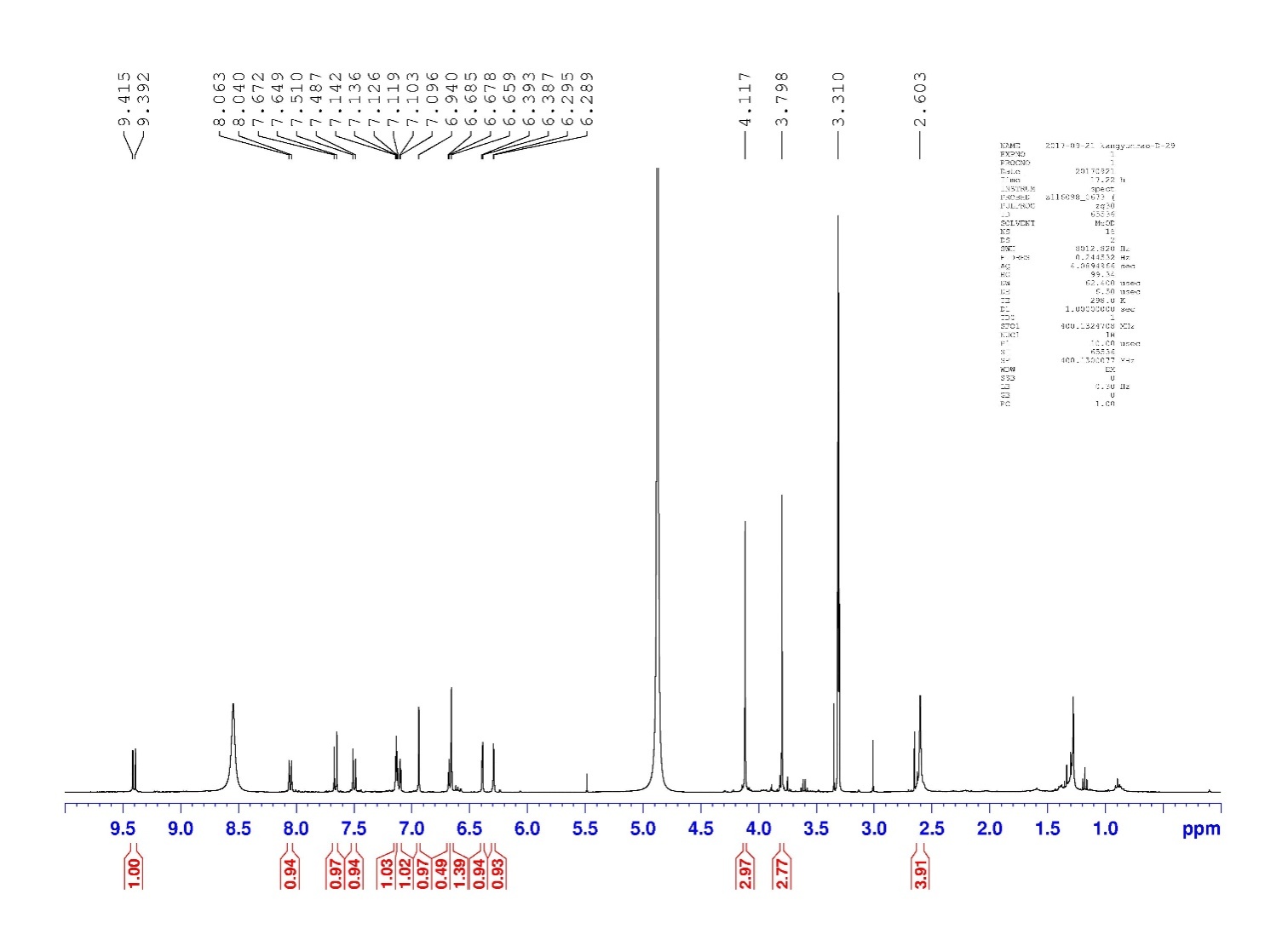


^1^H NMR (400 MHz, CD_3_OD) spectrum of compound **14**


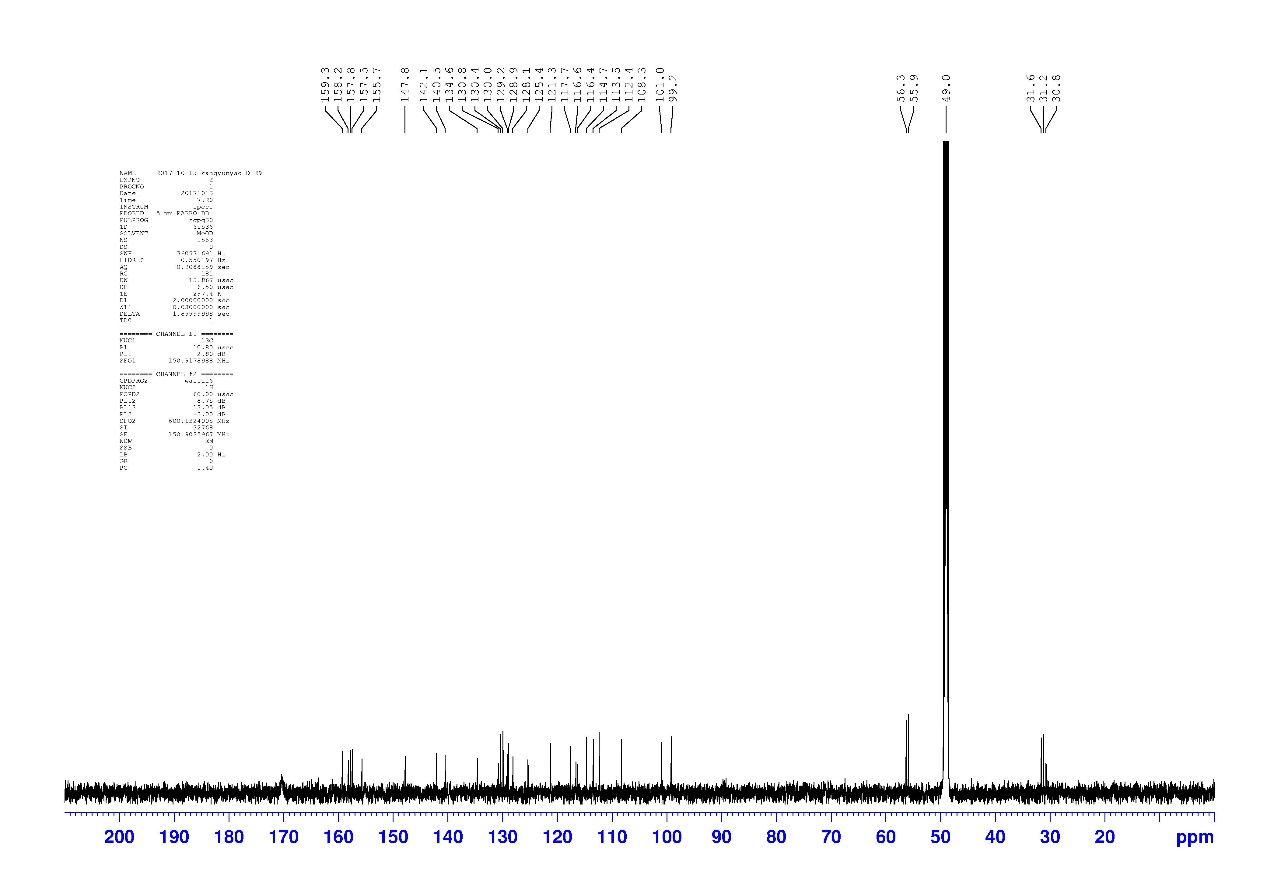


^13^C NMR (100 MHz, CD_3_OD)spectrum of compound **14**

**
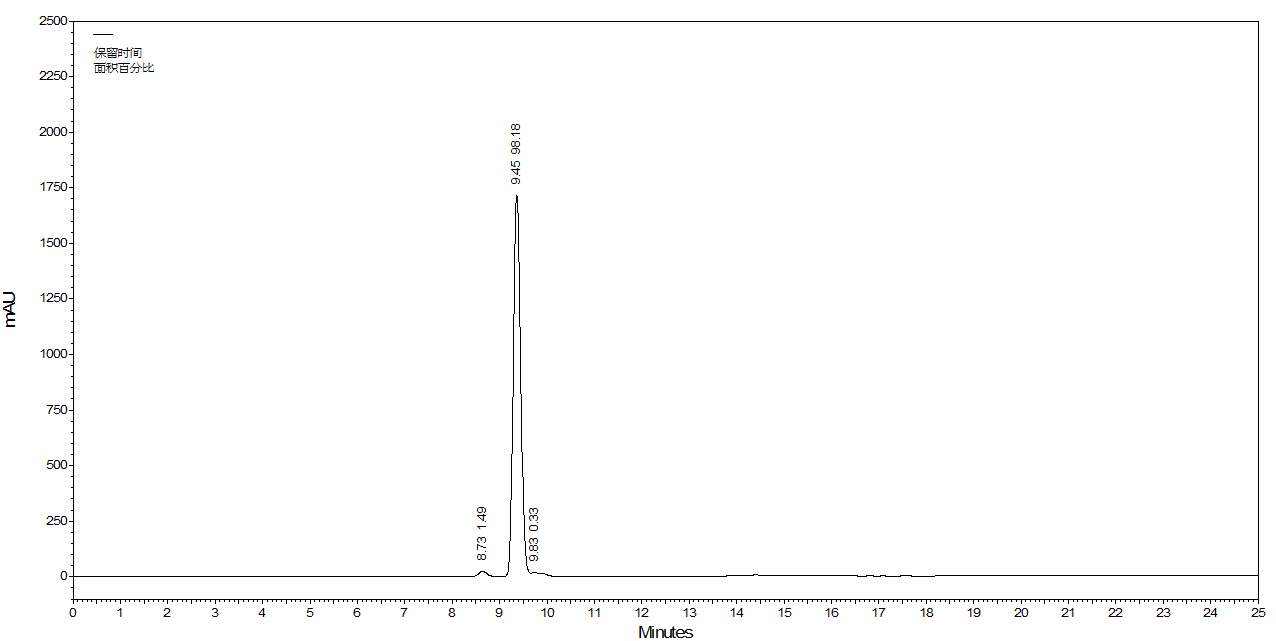
**

HPLC chromatogram of compound **14**

HR-ESI-MS spectrum of compound **15**


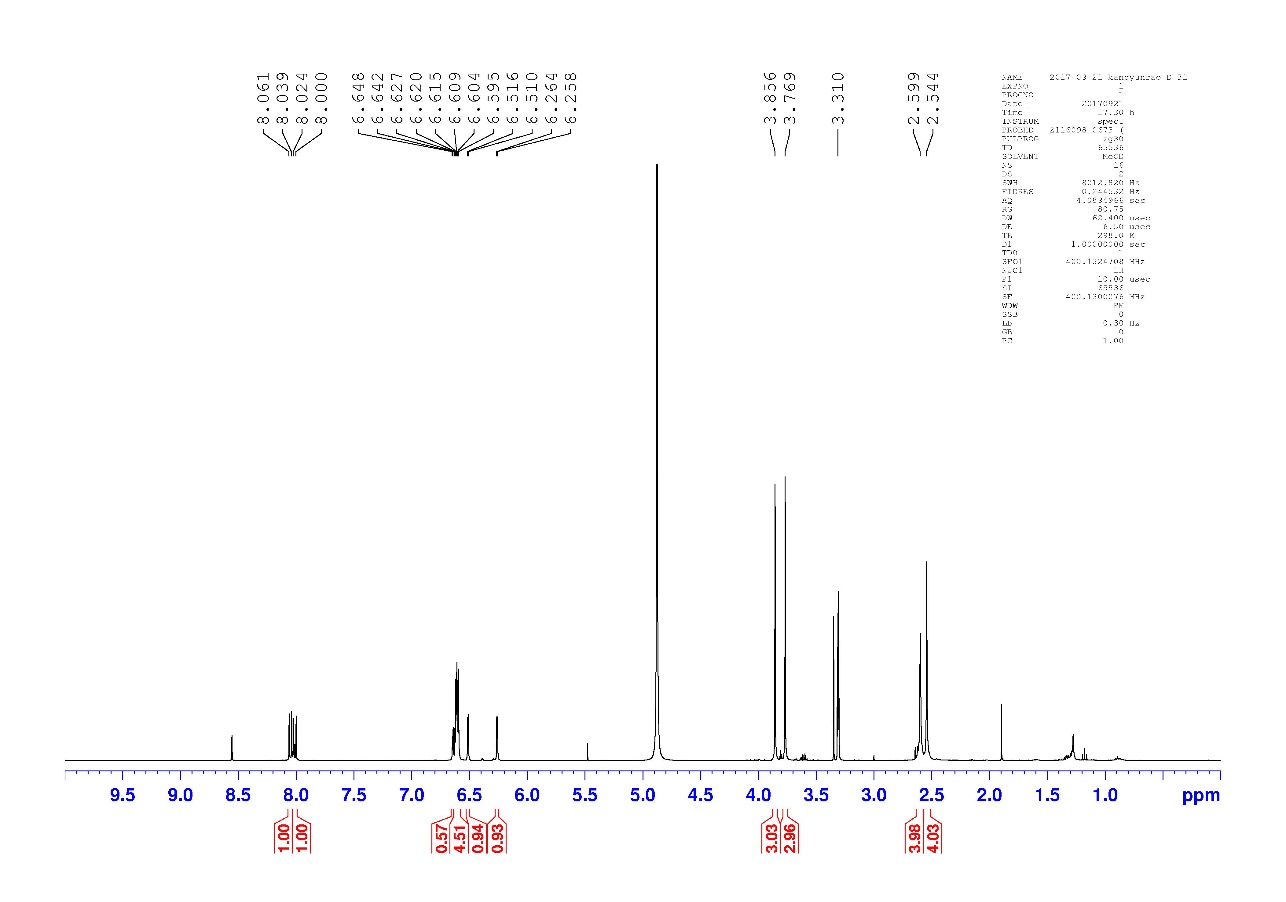


^1^H NMR (400 MHz, CD_3_OD) spectrum of compound **15**


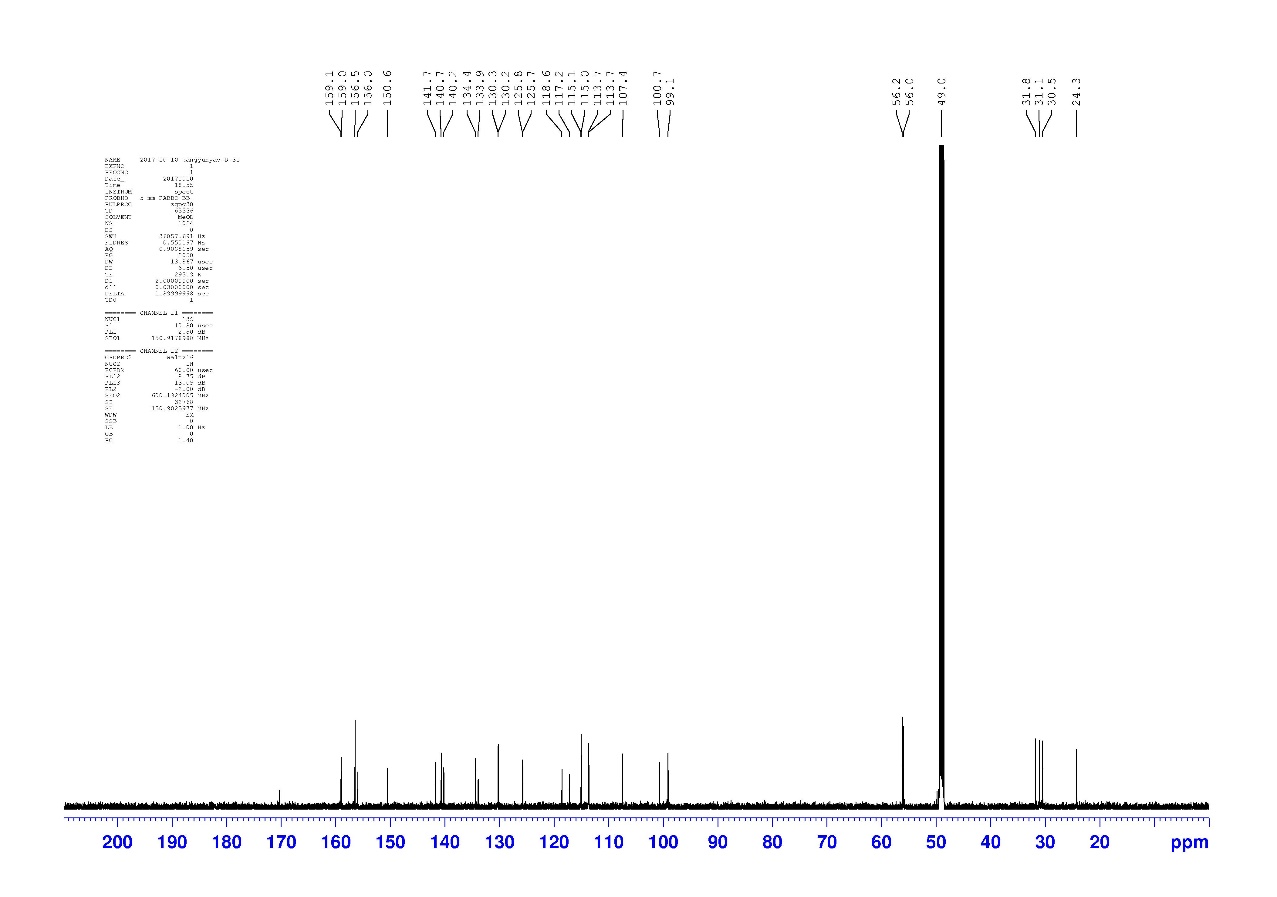


^13^C NMR (100 MHz, CD_3_OD) spectrum of compound **15**


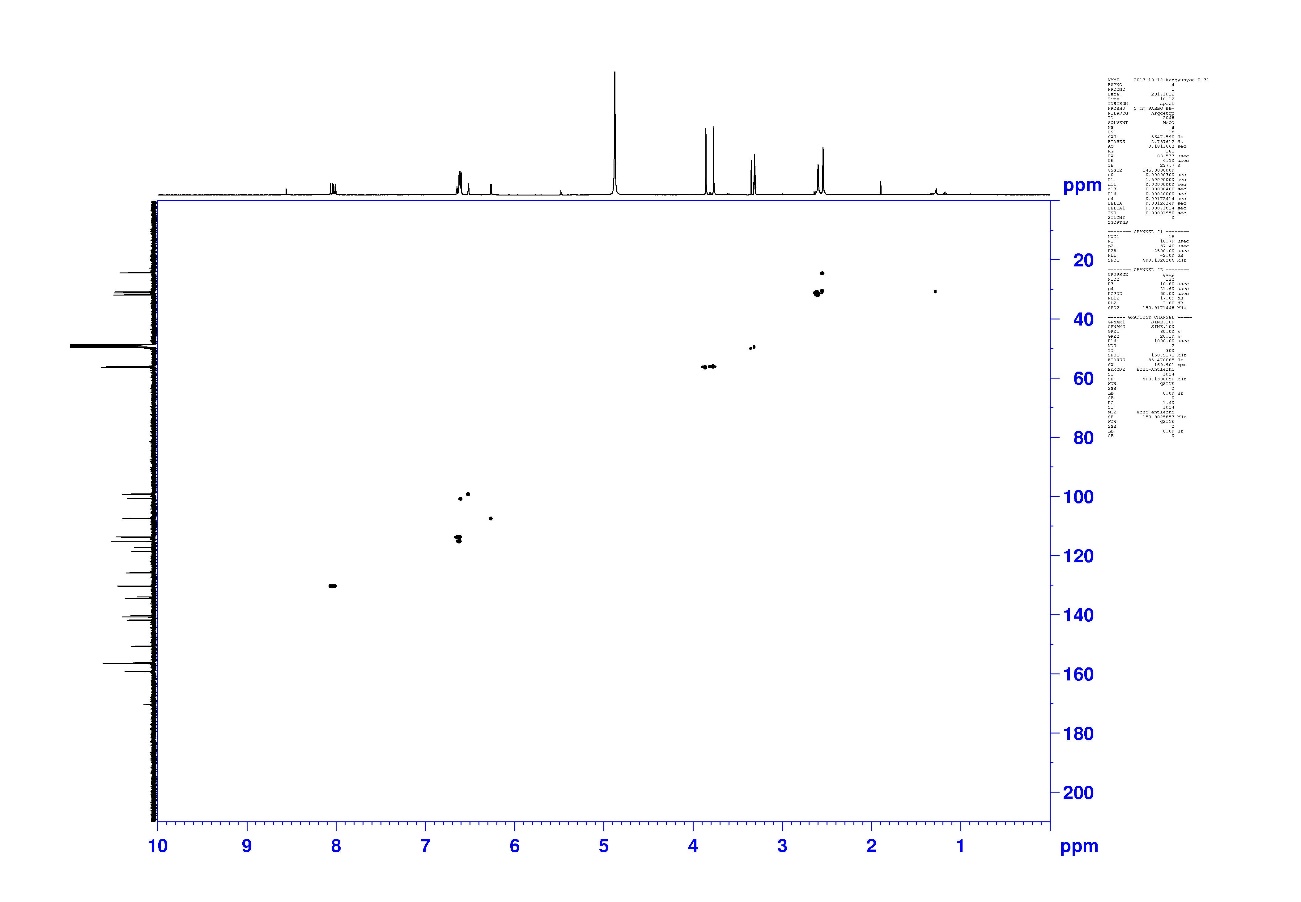


HSQC spectrum of compound **15**


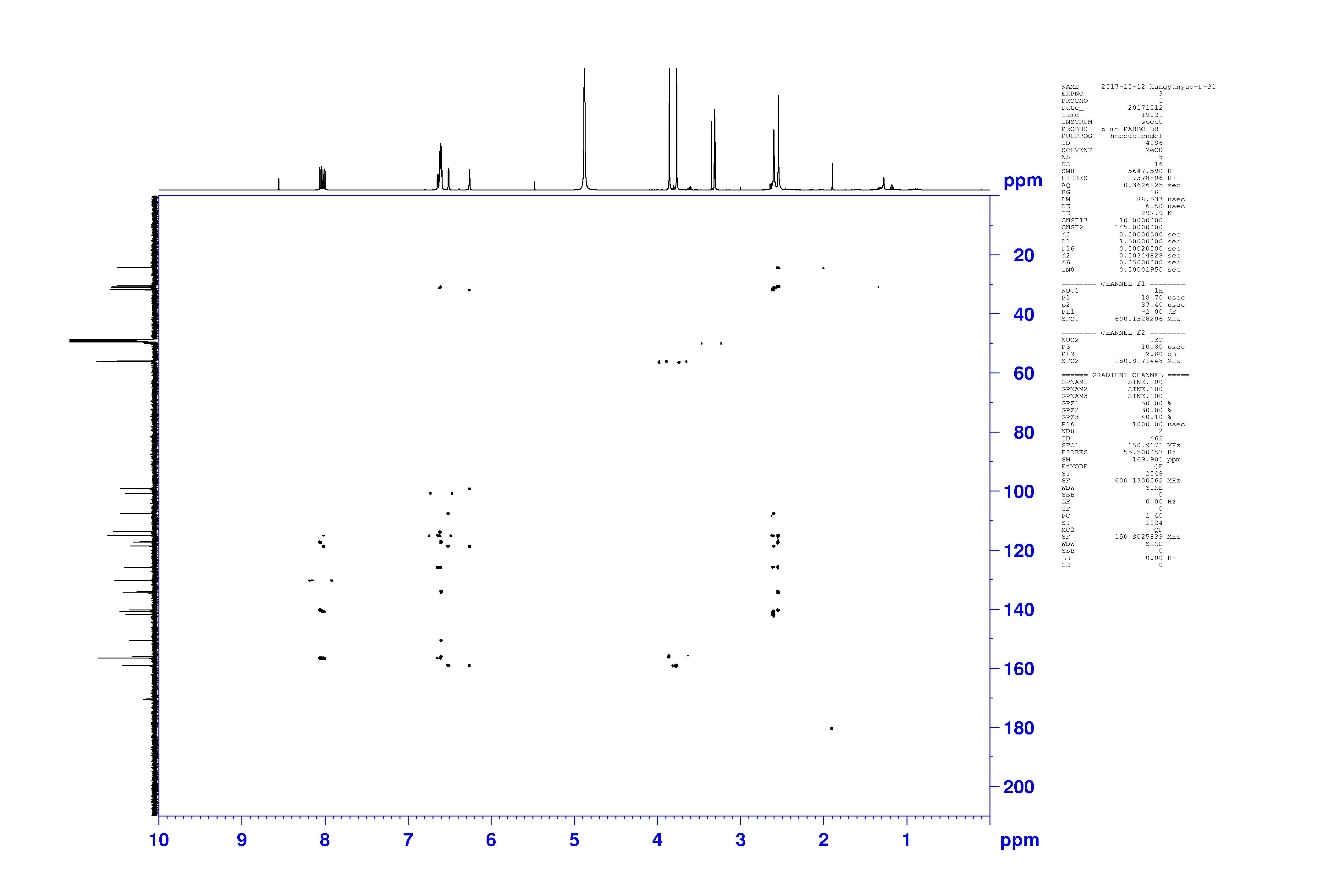


HMBC spectrum of compound **15**


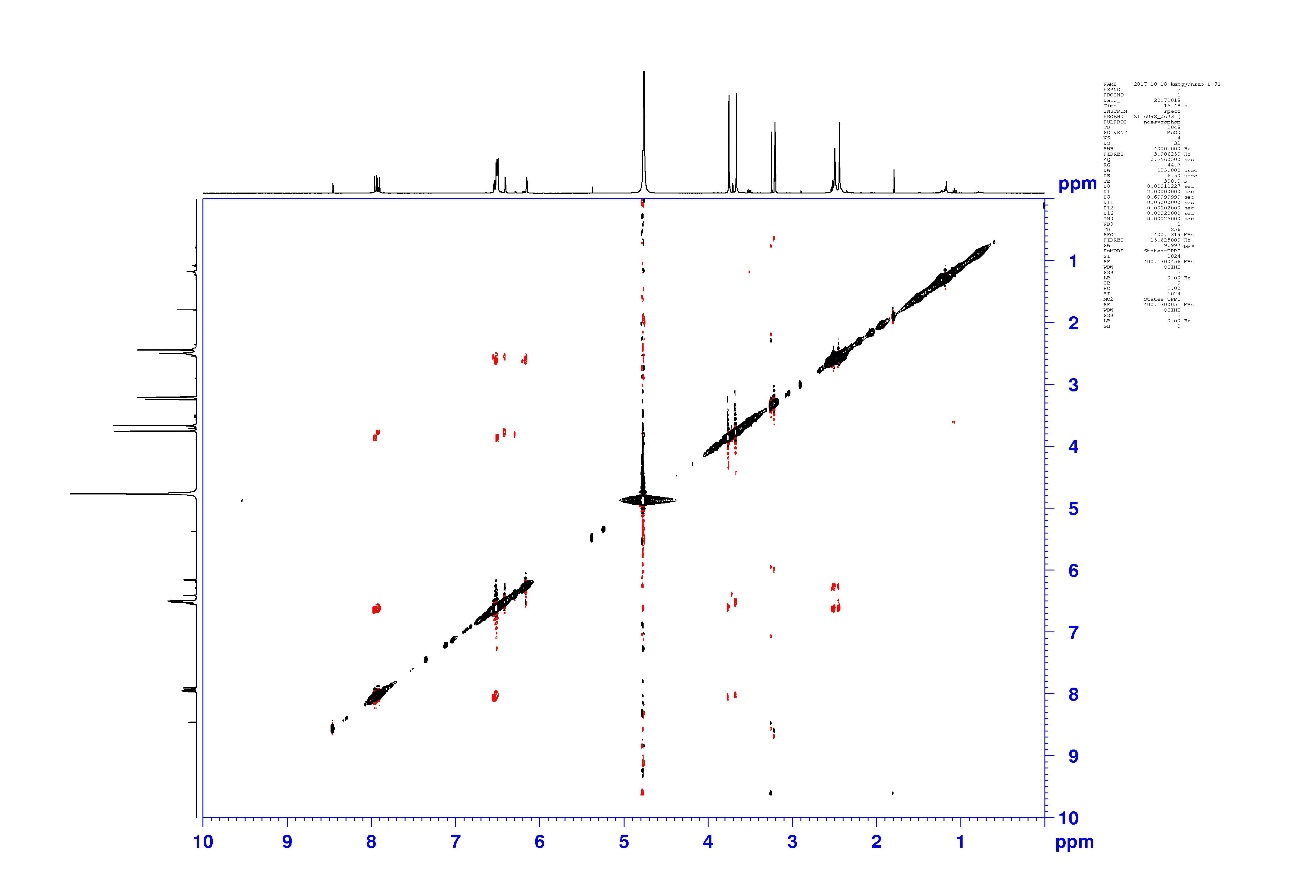


NOESY spectrum of compound **15**


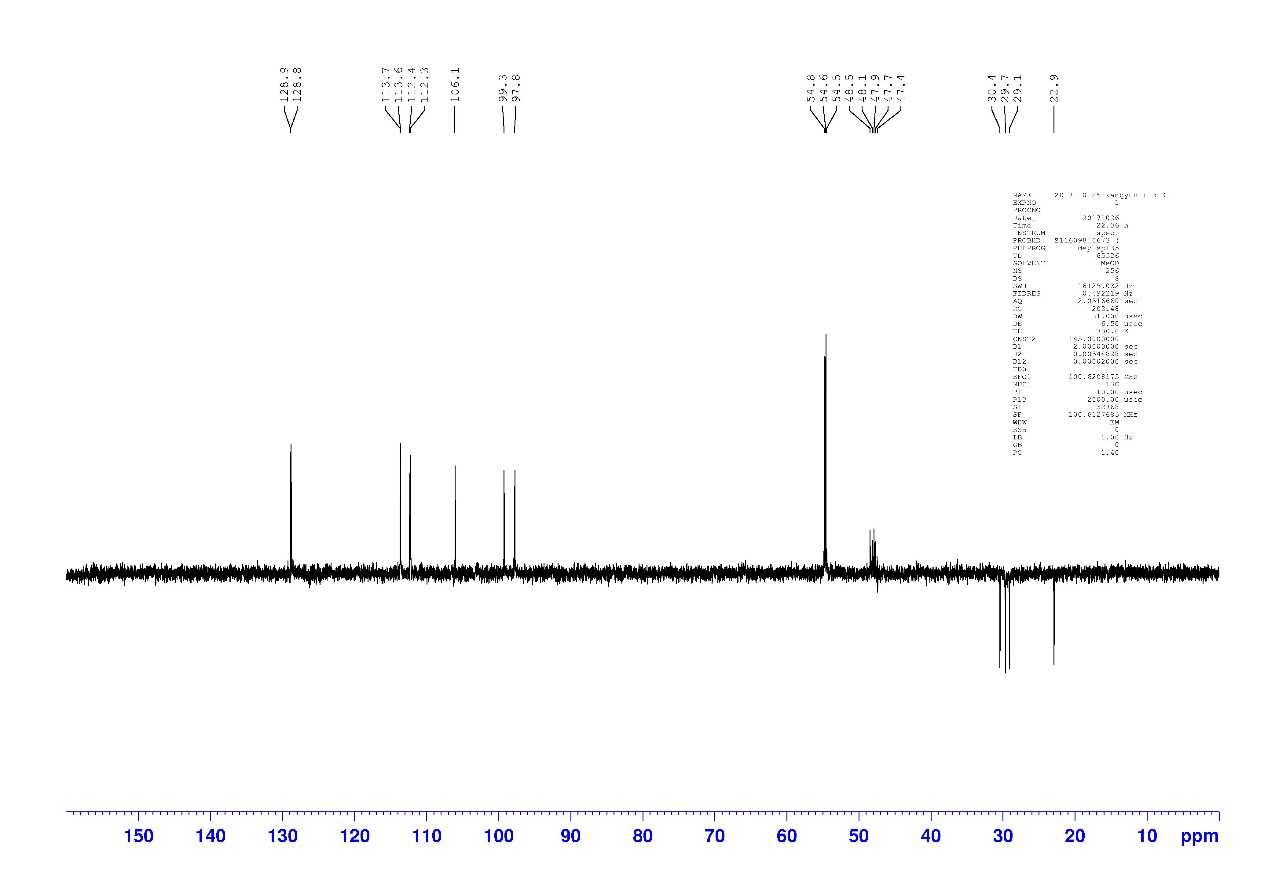


Dept 135 spectrum of compound **15**


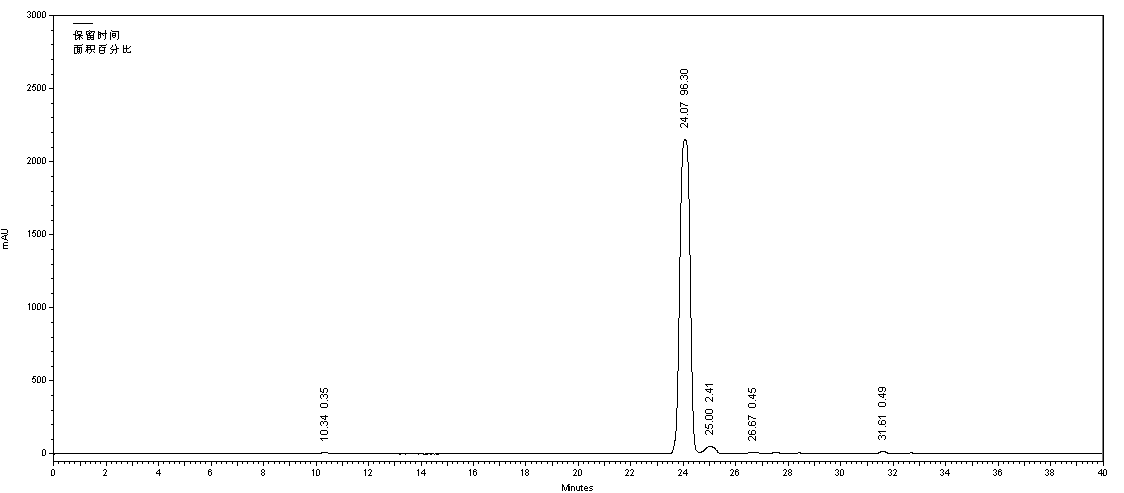


HPLC chromatogram of compound **15**

HR-ESI-MS spectrum of compound **16**


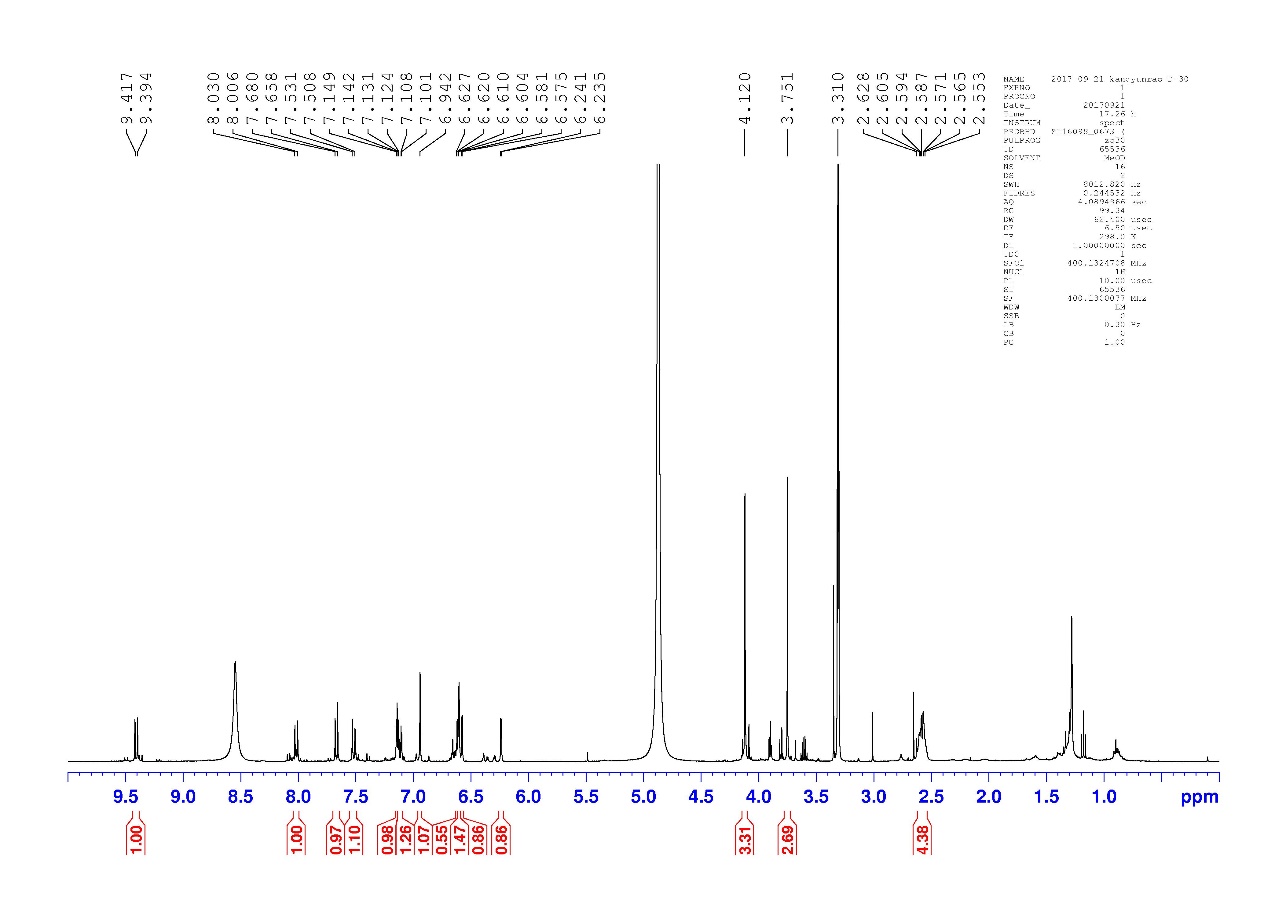


^1^H NMR (400 MHz, CD_3_OD) spectrum of compound **16**

**
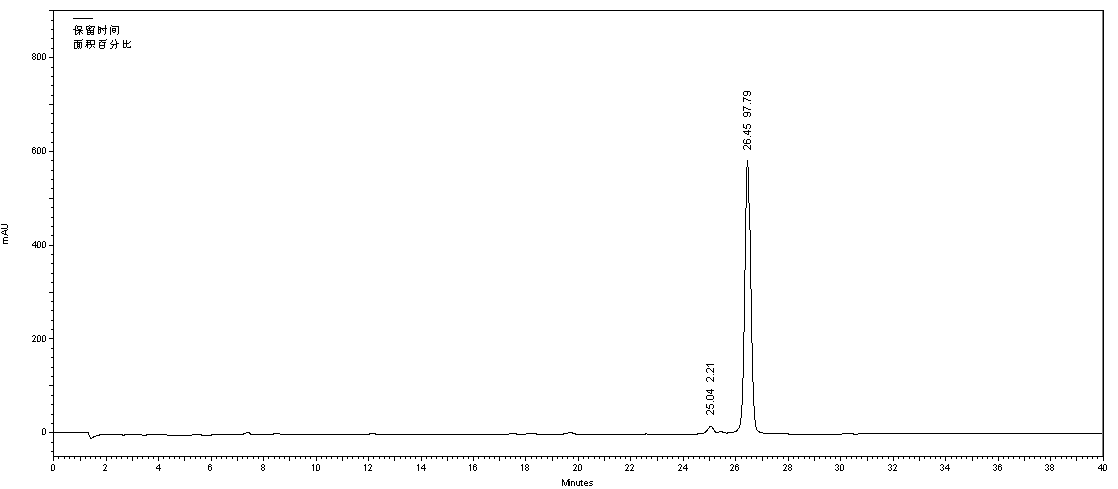
**

HPLC chromatogram of compound **16**


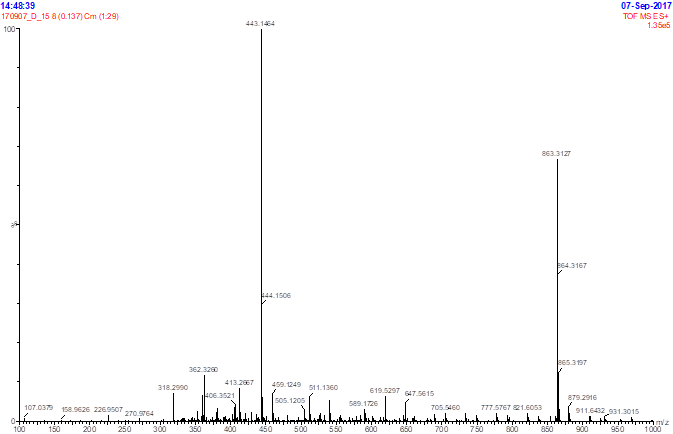


HR-ESI-MS spectrum of compound **17**


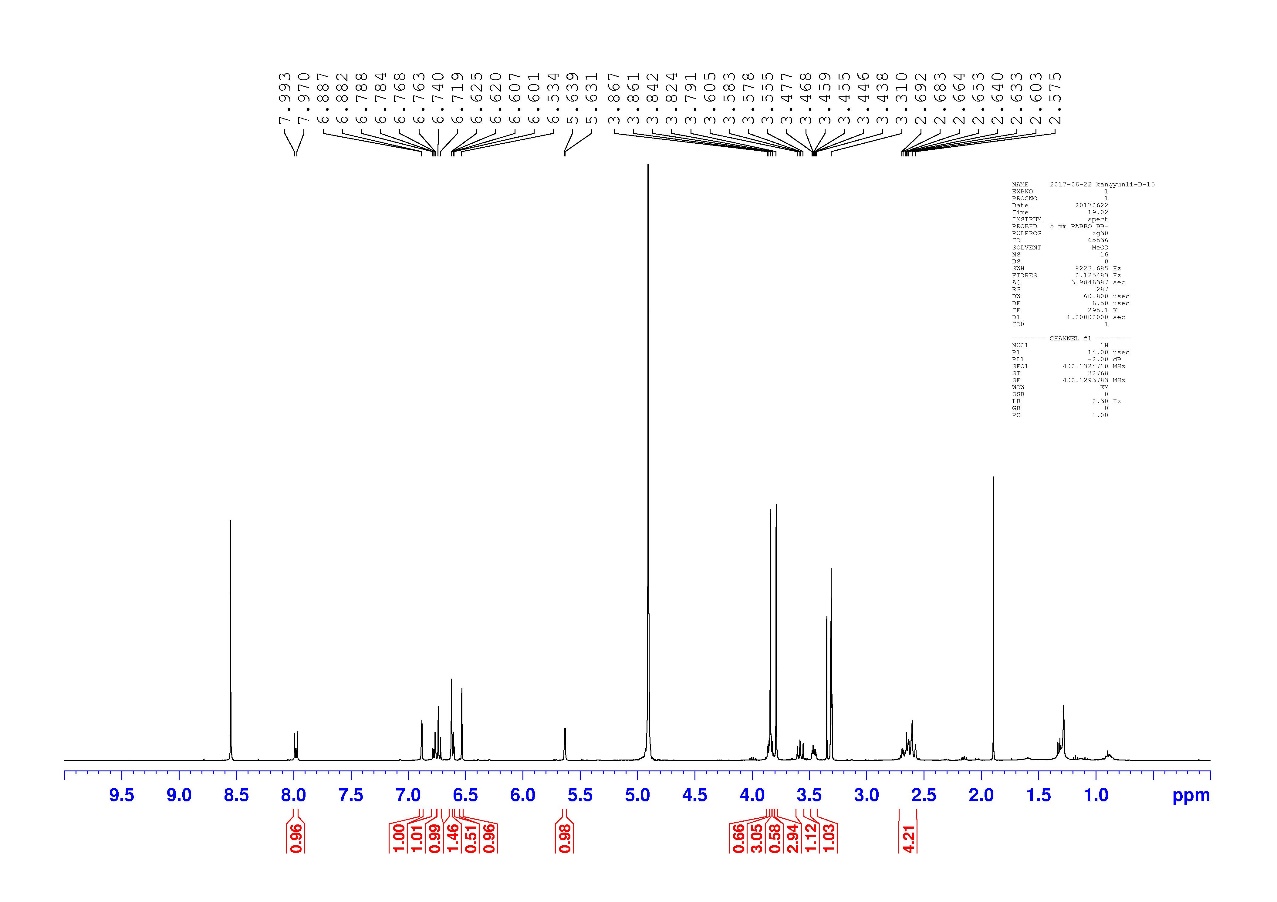


^1^H NMR (400 MHz, CD_3_OD) spectrum of compound **17**


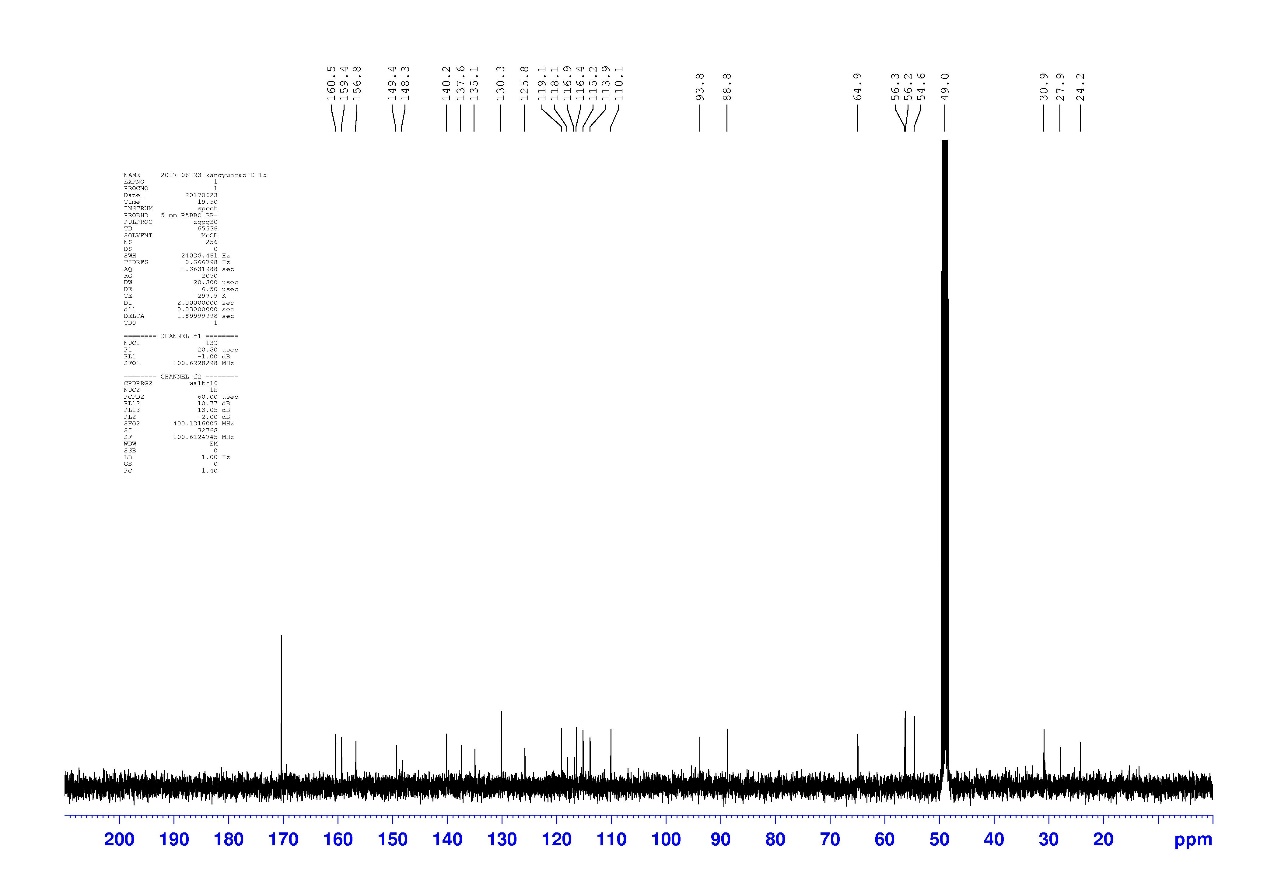


^13^C NMR (100 MHz, CD_3_OD) spectrum of compound **17**

**
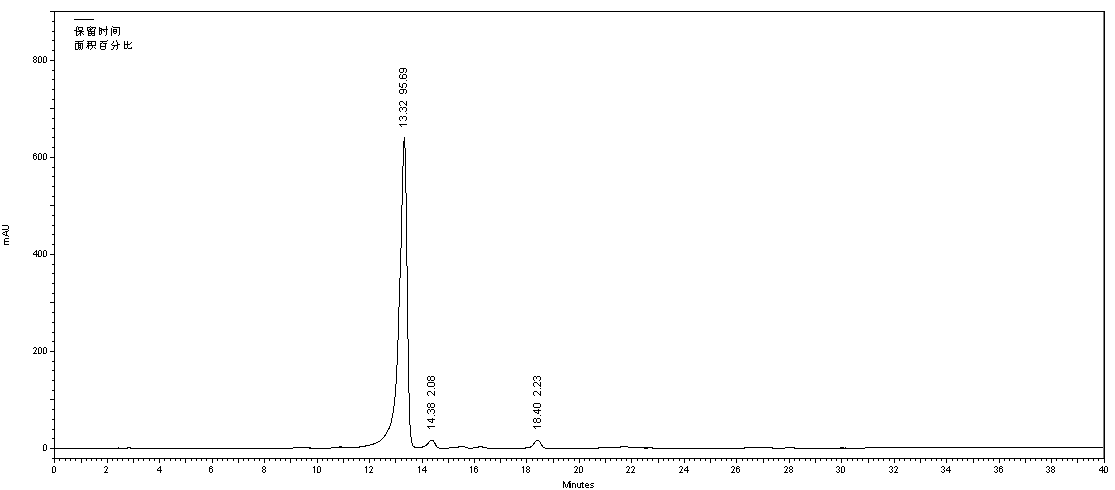
**

HPLC chromatogram of compound **17**

HR-ESI-MS spectrum of **18**


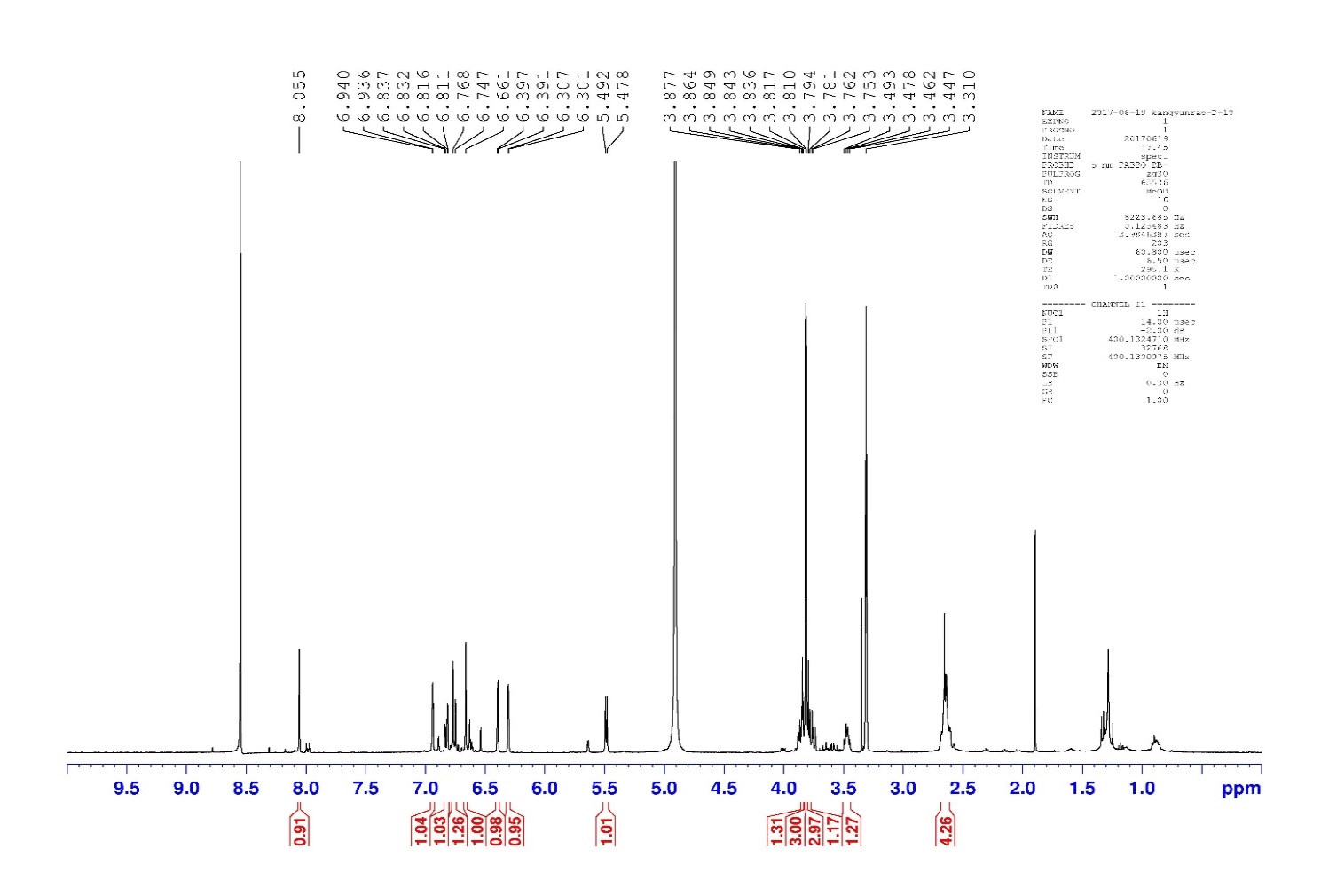


^1^H NMR (400 MHz, CD_3_OD) spectrum of **18**


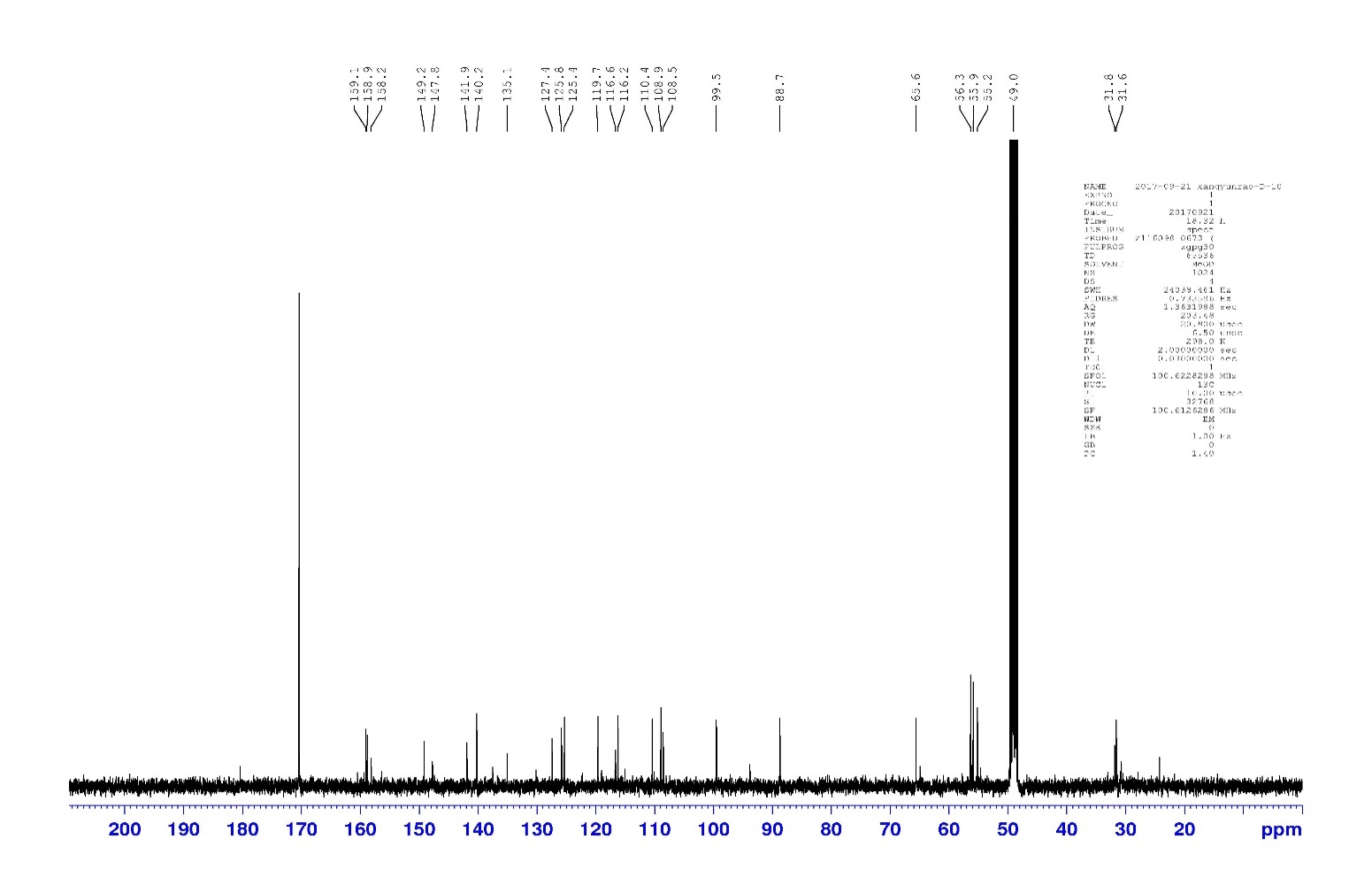


^13^C NMR (100 MHz, CD_3_OD) spectrum of **18**


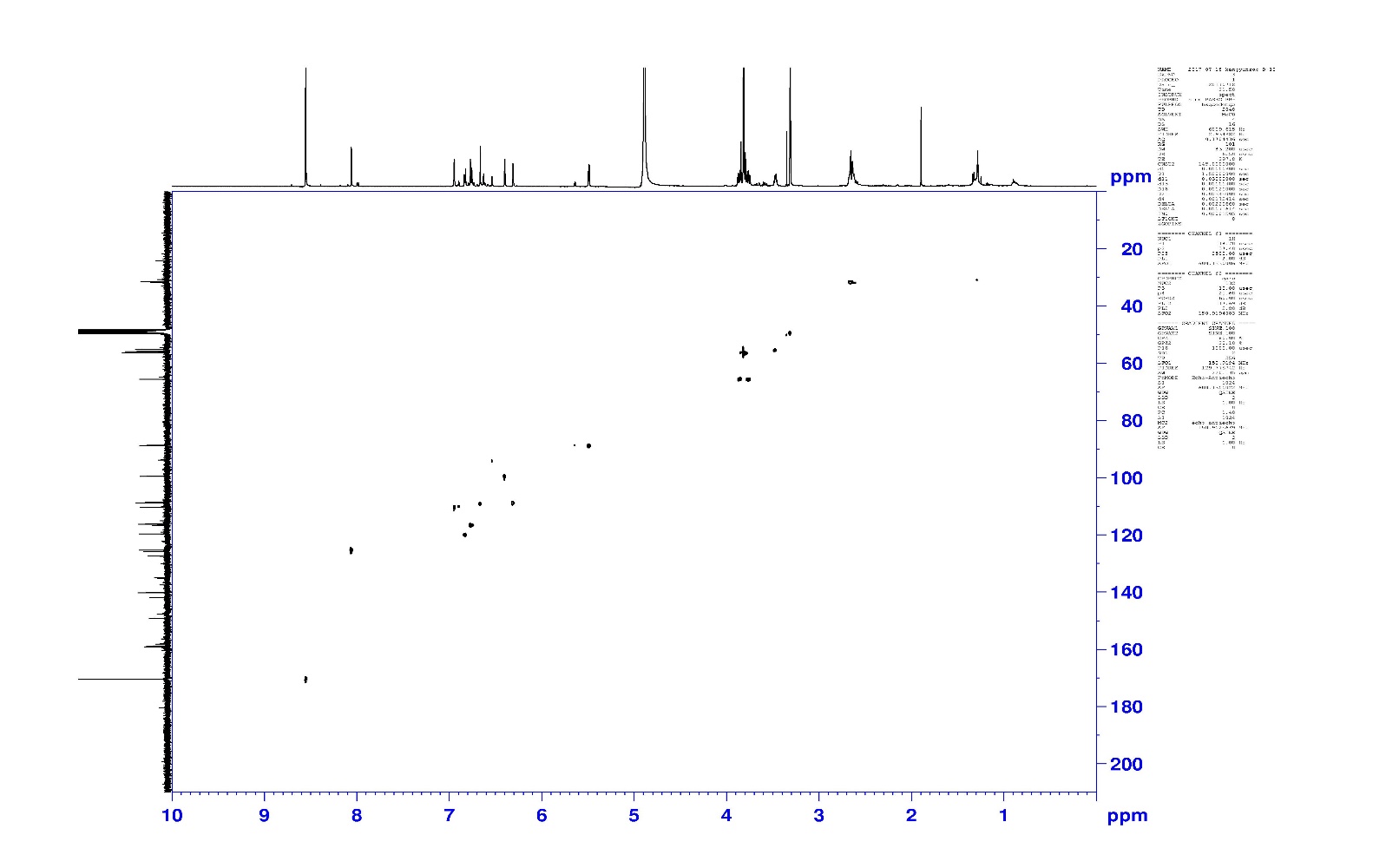


HSQC spectrum of **18**.


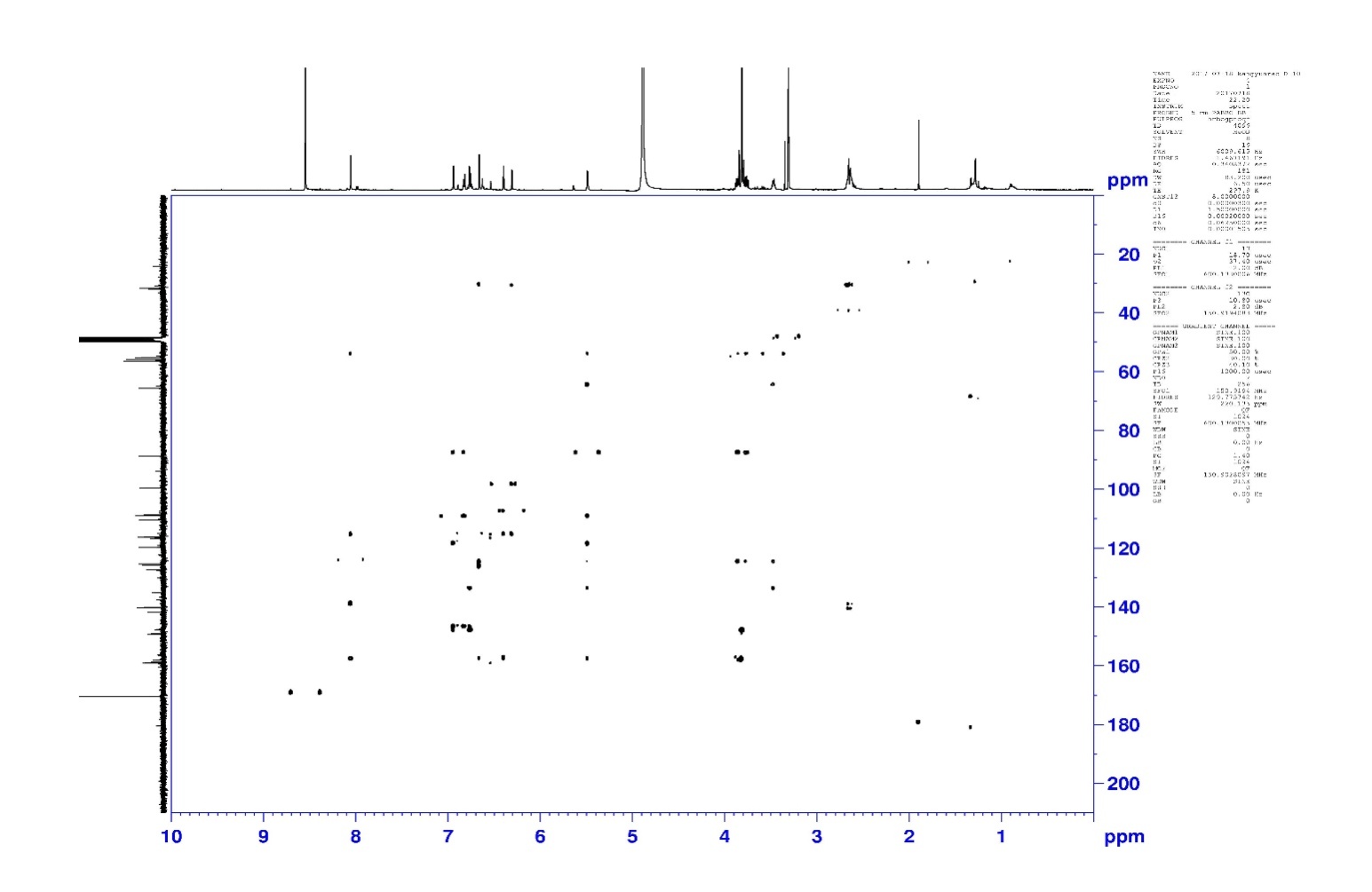


HMBC spectrum of **18**


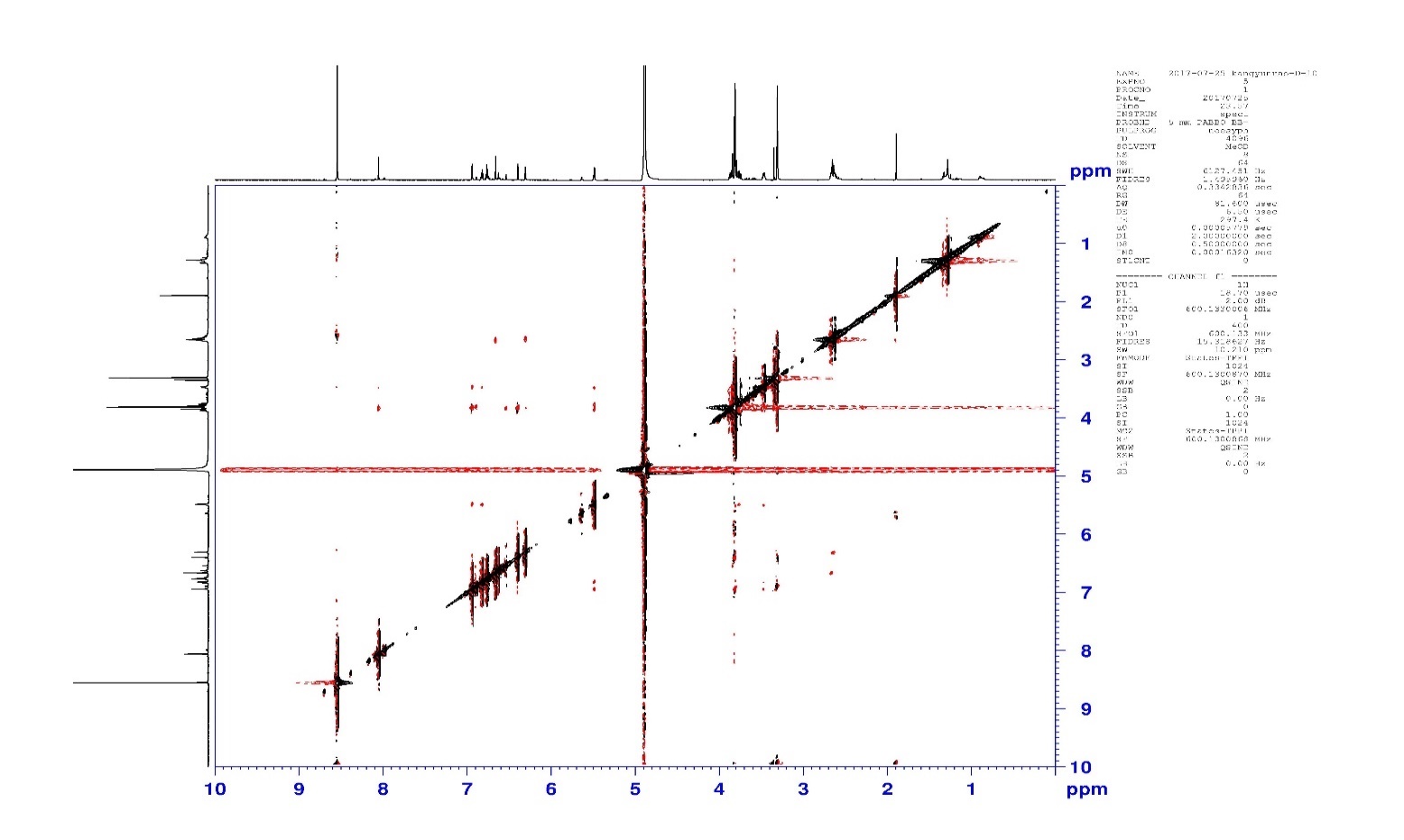


NOESY spectrum of **18**

**
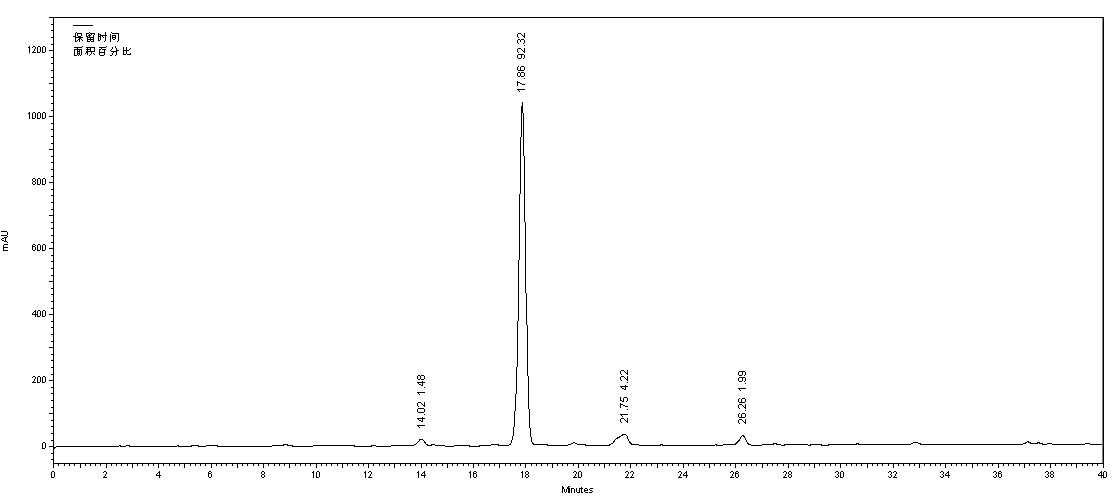
**

HPLC chromatogram of compound **18**

HR-ESI-MS spectrum of compound **19**


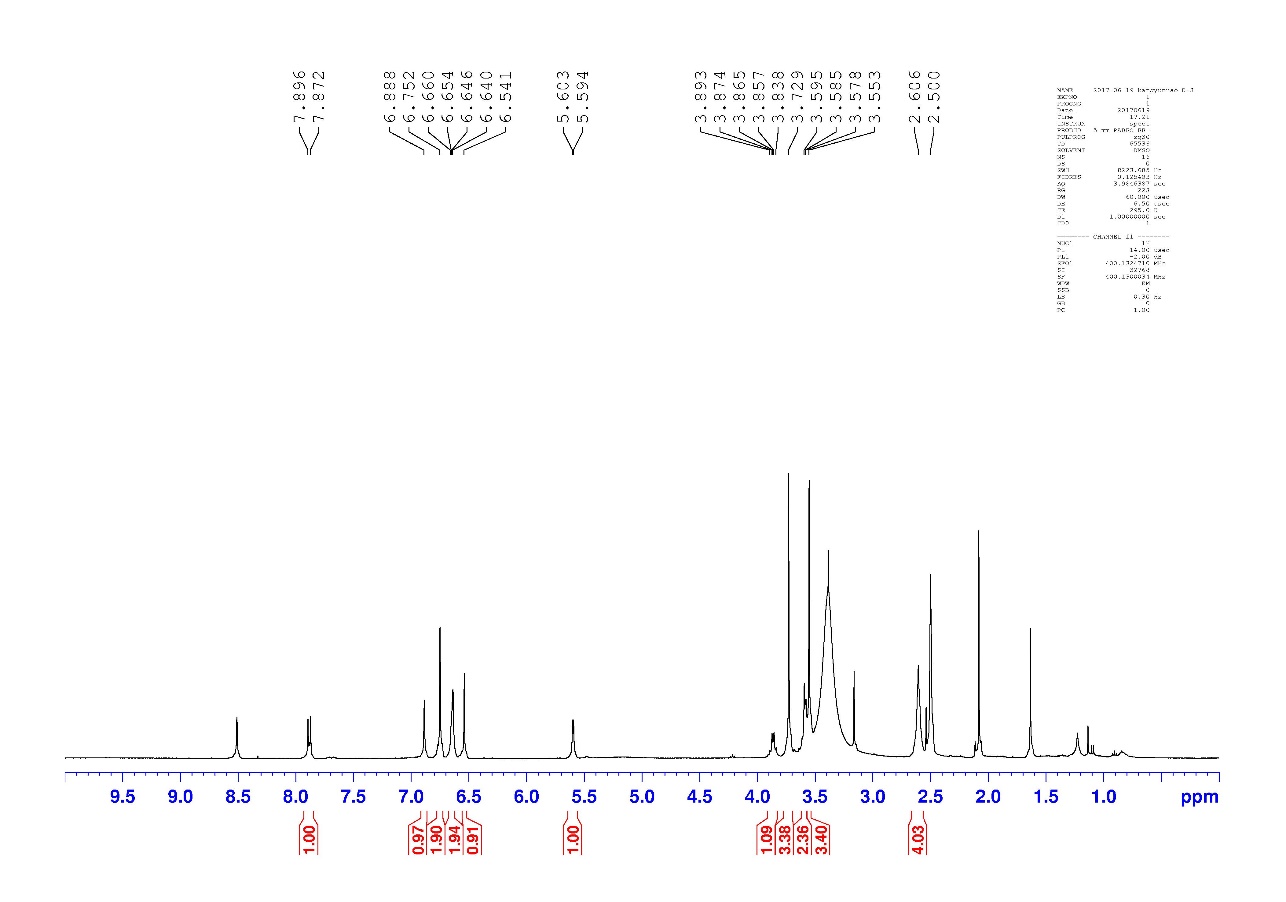


^1^H NMR (400 MHz, DMSO-*d*_6_) spectrum of compound **19**


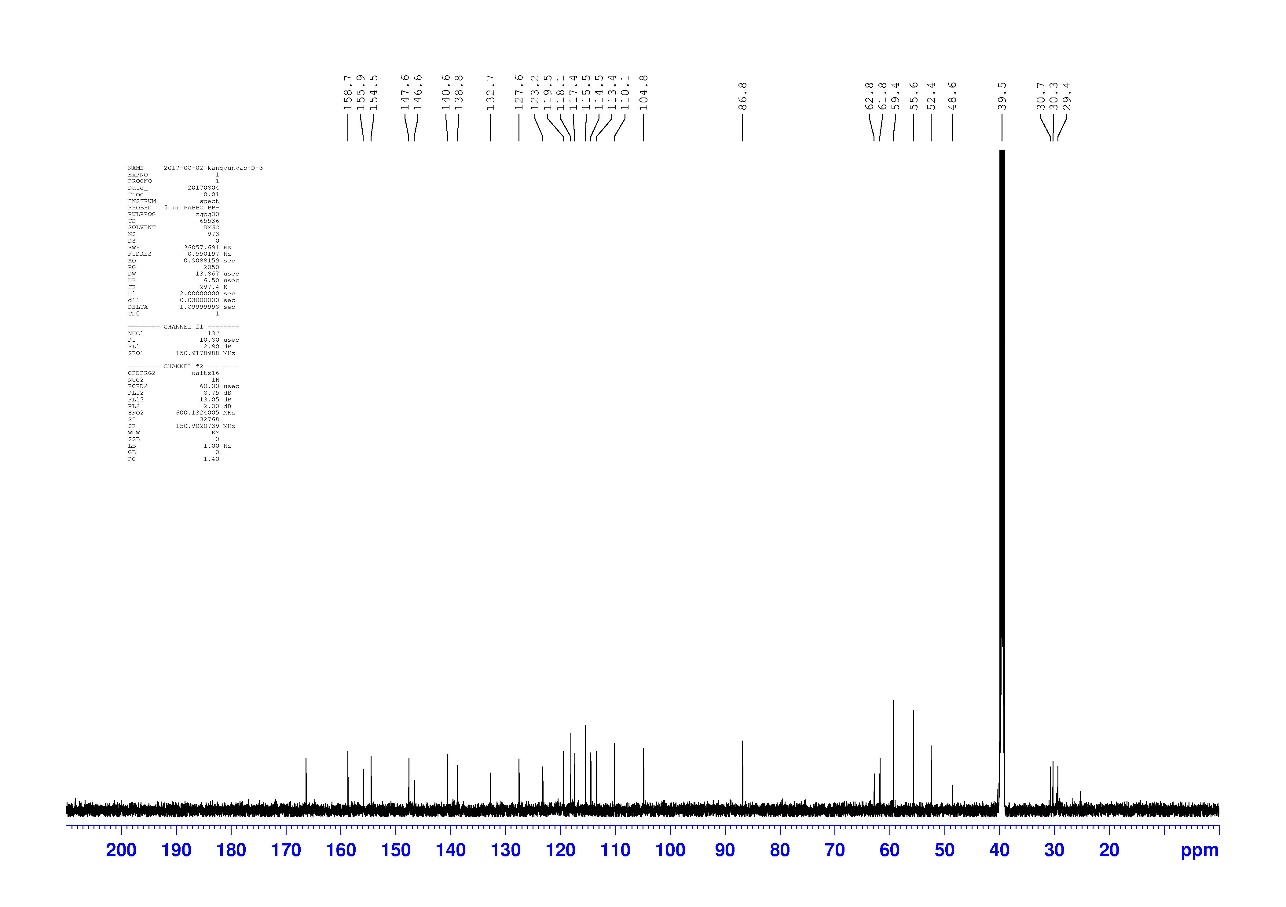


^13^C NMR (100 MHz, DMSO-*d*_6_) spectrum of compound **19**


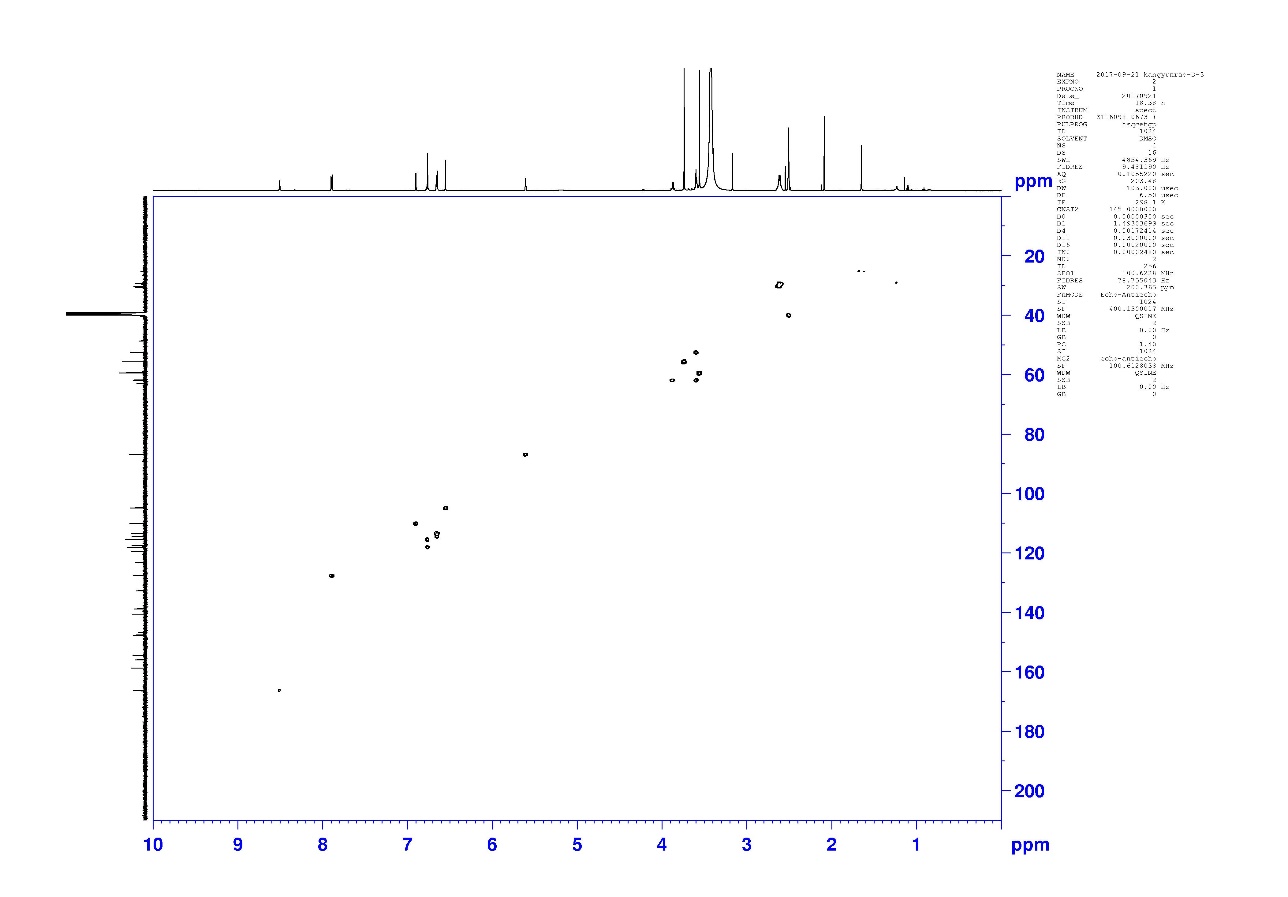


HSQC spectrum of compound **19**


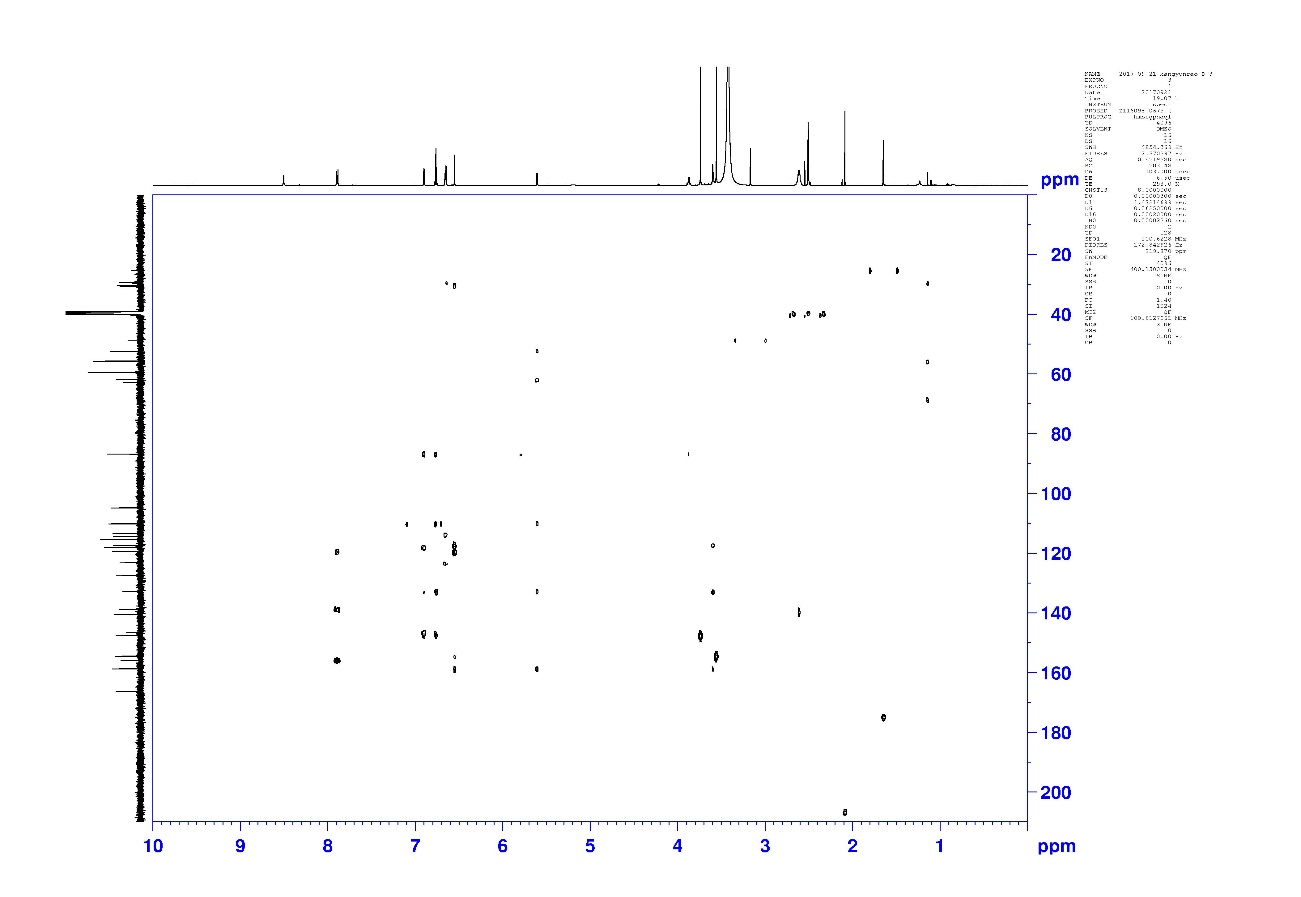


HMBC spectrum of compound **19**


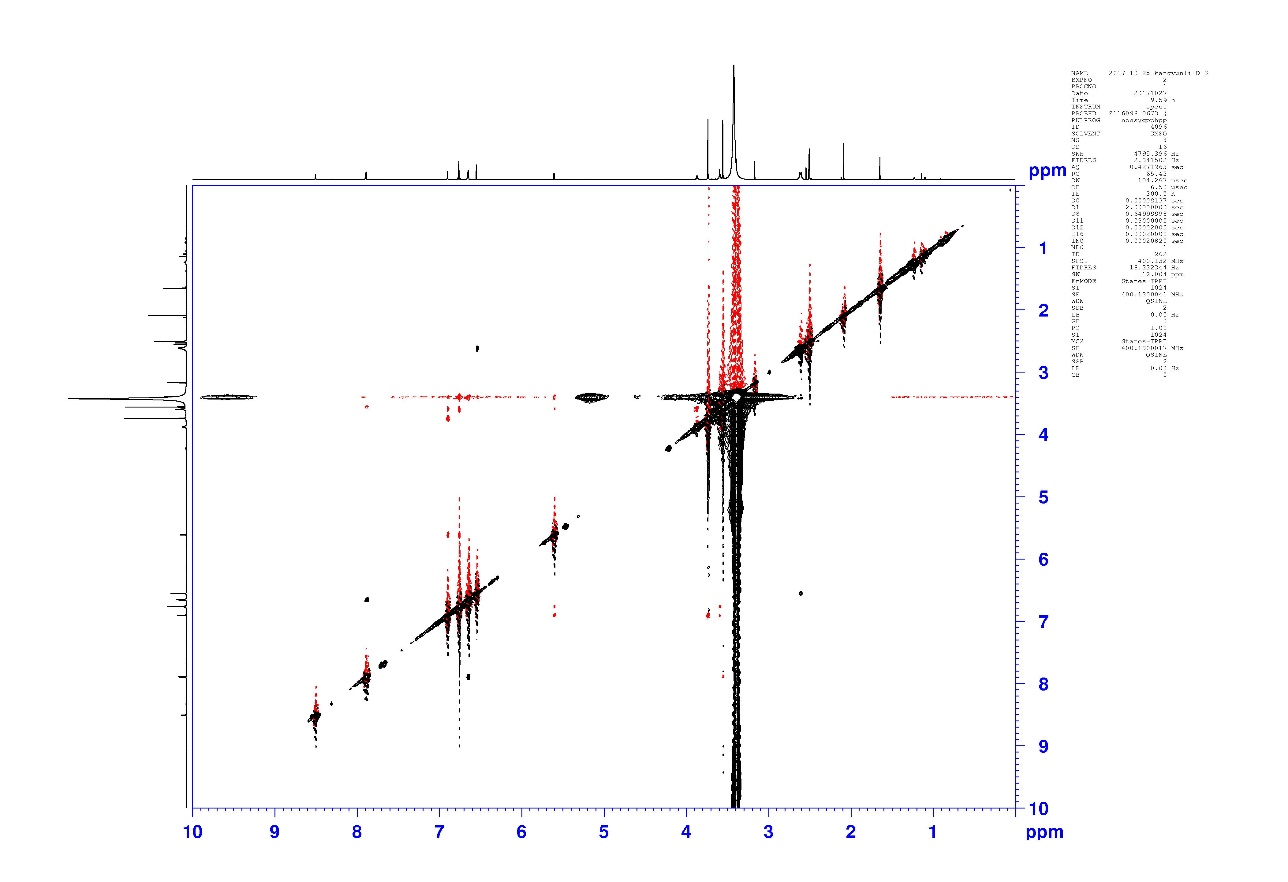


NOESY spectrum of compound **19**

**
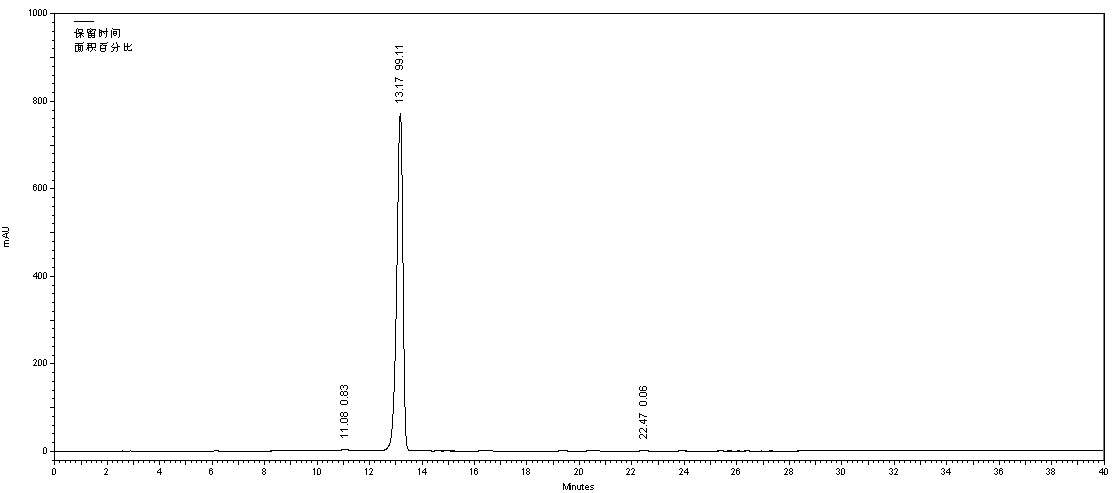
**

HPLC chromatogram of compound **19**


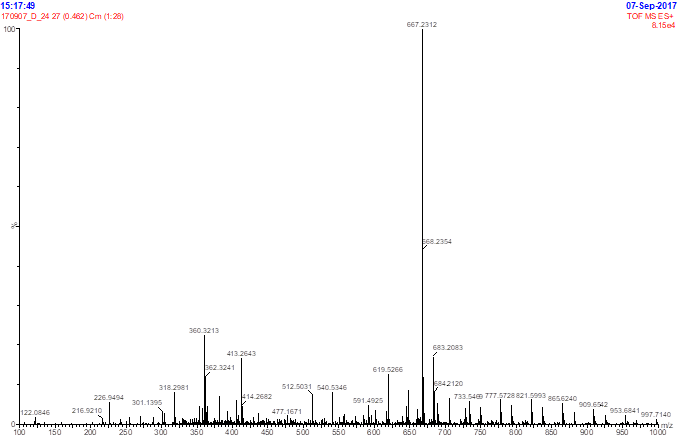


HR-ESI-MS spectrum of compound 20.


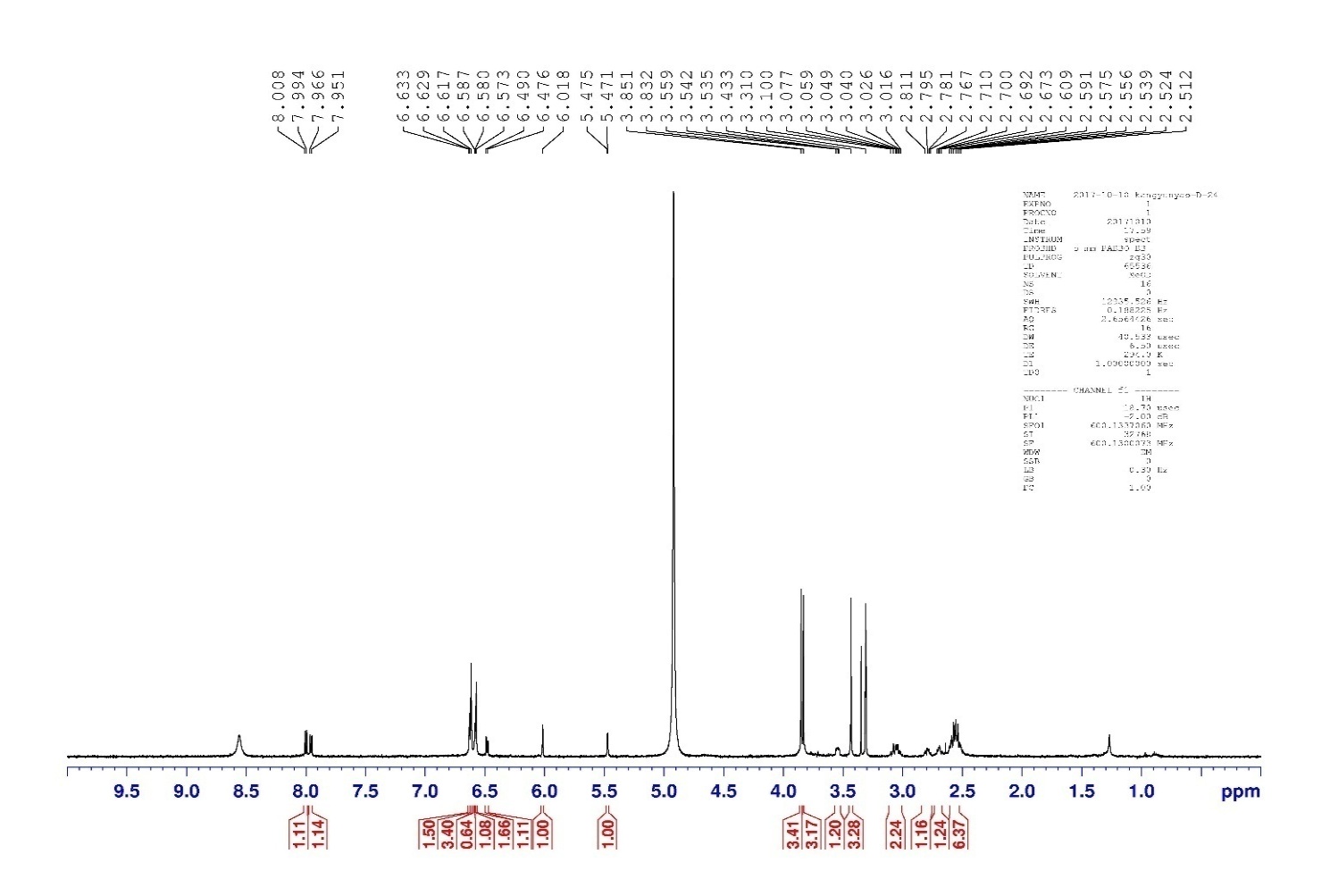


^1^H NMR (400 MHz, CD_3_OD) spectrum of compound **20**


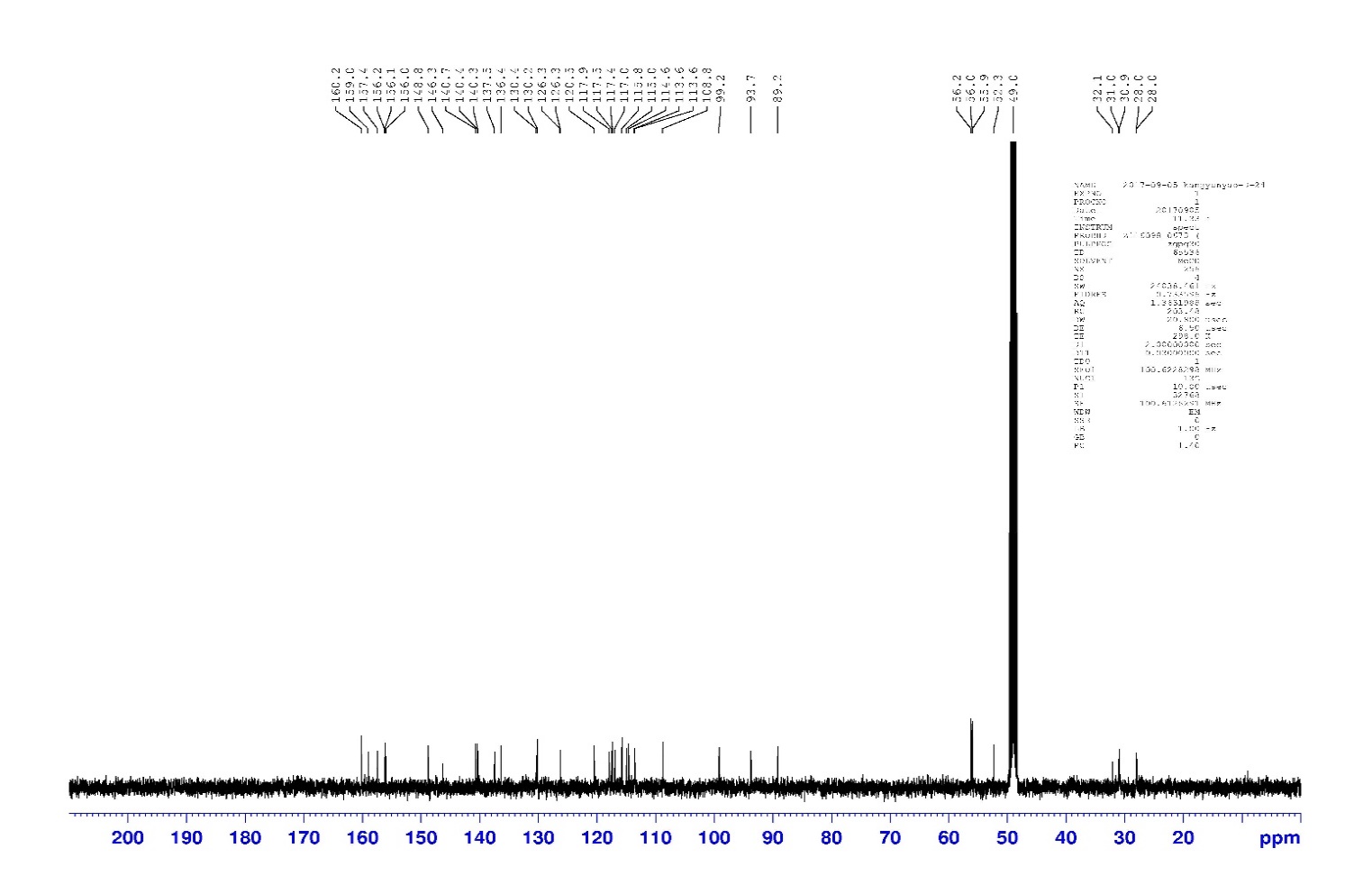


^13^C NMR (100 MHz, CD_3_OD) spectrum of compound **20**.


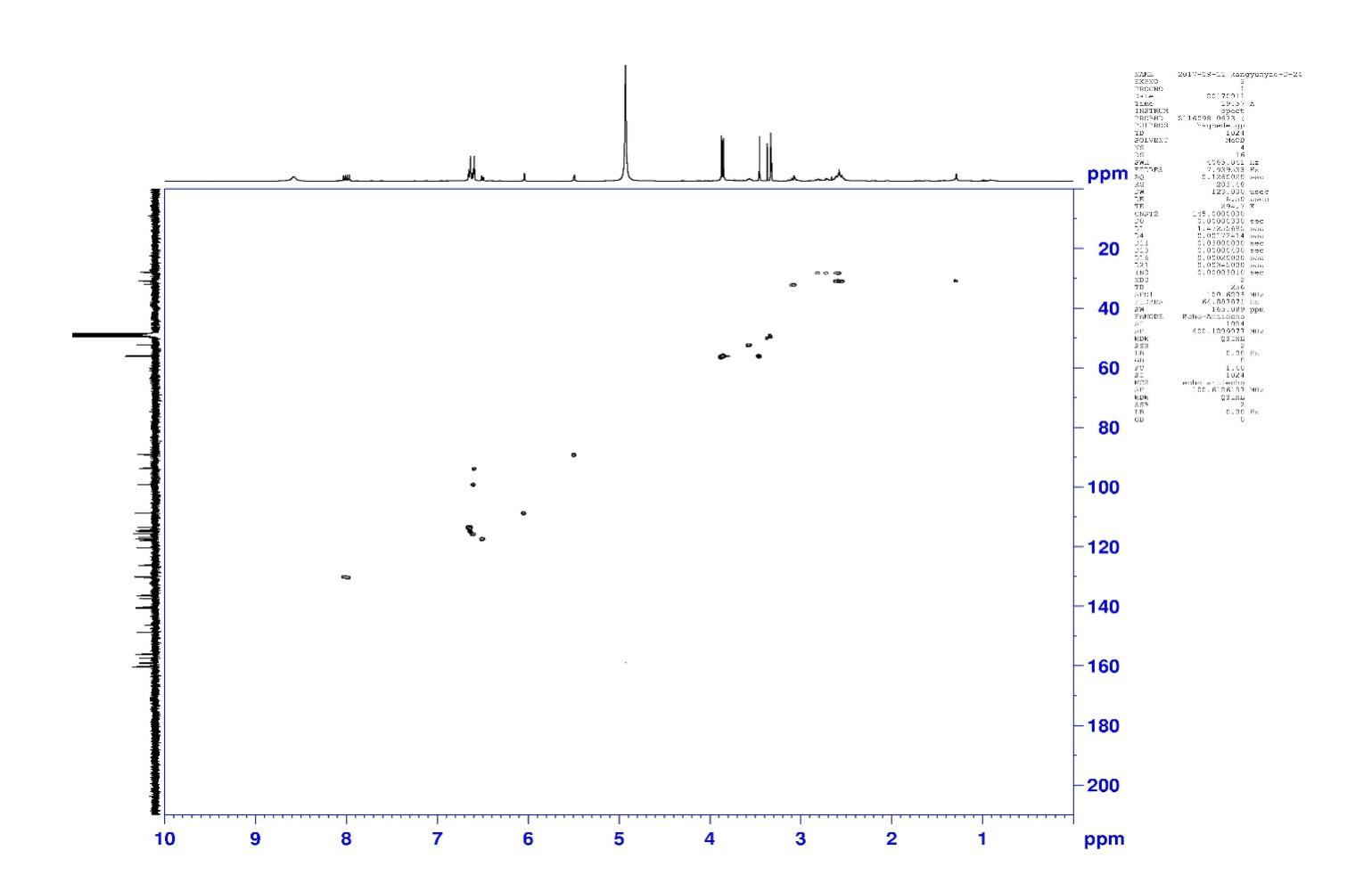


HSQC spectrum of compound **20**.


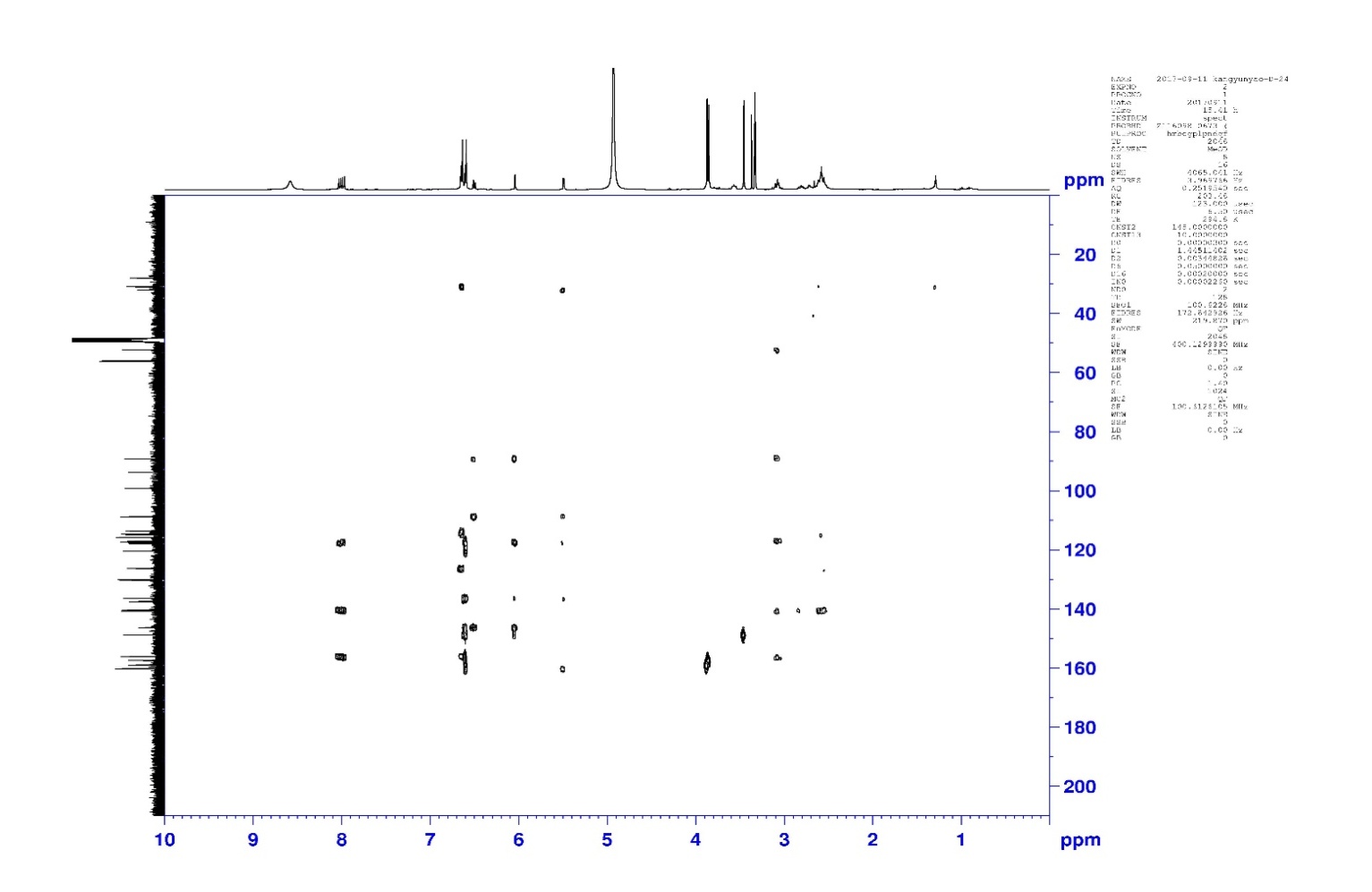


HMBC spectrum of compound **20**


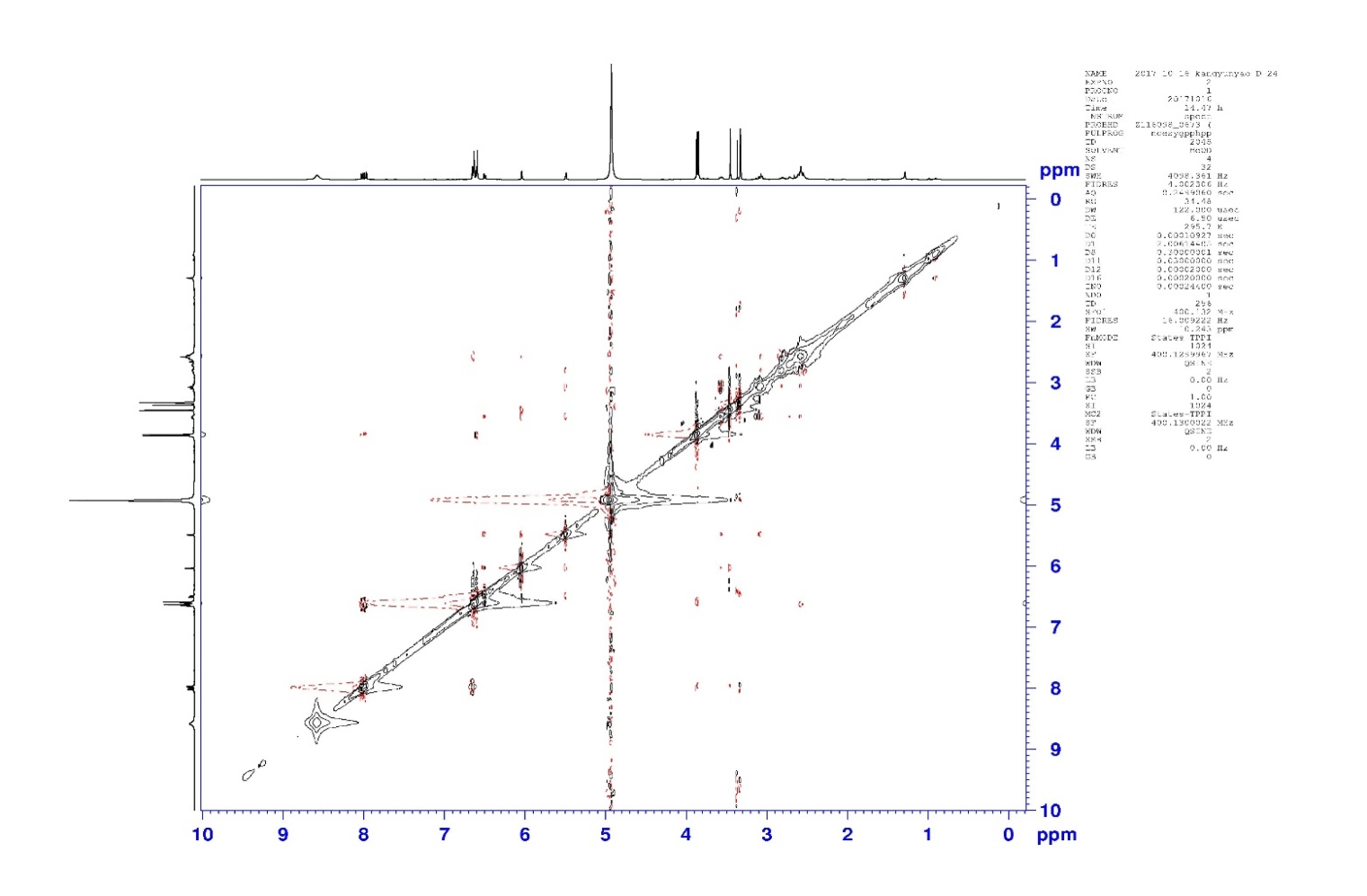


NOESY spectrum of compound **20**.

**
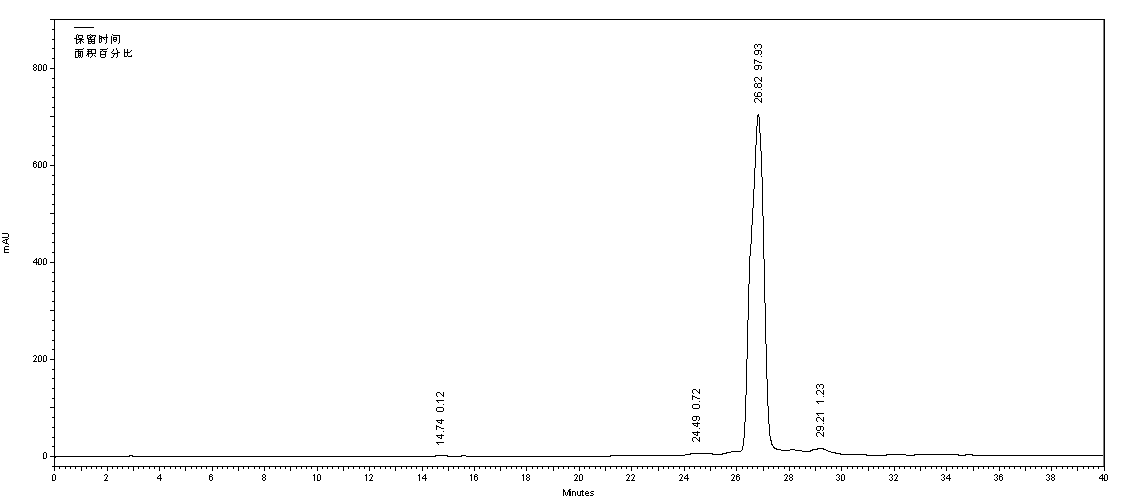
**

HPLC chromatogram of compound **20**

HR-ESI-MS spectrum of compound **21**


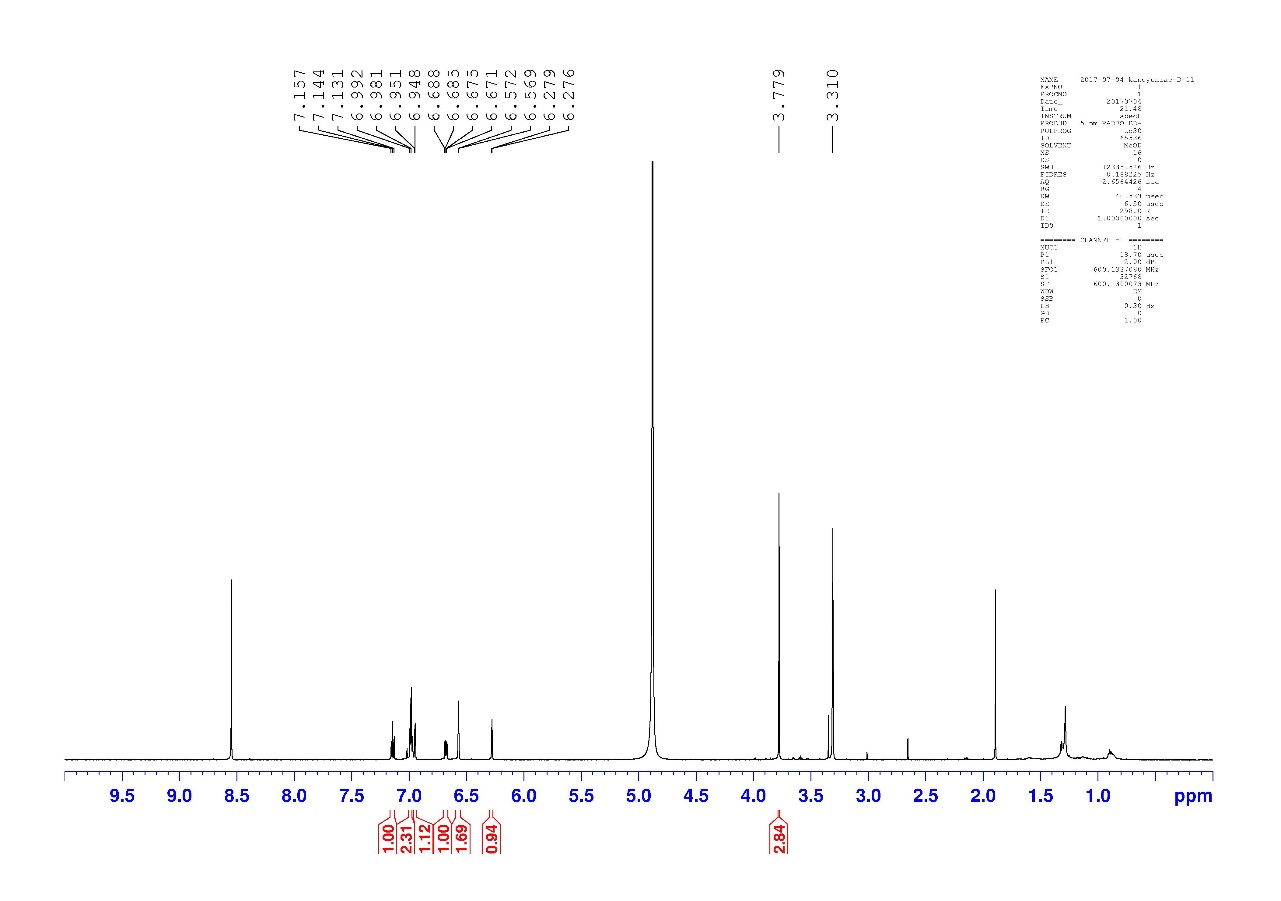


^1^H NMR (400 MHz, CD_3_OD) spectrum of compound **21**


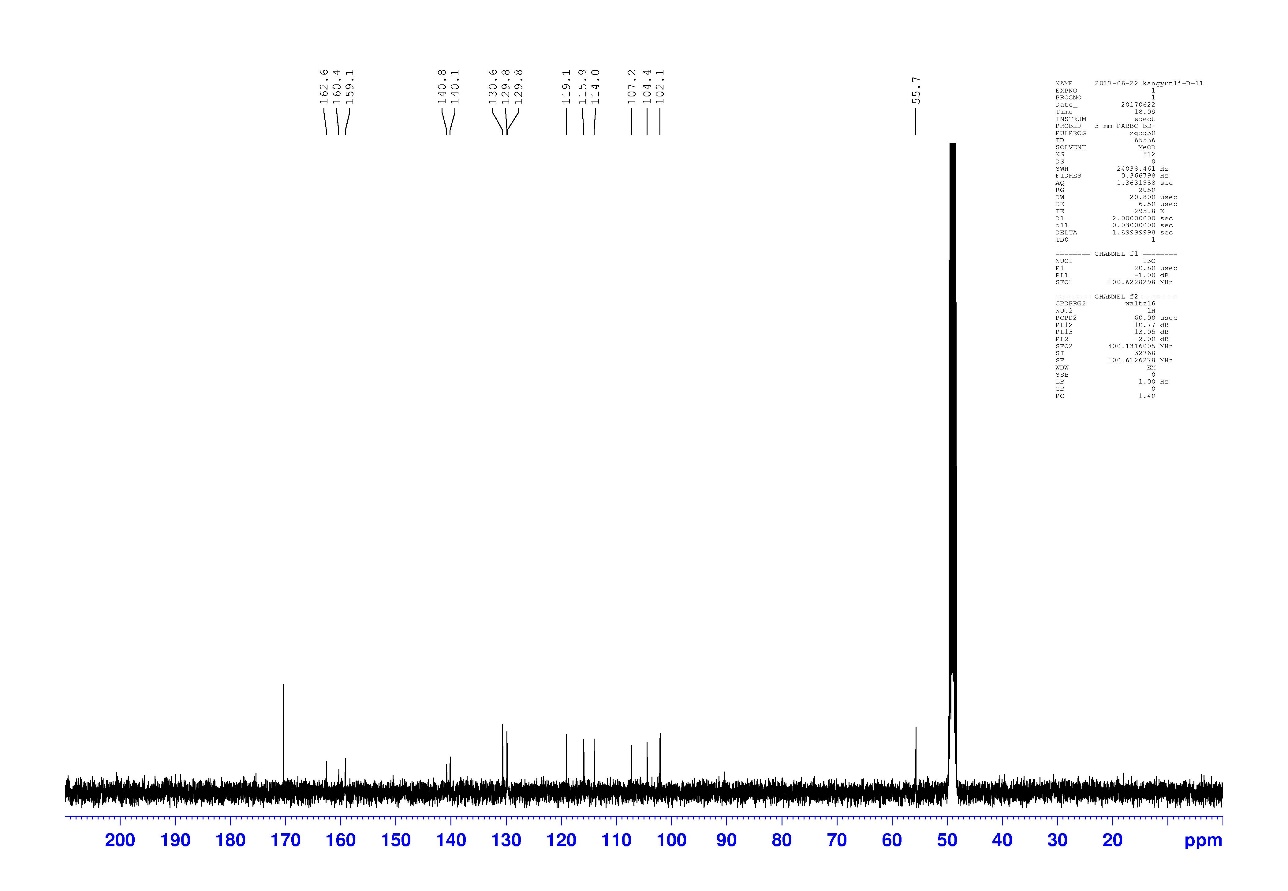


^13^C NMR (100 MHz, CD_3_OD) spectrum of compound **21**

**
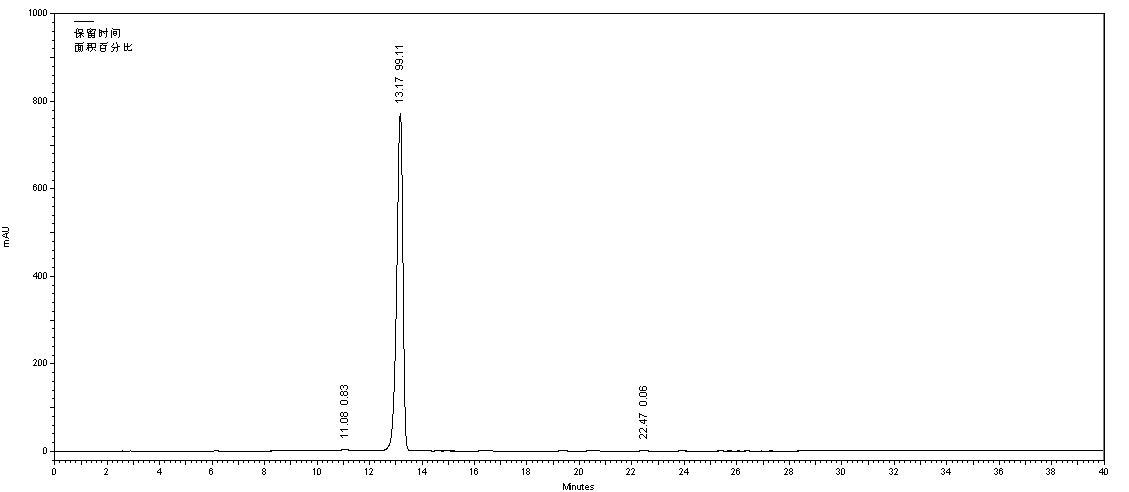
**

HPLC chromatogram of compound **21**

HR-ESI-MS spectrum of compound **22**


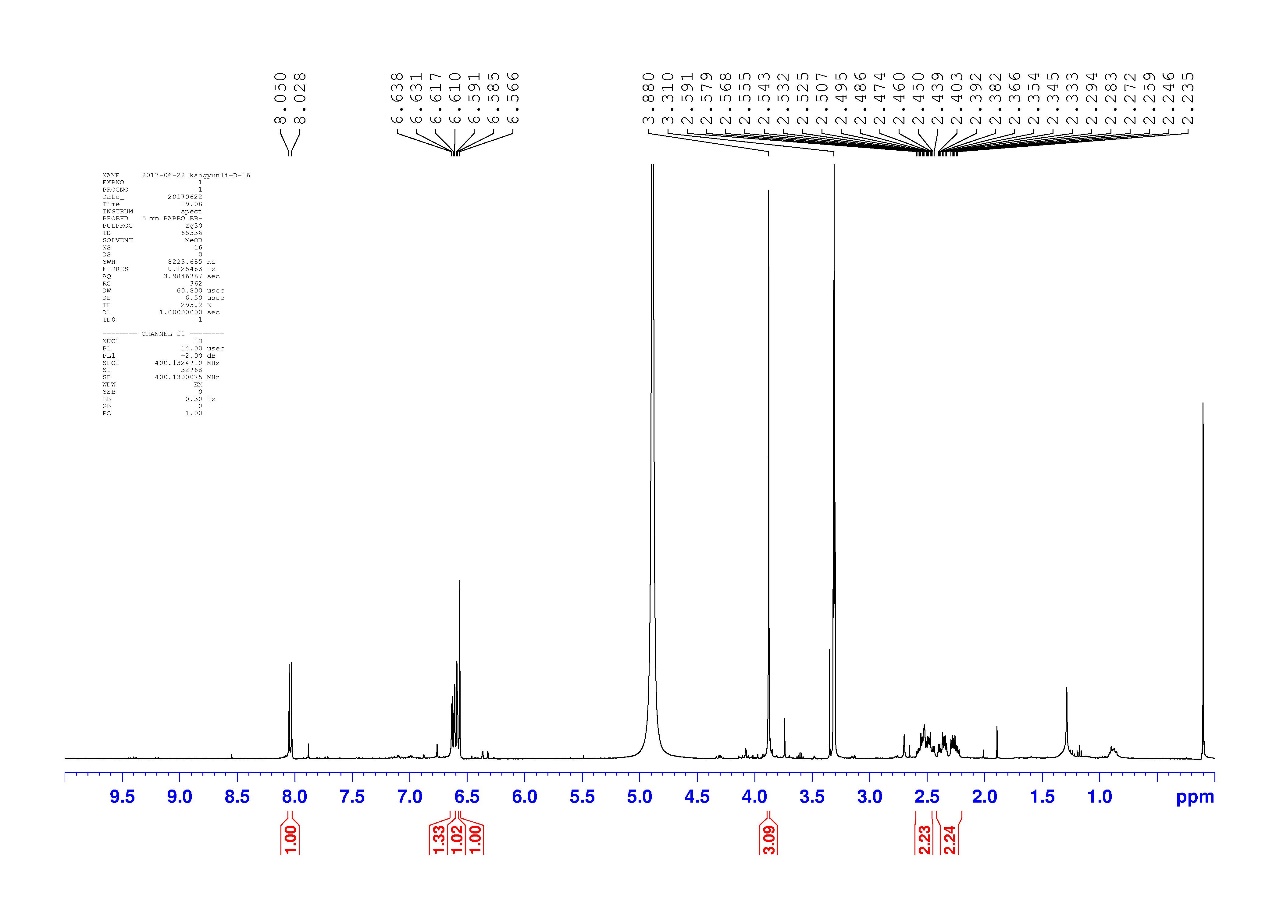


^1^H NMR (400 MHz, CD_3_OD)spectrum of compound **22**


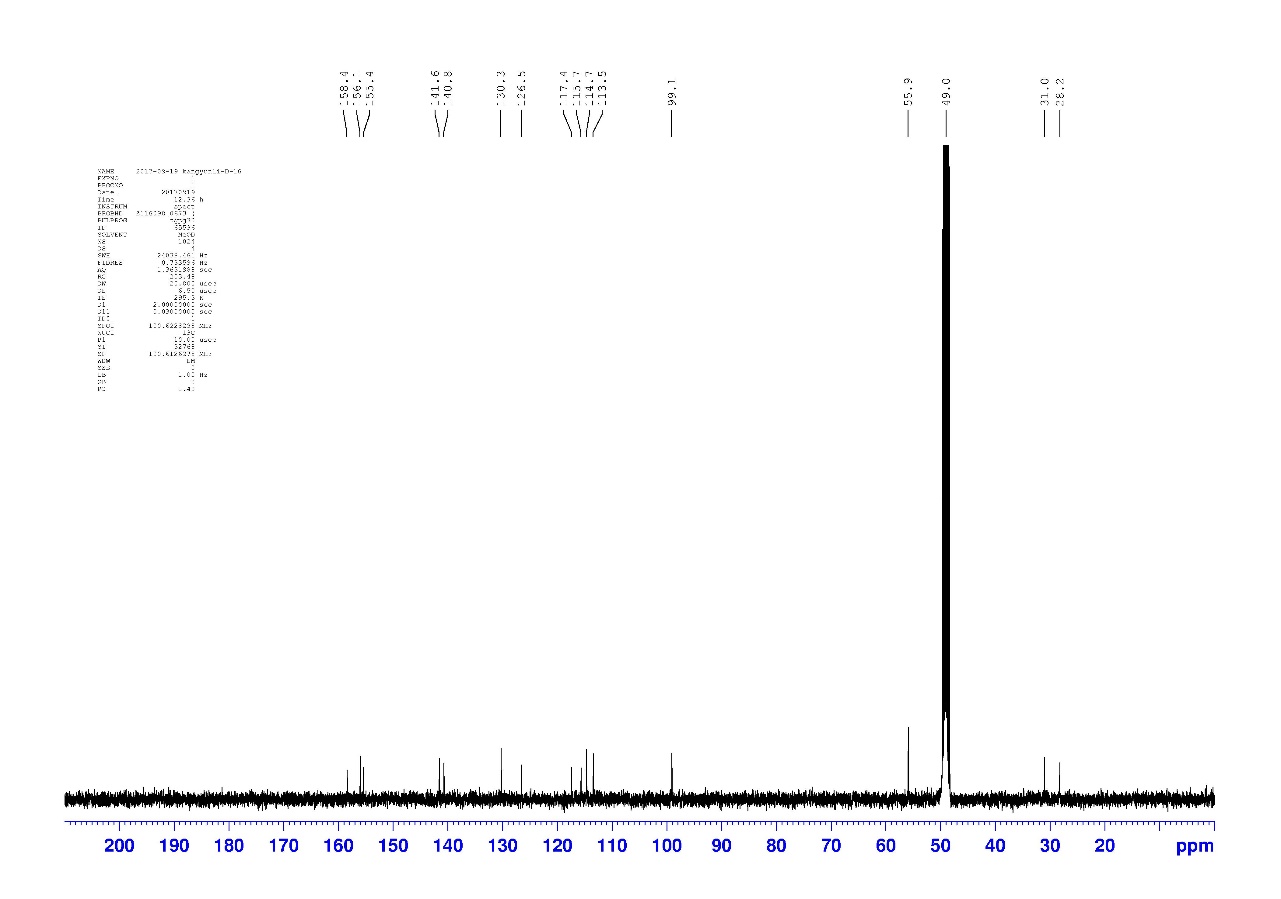


^13^C NMR (100 MHz, CD_3_OD) spectrum of compound **22**


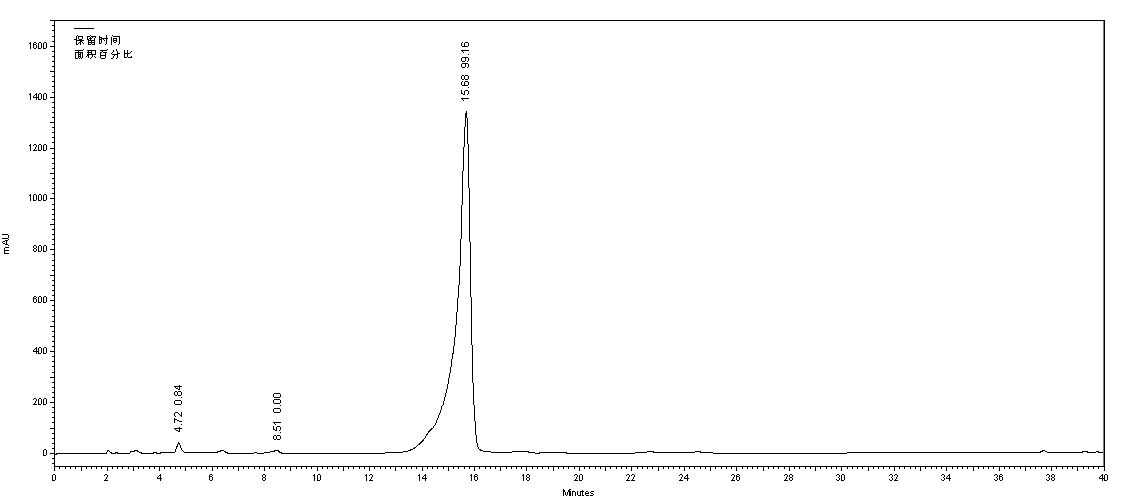


HPLC chromatogram of compound **22**
